# Conserved differences in protein sequence determine the human pathogenicity of Ebolaviruses

**DOI:** 10.1038/srep23743

**Published:** 2016-03-24

**Authors:** Morena Pappalardo, Miguel Juliá, Mark J. Howard, Jeremy S. Rossman, Martin Michaelis, Mark N. Wass

**Affiliations:** 1 Centre for Molecular Processing and School of Biosciences, University of Kent, Canterbury, Kent CT2 7NJ, UK

## Abstract

Reston viruses are the only Ebolaviruses that are not pathogenic in humans. We analyzed 196 Ebolavirus genomes and identified specificity determining positions (SDPs) in all nine Ebolavirus proteins that distinguish Reston viruses from the four human pathogenic Ebolaviruses. A subset of these SDPs will explain the differences in human pathogenicity between Reston and the other four ebolavirus species. Structural analysis was performed to identify those SDPs that are likely to have a functional effect. This analysis revealed novel functional insights in particular for Ebolavirus proteins VP40 and VP24. The VP40 SDP P85T interferes with VP40 function by altering octamer formation. The VP40 SDP Q245P affects the structure and hydrophobic core of the protein and consequently protein function. Three VP24 SDPs (T131S, M136L, Q139R) are likely to impair VP24 binding to human karyopherin alpha5 (KPNA5) and therefore inhibition of interferon signaling. Since VP24 is critical for Ebolavirus adaptation to novel hosts, and only a few SDPs distinguish Reston virus VP24 from VP24 of other Ebolaviruses, human pathogenic Reston viruses may emerge. This is of concern since Reston viruses circulate in domestic pigs and can infect humans, possibly via airborne transmission.

Four of the five members of the genus *Ebolavirus* (Ebola viruses, Sudan viruses, Bundibugyo viruses, Taϊ Forest viruses) cause hemorrhagic fever in humans associated with fatality rates of up to 90% while Reston viruses are non-pathogenic to humans^1^,^2^ (see Materials and Methods for the *Ebolavirus* nomenclature). So far there have been three Reston virus outbreaks in nonhuman primates: 1989–1990 in Reston Virginia, USA, 1992–1993 in Sienna, Italy, and 1996 in a licensed commercial quarantine facility in Texas. All cases were traced back to a single monkey breeding facility in the Philippines. During these outbreaks five human individuals were tested positive for IgG antibodies directed against Reston virus. Moreover, Reston virus was found in 2008 in domestic pigs in the Philippines. Seroconversion was detected in six human individuals. None of the 11 individuals that were seropositive for Reston virus antibodies reported an Ebola-like disease^3^.

The reasons underlying the differences in human pathogenicity between Reston viruses and the members of the other *Ebolavirus* species remain unclear. Understanding of the molecular causes of these differences would enhance our understanding of Ebolavirus function and pathogenicity and aid investigation into treatment of Ebolavirus infection. Here, we performed an *in silico* analysis of the genomic differences between Reston viruses and human pathogenic Ebolaviruses to identify conserved changes at the protein level that explain the differences in Ebolavirus pathogenicity in humans.

Ebolaviruses encode nine proteins including nucleoprotein (NP), glycoprotein (GP), soluble GP (sGP), small soluble GP (ssGP), RNA dependent RNA polymerase (L), and four structural proteins termed VP24, VP30, VP35, and VP40^1^,^4^,^5^. GP, sGP, and ssGP are produced from the *GP* gene by alternative RNA editing^1^,^4^,^5^. Many of the Ebolavirus proteins have multiple functions. In the virion, the NP-encapsulated RNA genome associates with VP35, VP30, and L to form the transcriptase-replicase complex. VP35 and VP24, a membrane-associated structural protein, antagonize the cellular interferon response. The matrix protein VP40 fulfills critical roles during virus assembly and release. GP, the only transmembrane surface protein, is responsible for host cell binding and virus internalization^1^,^6^. Little is known about the functional roles of the secreted proteins sGP and ssGP^1^,^3^,^4^,^7^.

Despite the small Ebolavirus genome we still have a limited understanding of Ebolaviruses and what causes their pathogenicity and why Reston viruses are not human pathogenic^1^,^6^,^8^. The importance of understanding these differences is highlighted by the current Ebola virus outbreak in Western Africa, which is the first large outbreak and has resulted in 27,345 suspected cases and 11,184 deaths to date (www.who.int, as of 14^th^ June 2015). During this outbreak many additional Ebola virus genomes were sequenced enabling us to perform the first comprehensive comparison of the non-human pathogenic Reston virus to all four human pathogenic Ebolaviruses. While some studies^8^,^9^,^10^ have compared the differences between individual Reston virus proteins derived from a certain strain with their equivalent derived from one strain of a human pathogenic species, none have performed a systematic analysis of all available protein sequence information from all (known) *Ebolavirus* species.

Our large scale analysis of nearly 200 different Ebolavirus genomes focussed on combining computational methods with detailed structural analysis to identify the genetic causes of the difference in pathogenicity between Reston viruses and the human pathogenic *Ebolavirus* species. Central to our approach was the identification of Specificity Determining Positions (SDPs), which are positions in the proteome that are conserved within protein subfamilies but differ between them^11^,^12^ and thus distinguish between the different functional specificities of proteins from the different *Ebolavirus* species. SDPs have been demonstrated to be typically associated with functional sites, such as protein-protein interface sites and enzyme active sites^12^. The SDPs that we have identified and that distinguish Reston viruses from human pathogenic Ebolaviruses, arguably, contain within them a set of amino acid changes that explain the differences in pathogenicity between Reston viruses and the four human pathogenic species, although a contribution of non-coding RNAs (that may exist but remain to be detected) cannot be excluded^6^,^13^. The subsequent structural analysis was performed to identify the SDPs that are most likely to affect Ebolavirus pathogenicity, using an approach that is similar to those used to investigate candidate single nucleotide variants in human genome wide association and sequencing studies by us and others^14^,^15^,^16^,^17^.

## Results

### Specificity Determining Positions (SDP) Analysis

**Figure 1:**
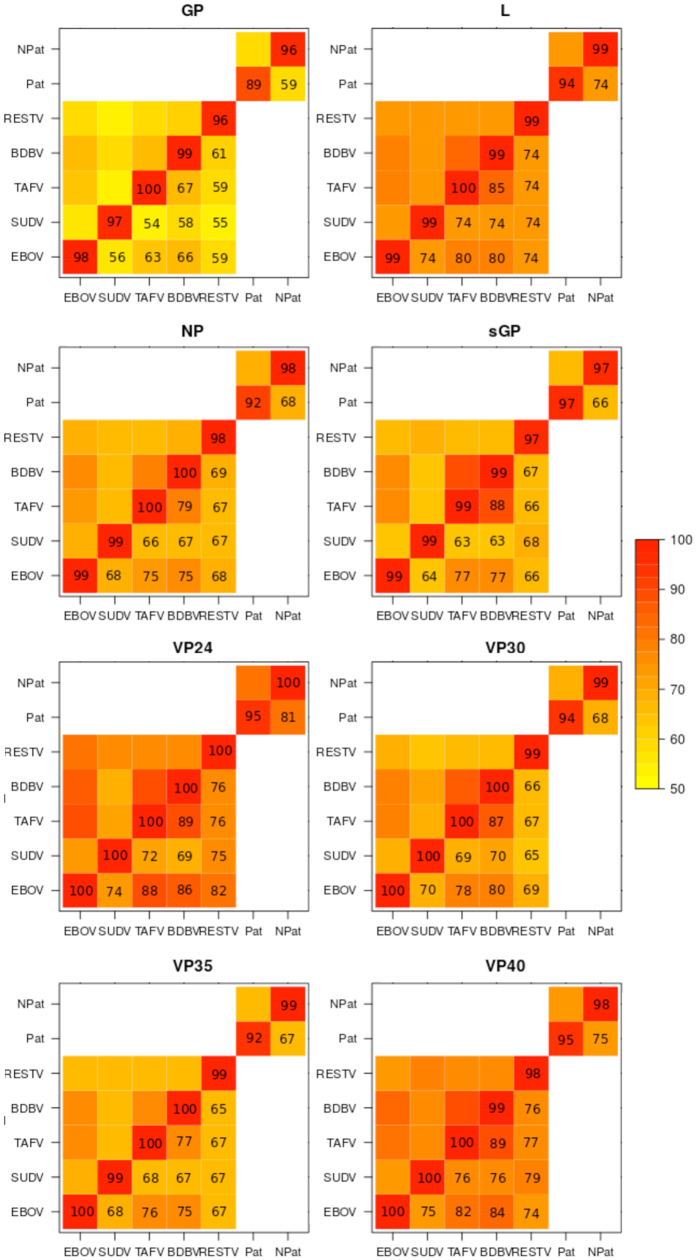
Conservation of Ebolavirus proteins. Heatmaps of intra- and inter species sequence identity for Ebolavirus proteins. (EBOV, Ebola virus; BDBV, Bundibugyo virus; SUDV, Sudan virus; TAFV, Taϊ Forest virus; RESTV, Reston virus).

Ebolavirus genomes were obtained from the Virus Pathogen Resource (ViPR^18^), consisting of 156 Ebola viruses, 7 Bundibugyo viruses, 13 Sudan viruses, 3 Taϊ Forest viruses, and 17 Reston viruses (online Methods). Phylogenetic analysis of the whole genomes and the individual proteins separated the *Ebolavirus* species from each other (Supplementary Figure 1). In accordance with previous studies^19^,^20^,^21^,^22^,^23^, we observed high intra-species conservation with greater inter-species variation (Fig. 1 and Supplementary Table 1). The surface protein GP exhibited the greatest variation (Fig. 1), most likely as a consequence of selective pressure exerted by the host immune response^21^.

**Figure 2:**
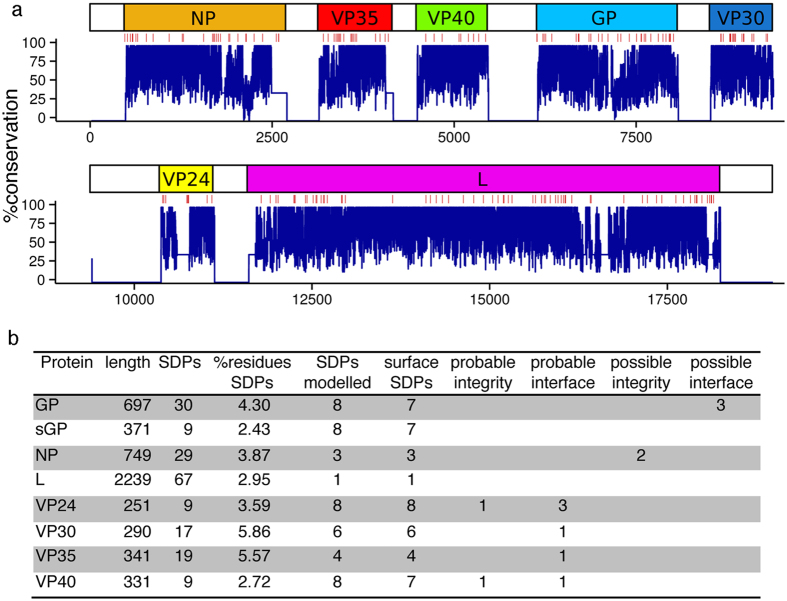
Ebolavirus SDPs. (**a**) genomic overview of Ebolavirus conservation. SDPs are shown as red lines with protein conservation (blue graph). (**b**) The number of SDPs in each of the Ebolavirus proteins is shown with details on: the number of SDPs that were mapped onto protein structures and the numbers that were identified to have potential roles in changing pathogenicity by either affecting protein-protein interactions (interface) or changing protein structure-function. These changes were classed as probable, where there is high confidence of the effect and possible where there is a lower level of confidence in the observations.

Using the S3Det algorithm^12^ (Materials and Methods), we identified 189 SDPs that are differentially conserved between Reston viruses and human pathogenic Ebolaviruses (Fig. 2, Supplementary Figure 2, Supplementary Tables 2–9). These SDPs represent the most significant changes between the Reston virus and the human pathogenic Ebolaviruses so a subset of these SDPs must explain the difference in pathogenicity. SDPs were present in each of the Ebolavirus proteins representing between 2.4% of residues in sGP to 5.9% of residues in VP30 (Fig. 2b). Comparison of the SDPs with previously published mutagenesis studies^24^ (online Methods) provided no explanation for their functional consequences (Supplementary Table 10).

### Structural Analysis

Full-length structures for VP24 and VP40 were available, as well as structures for the globular domains of GP, sGP, NP, VP30, and VP35 (Supplementary Table 11). It was not possible to model the oligeromerization domains of VP30 and VP35 nor the structure of L apart from a short 105 residue segment of the 2239 residue protein, which contained a single SDP. 47 SDPs could be mapped onto Ebolavirus protein structures (or structural models where structures were not available, see online Methods). Most SDPs are located on protein surfaces (Supplementary Figure 3) and are therefore potentially involved in interaction with cellular and viral binding partners and/or immune evasion. Based on our combined computational and structural analysis we find evidence for eight SDPs that are very likely to alter protein structure/function, with six affecting protein-protein interfaces and two that with the potential to influence protein integrity and hence affect stability, flexibility and conformations of the protein (Table 1). Five additional SDPs may alter protein structure/function but the evidence supporting them is weaker (Supplementary Tables 12–18). Two of these weaker SDPs were present in NP (A705R, R105K - all SDPs are referred to using Ebola virus residue numbering and show the human pathogenic Ebolavirus amino acid first and the Reston virus amino acid second). A705R is likely to introduce a salt bridge with E694 and R105K will alter hydrogen bonding (Supplementary Table 12). The three other SDPs with weaker evidence were present in the glycan cap in GP (see below). The eight confident SDPs were present in V24, VP30, VP35, and VP40. The VP40 and VP24 SDPs revealed the most changes that may relate to differences in human pathogenicity (see below).

### Multiple SDPs are present in the GP glycan cap

GP is highly glycosylated and mediates Ebolavirus host cell entry. Subunit GP1 binds to the host cell receptor(s). Subunit GP2 is responsible for the fusion of viral and host cell membranes. However, their cellular binding partners remain to be defined^1^,^25^,^26^,^27^. Reverse genetics experiments have suggested that GP contributes to human pathogenicity but is insufficient for virulence on its own^28^. We identified SDPs in both GP1 and GP2 (Supplementary Figure 4 and Supplementary Table 12). Three SDPs (I260L, T269S, S307H) are located in the glycan cap that contacts the host cell membrane (Supplementary Figure 4B,C). These changes (particularly S307H at the top of the glycan cap) alter the electrostatic surface of GP (Supplementary Figure 4D) and may therefore alter GP interactions with cellular proteins, however given the glycosylation of GP, it is unlikely that these residues would physically contact the host cell membrane and none of them are near glycosylation sites. So it is not clear what role they may have. GP binding to the endosomal membrane protein NPC1 is necessary for membrane fusion^25^. However, residues important for NPC1 binding (identified by mutagenesis studies in^25^) were conserved in all analyzed Ebolaviruses and the SDPs were not located close to them (Supplementary Figure 5). Thus differences in NPC1 binding do not account for differences in Ebolavirus human pathogenicity. This finding is in concert with very recent data indicating that NPC1 is essential for Ebolavirus replication as NPC1-deficient mice were insusceptible to Ebolavirus infection^27^.

It was not possible to predict the consequences of SDPs in sGP and ssGP (Fig. S23), as there is a lack of functional information available for these proteins^3^,^4^. A 17 amino acid peptide derived from Ebola virus or Sudan virus GP exerted immunosuppressive effects on human CD4+ T cells and CD8+ T cells while the respective Reston virus peptide did not^29^. We identified one SDP in the peptide, which represents the single amino acid change (I604L) previously observed between Reston virus and Ebola virus^29^, demonstrating that this difference is conserved between Reston viruses and all human pathogenic Ebolaviruses.

### Changes in the VP30 dimer may affect pathogenicity

**Figure 3:**
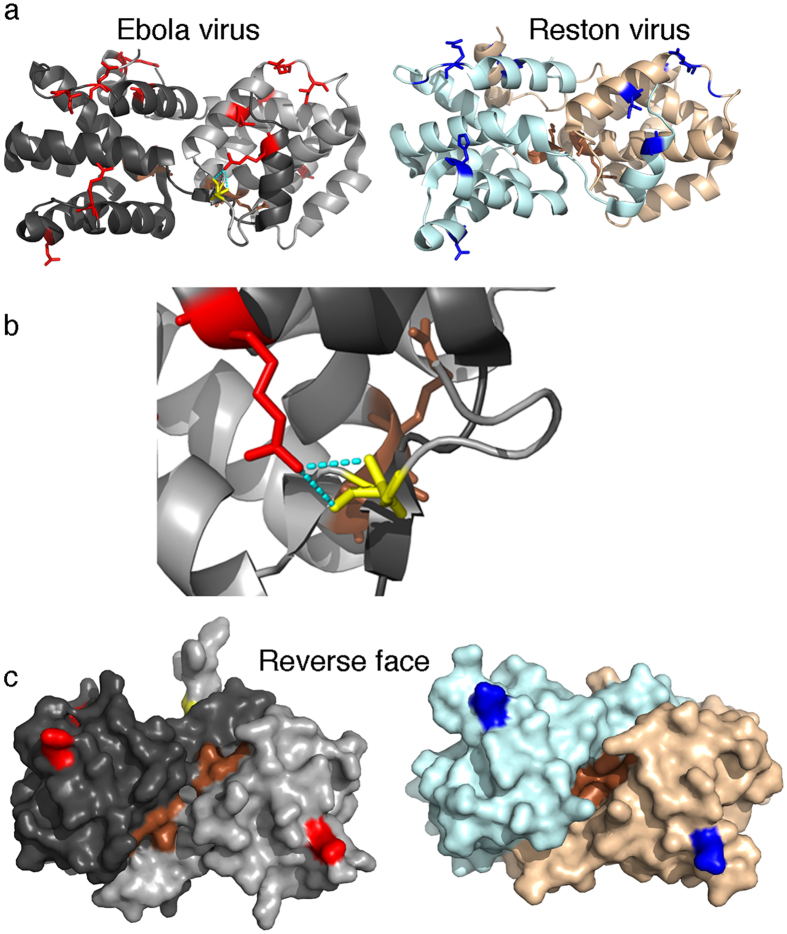
SDPs present in the VP30 dimer. The dimer structure of both Ebola virus (PDB structure 2I8B) and Reston virus (PDB structure 3V7O) VP30 are shown with SDPs indicated (red – Ebola virus, blue – Reston virus) and functional residues (brown – A179, K180). (**a**) Cartoon representation: For the Ebola virus the hydrogen bond of R262 with the residue 141 of the other subunit is shown. (**b**) enlarged display of the hydrogen bond between R262 and the backbone of residue 141. (**c**) Surface representation of the reverse face of the dimer from A, showing the location of the functional residues A179 and K180 within the dimer

Analysis of the VP30 SDPs provided novel mechanistic insights into the structural differences previously observed between Reston virus and Ebola virus VP30^10^ and that may contribute to the differences observed in human pathogenicity between Reston virus and Ebola virus. VP30 is an essential transcriptional co-factor that forms dimers via its C-terminal domain and hexamers via an oligomerization domain (residues 94–112)^30^. The VP30 hexamers activate transcription while the dimers do not, and the balance of hexamers and dimers has been suggested to control the balance between transcription and replication^31^. Crystallization studies have shown that Ebola virus and Reston virus dimers are rotated relative to each other^10^. We observed two SDPs (T150I, R262A) in the dimer interface that can at least partially explain the structural differences between Ebola virus and Reston virus VP30 dimers. Ebola virus R262 is part of the dimer interface and forms a hydrogen bond with the backbone of residue 141 in the other subunit, whereas Reston A262 does not and is not part of the dimer interface (Fig. 3). The removal of the two hydrogen bonds (in the symmetrical dimer) is likely to lead to the different Reston and Ebola virus dimer structures. mCSM predicts this change to be destabilizing with a ΔΔG −0.969 Kcal/mol. The Reston virus conformation also buries functional residues A179 and K180 potentially affecting protein function^10^ (Fig. 2). Moreover, our findings show that the Ebola virus confirmation is conserved in all human-pathogenic Ebolaviruses suggesting that it is relevant for human pathogenicity.

### VP35 SDP present in dimer interface

VP35 is a multifunctional protein that antagonizes interferon signaling by binding double stranded RNA (dsRNA). Structural data are available for both the Ebola virus and Reston virus VP35 monomer and an asymmetric dsRNA bound dimer^9^,^32^,^33^,^34^. These structures are highly conserved, however functional studies have demonstrated that Reston virus VP35 is more stable, has a reduced affinity for dsRNA, and exerts weaker effects on interferon signaling^32^. The increased stability is proposed to be due to a linker between the two subdomains having a short alpha helix in the Reston virus structure^32^. Our analysis shows that the sequence of this linker region is completely conserved in all of the genomes, however an SDP is located close to the linker (A290V). One SDP (E269D) is present in the dimer interface and the shorter aspartate side chain in Reston virus VP35 results in increased distances with the atoms that this aspartate forms hydrogen bonds with: R312, R322, and W324 (Ebola virus numbering; Supplementary Table 13). mCSM predicts this change to be slightly destabilizing to the complex (ΔΔG −0.11 Kcal/mol). This has the potential to alter the stability of the dimer and thus the ability of VP35 to prevent interferon signaling.

It has recently been demonstrated that a VP35 peptide binds NP and modulates NP oligomerization and RNA binding to NP^35^. There are two SDPs (S26T, E48D) in this region. S26T is located on the periphery of the interface. E48D lies outside the solved structure but is within the region required for binding to NP. Both SDPs represent minor changes that maintain the chemical properties of the side chains. Thus, there is no evidence suggesting substantial differences in the binding of this peptide to NP.

### VP40 SDPs may alter oligomeric structure

**Figure 4:**
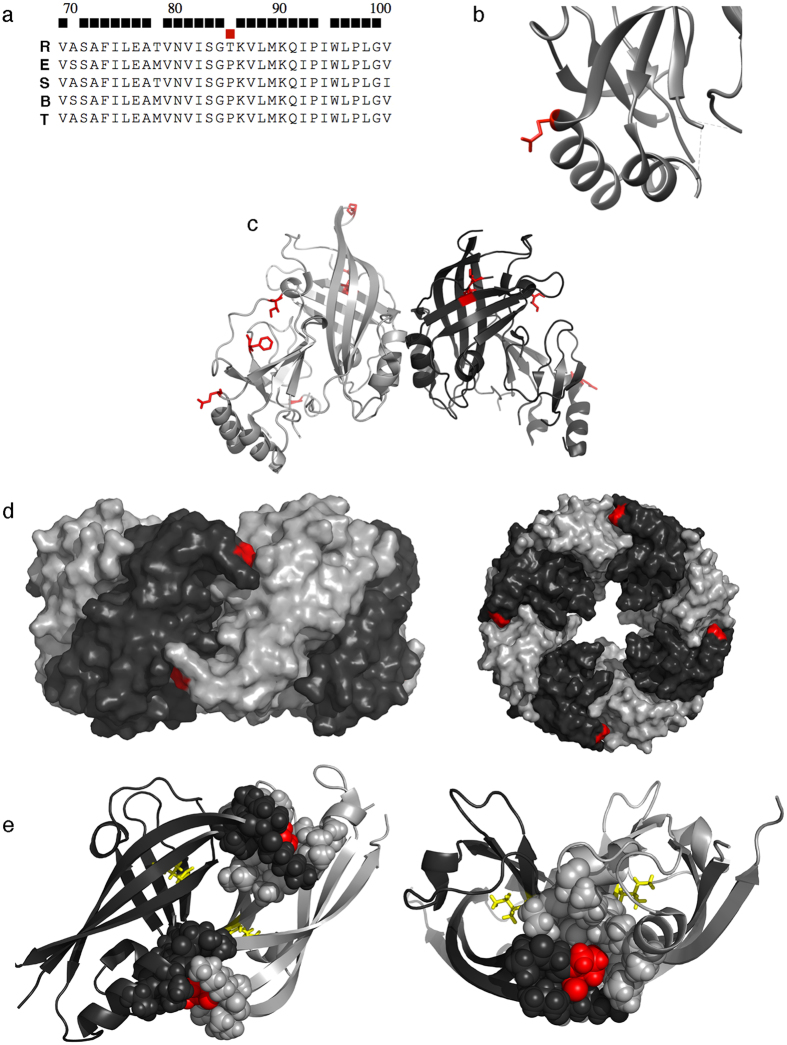
The P85T SDP is present in the VP40 octamer interface. (**a**) Consensus sequence for the region around P85T in *Ebolavirus* species (R, Reston virus; E, Ebola virus; S, Sudan virus; B, Bundibugyo virus; T, Taϊ Forest virus). Black squares indicate positions that are completely conserved in all genomes, red squares SDPs. (**b**) segment of VP40 showing the Q245P SDP (red) from PDB structure 1ES6. (**c**) The VP40 dimer, with SDPs colored red and shown in stick format (PDB structure 4LDB). (**d**) The VP40 octamer, P85 shown in red (side- and top-view) from PDB structure 4LDM. (**E**) Two subunits from the VP40 octamer, P85 is colored red in sphere format, and the SDP I122V is shown as yellow in stick format.

VP40 exists in three known oligomeric forms^36^. Dimeric VP40 is responsible for VP40 trafficking to the cellular membrane. Hexameric VP40 is essential for budding and forms a filamentous matrix structure. Octameric VP40 regulates viral transcription by binding RNA. Two SDPs (P85T and Q245P) can affect VP40 structure. P85T occurs at the VP40 octamer interface site (Fig. 4) in the middle of a run of 14 residues that are completely conserved in all Ebolaviruses (Fig. 4b). In the Ebola virus structure, it is located in an S-G-P-K beta-turn, where the proline at position 85 (P85) confers backbone rigidity. The change to threonine (T) at this residue in Reston viruses introduces backbone flexibility and also provides a side chain with a hydrogen bond donor, potentially affecting octamer structure and/or formation. mCSM predicted this change to have a destabilizing effect (ΔΔG −0.626 Kcal/mol). The Q245P SDP introduces a proline residue into an alpha helix (Fig. 4b), which most likely breaks and shortens helix five, resulting in the destabilization of helices five and six and a change in the hydrophobic core. Interestingly mCSM predicted this change to have little effect on the stability of the protein (predicted ΔΔG 0.059 Kcal/mol). Thus, P85T and Q245P may affect VP40 function and human pathogenicity.

### VP24 SDPs affect KPNA5 binding

**Figure 5:**
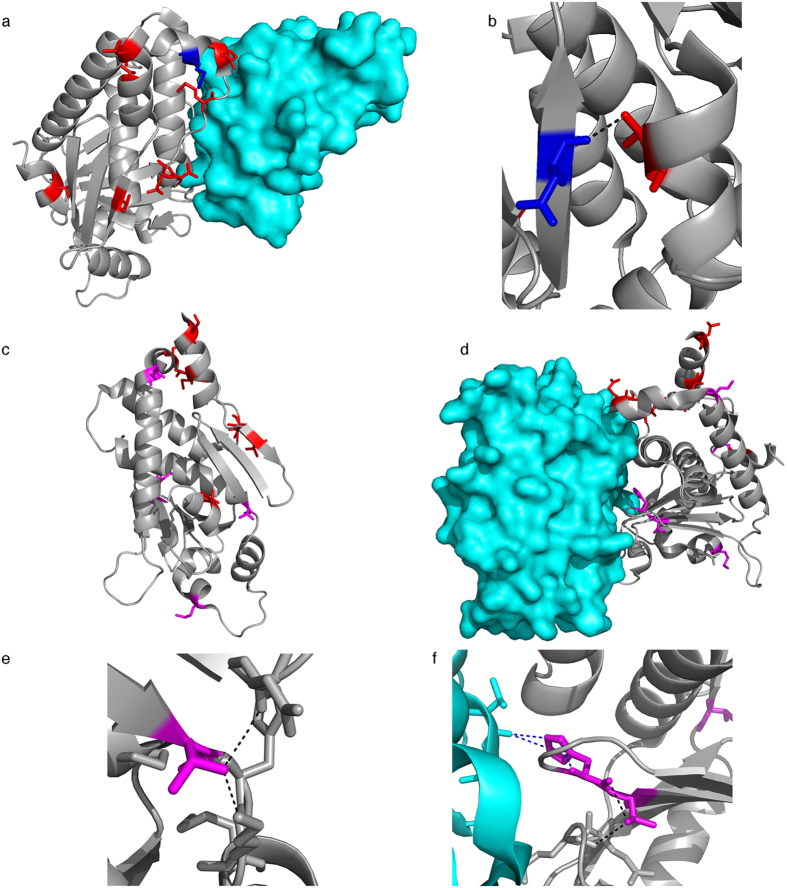
Ebola virus VP24 SDPs and complex with KPNA5. (**a**) VP24 Structure (grey) in complex with KPNA5 (cyan) (PDB structure: 4U2X), with VP24 SDPs (red) and K142 colored blue. (**b**) T226 (red) hydrogen bond with the backbone of D48 (blue). (**c**) VP24 showing residues mutated in rodent adaptation experiments (magenta) and SDPs identified in this study (red). (**d**) Ebola virus VP24 in complex with KPNA5, revierse view shown from A. SDPs are coloured red and residues mutated in adaptation experiments are coloured magenta (**e**) VP24 (grey) and KPNA5 (cyan) complex with residues mutated during adaptation (magenta) and SDPs (red). (**f**) Hydrogen bonds formed by VP24 T50. (**g**) Hydrogen bonds formed by VP24 H186, and T187. Intrachain bonds are colored black and hydrogen bonds between VP24 and KPNA5 are colored blue.

VP24 is involved in the formation of the viral nucleocapsid and the regulation of virus replication^1^,^19^,^37^,^38^,^39^. VP24 also interferes with interferon signaling through binding of the karyopherins α1 (KPNA1), α5, (KPNA5), and α6 (KPNA6) and subsequent inhibition of nuclear accumulation of phosphorylated STAT1 and through direct interaction with STAT1^24^,^40^,^41^,^42^. Eight VP24 SDPs are in regions with available structural information (Supplementary Tables 17 and 18). Seven of these are present on the same face of VP24 (Fig. 5a) suggesting that they affect VP24 interaction with viral and/or host cell binding partners. The SDPs T131S, M136L, and Q139R are present in the KPNA5 binding site (Fig. 5). M136 and Q139 are part of multi-residue mutations in Ebola virus VP24 that removed KPNA5 interactions (Supplementary Table 17)^24^ and are adjacent to K142 (Fig. 5a), mutants of which have shown reduced interferon antagonism^43^. Therefore, M136L and Q139R can exert significant effects on VP24-KPNA5 binding. Additionally, T226A results in the loss of a hydrogen bond between T226 and D48 in Reston virus VP24 (Fig. 5b), with the potential to alter structural integrity and influence protein function. Analysis using mCSM predits the T226A change to be destabilizing with a ΔΔG −0.935 Kcal/mol. mCSM predicted seven of the eight analysed SDPs to be destabilizing (Supplementary Table 2).

VP24-mediated inhibition of interferon signaling may be critical for species-specific pathogenicity^24^,^38^,^40^,^41^,^42^. In this context, VP24 was a critical determinant of pathogenicity in studies in which Ebola viruses were adapted to mice and guinea pigs that are normally insusceptible to Ebola virus disease^5^,^38^,^44^,^45^,^46^. The adaptation-associated VP24 mutations in rodents are located in the KPNA5 binding site with some of them being very close to the VP24 SDPs T131S, M136L, and Q139R that we determined to be in the KPNA5 binding site (Fig. 5c,d, Supplementary Table 19). Additionally some of the mutations are similar to the SDPs in that they would remove hydrogen bonds within VP24 (e.g. T187I, T50I, Fig. 5e,f, & Supplementary Table 19) or alter hydrogen bonding with KPNA5 (H186Y, Fig. 5f & Supplementary Table 19). Thus there is strong evidence suggesting that the VP24 SDPs have a role in rendering the Reston virus non-pathogenic in humans.

## Discussion

In this study, we have combined the computational identification of residues that distinguish Reston viruses from human pathogenic *Ebolavirus* species with protein structural analysis to identify determinants of Ebolavirus pathogenicity. The results from this first comprehensive comparison of all available genomic information on Reston viruses and human pathogenic Ebolaviruses detected SDPs in all proteins but only few of them may be responsible for the lack of Reston virus human pathogenicity.

Our analysis mapped 47 of the 189 SDPs onto protein structure, so additional SDPs may be relevant but the structural data needed to reliably identify them is missing. Although it is difficult to conclude the extent to which each individual SDP contributes to the differences in human pathogenicity between Reston viruses and the other Ebolaviruses, we can identify certain SDPs that have a particularly high likelihood to be involved. SDPs present in the oligomer interfaces of VP30, VP35, and VP40 may affect viral protein function. VP24 SDPs may interfere with VP24-KPNA5 binding and affect viral inhibition of the host cell interferon response. These findings suggest that changes in protein-protein interactions represent a central cause for the variations in human pathogenicity observed in Ebolaviruses. VP24 and VP40 in particular contain multiple SDPs that are likely to contribute to differences in human pathogenicity. Where possible the SDPs have been considered collectively, such as for VP24, where most of the SDPs are present on a single face of the protein (Fig. 5a) and three of them are present in the interface with KPNA5. Beyond this it is difficult to interpret how any combination of SDPs might be responsible for the differences in human pathogenicity.

Our data also demonstrate that relevant changes explaining differences in virulence between closely related viruses can be identified by computational analysis of protein sequence and structure. Such computational studies are particularly important for the investigation of Risk Group 4 pathogens like Ebolaviruses whose investigation is limited by the availability of appropriate containment laboratories.

The role of VP24 appears to be central given the large number of SDPs we identify as likely to affect function, particularly KPNA5 binding. This is also highlighted by the similarity between these SDPs and the mutations that occur in adaptation experiments in mice and guinea pigs^6^,^33^,^39^,^40^,^41^. Consequently, the mutation of a few VP24 SDPs could result in a human pathogenic Reston virus. Given that Reston viruses circulate in domestic pigs, can be spread by asymptomatically infected pigs, and can be transmitted from pigs to humans (possibly by air)^2^,^47^,^48^, there is a concern that (a potentially airborne) human pathogenic Reston viruses may emerge and pose a significant health risk to humans. Notably, asymptomatic Ebolavirus infections have also been described in dogs^2^ and Ebola virus shedding was found in an asymptomatic woman^49^. Thus, there may be further unanticipated routes by which Reston viruses may spread in domestic animals and/or humans enabling them to adapt and cause disease in humans.

In summary our combined computational and structural analysis of a large set of Ebolavirus genomes has identified amino acid changes that are likely to have a crucial role in altering Ebolavirus pathogenicity. In particular the differences in VP24 together with the observation that Ebolavirus adaptation to originally non-susceptible rodents results in rodent pathogenic viruses^6^,^33^,^39^,^40^,^41^ suggest that a few mutations could lead to a human pathogenic Reston virus.

## Materials and Methods

### Ebolavirus nomenclature

The nomenclature in this manuscript follows the recommendations of Kuhn *et al*.^50^. The genus is *Ebolavirus*. It is only italicized if the name refers to the genus but not if it refers to physical viruses or virus parts or constituents such as proteins or genomes. The species are *Zaire ebolavirus* (type virus: Ebola virus, EBOV), *Sudan ebolavirus* (type virus: Sudan virus, SUDV), *Bundibugyo ebolavirus* (type virus: Bundigugyo virus, BDBV), and *Taϊ Forest ebolavirus* (formerly Côte d’Ivoire ebolavirus; type virus: Taϊ Forest virus, TAFV).

### Ebolavirus Genome Sequences

196 complete *Ebolavirus* genomes were downloaded from Virus Pathogen Resource, VIPR (http://www.viprbrc.org/brc/home.spg?decorator=vipr)^18^. The 196 genomes comprise 156 Ebola virus (EBOV), 17 Reston (RESTV), 13 Sudan (SUDV), 7 Bundibugyo (BDBV) and 3 Taï Forest (TAFV) species (Supplementary Table 20). Open Reading Frames (ORFs) in the genomes were identified using EMBOSS^51^. The ORFs were then mapped to the nine Ebolavirus proteins.

### Multiple Sequence Alignments and identification of specificity determining positions

Multiple sequence alignments were generated for each of the Ebolavirus proteins using Clustal Omega^52^, with default settings. Protein sequence identities between the different sequences were obtained form the Clustal Omega output. The effective number of independent sequences present was calculated for the alignment for each protein by building an hmm for the alignment using hmmer^53^. The effective number of independent sequences identified ranged from 88 for the VP24 and L proteins to 148 in NP (Table S21).

The s3det algorithm^12^ was used to predict specificity determining positions (SDPs) using a supervised mode with sequences assigned to predetermined groups/subfamilies with all of the human pathogenic sequences in one group and the Reston virus sequences in a second group. The sensitivity of the SDP analysis to the number of sequences used was considered by subsampling the sequences (see Supplementary Methods and Supplementary Figs S6–S8). SDPs were compared to known functional residues (many from mutagenesis studies) in Ebolavirus proteins catalogued in UniProt^54^ and in the literature.

### Phylogenetic Trees

Bayesian Phylogenetic trees were generated using BEAST v1.8.2^55^, then the consensus tree for each set of 10000 trees was calculated with TreeAnnotator and the node labels obtained analyzing the trees with FigTree [ http://tree.bio.ed.ac.uk/software/figtree/]. TreeAnnotator and BEAUti, are part of the BEAST package.

The Maximum Likelihood Phylogenetic trees were generated using RaxML8^56^. A full Maximum Likelihood analysis and 1000 Bootstrap replicate searches were run in order to obtain the best scoring ML tree for each set of sequences.

Phylogenetic trees were generated using default settings in both BEAST and RaxML8, according to the type of input data. All phylogenetic trees were analyzed and plotted using the R “ape” package^57^.

### Structural Analysis

Where available, protein structures for the Ebolavirus proteins were obtained from the protein databank^58^. Where full length protein structures were not available the proteins were modelled using Phyre2^59^. SDPs were mapped onto the protein structures using PyMOL. Solvent accessibility for SDPs was calculated using DSSP^60^.

The Reston virus structures of GP1 and GP2 were modeled using one-to-one threading in Phyre2^59^ with the EBOV GP trimer structure (PDB code 3CSY) used as a template. A model of a Reston virus GP trimer structure was generated by aligning the modelled Reston virus GP1 and GP2 structures to their corresponding chains in the Ebola virus trimer.

The Coulombic Electrostatic Potential for the proteins was calculated using Delphi, with default parameters^61^. The electrostatics map was visualized and analyzed using Chimera^62^.

mCSM^63^ was used to predict the effect of each individual SDP on the stability of the protein. The Ebola virus structures were used as input and the relevant amino acid changed to the one present in the Reston virus.

## Additional Information

**How to cite this article**: Pappalardo, M. *et al*. Conserved differences in protein sequence determine the human pathogenicity of Ebolaviruses. *Sci. Rep.* **6**, 23743; doi: 10.1038/srep23743 (2016).

## Supplementary Material

Supplementary Information

## Figures and Tables

**Table 1:** SDPs that are likely to alter Reston virus protein structure and function.

**Protein**	**SDP**	**Interface**	**Protein Integrity**
VP24	T131S	KPNA5 interface	
VP24	M136L	KPNA5 interface	
VP24	Q139R	KPNA5 interface	
VP24	T226A		Loss of Hydrogen bond
VP40	P85T	Octamer interface	
VP40	Q245P		Breaks α helix
VP30	R262A	Dimer interface – loss of Hydrogen bond	
VP35	E269D	Dimer interface	
