# Supplementary material for: Conserved differences in protein sequence determine the human pathogenicity of Ebolaviruses
Source: Sci Rep. 2016 Mar 24;6:23743. doi: 10.1038/srep23743 (PMC4806318; doi:10.1038/srep23743)
Supplement: Supplementary Information [file srep23743-s1.pdf]

Supplementary Materials

Conserved differences in protein sequence determine the human pathogenicity of Ebolaviruses

Authors: Morena Pappalardo†, Miguel Juliá†, Mark J. Howard, Jeremy S. Rossman*, Martin Michaelis*, Mark N. Wass*

Affiliation:

Centre for Molecular Processing and School of Biosciences, University of Kent, Canterbury, Kent CT2 7NJ, UK.

*Correspondence to: M.N.Wass@kent.ac.uk (Mark N. Wass), M.Michaelis@kent.ac.uk (Martin Michaelis), J.S.Rossman@kent.ac.uk (Jeremy S. Rossman)

† equal contribution.

Supplementary Material

Supplementary Methods - Subsampling of sequence data

The sensitivity of the SDP analysis to the number of sequences available was considered by subsampling the sequences. Sampling was performed for; only the human pathogenic group; only the Reston group; and for both groups simultaneously. Subsampling was performed using between 10%-90% of sequences in the group, increasing in 10% increments. For each percentage setting the group was sampled 50 times. Where both groups were sampled simultaneously they were done so with the same percentage of sequences i.e. at 20% sampling the SDPs were predicted each time using 20% of the human pathogenic sequences in one group and 20% of the Reston sequences in the other. For each sample s3det was run to predict SDPs using the same settings as for the full dataset. Completely conserved SDPs are also compared to those that are not completely conserved. the The total number of SDPs predicted when sampled is shown in supplementary Figure 6. When the sequences of human pathogenic Ebolaviruses were sampled, while the number of Reston sequences remained constant, we observed that the number of SDPs predicted decreased as the proportion of sequences sampled increased. We further observed that even when a very high proportion of sequences was sampled (70%-90%), that there was still some variation in the number of SDPs, indicating that there was still further information present in the excluded sequences. When the Reston virus sequences were sampled, the pattern observed varied between the proteins (Supplementary Figure 6B). For GP, L and VP30, sampling resulted in more SDPs being predicted than in the full dataset, with the number reducing as the proportion of sequences sampled increased. For NP, sampling the Reston sequences generated some samples where fewer SDPs than the total present in the full dataset were predicted and other samples where a larger number of SDPs were predicted. This is possible for SDPs that are not completely conserved in the two groups, as sampling may generate some sets of sequences where these positions appear variable and others where they are conserved. For VP35, sampling led to fewer SDPs being predicted until 90% of sequences were used. The number of SDPs in VP24 and VP40 was invariant across all samples. When sampling both groups (Supplementary Figure 6C) we found that the number of SDPs predicted very quickly converged to the number of SDPs present in the full dataset.

We then considered the number of SDPs predicted that are present in the full dataset and those that are present only in sampling (Supplementary Figure 7). When the human pathogenic sequences were sampled (Supplementary Figure 7A), we found that the vast majority of SDPs in the full data set were predicted at all sampling levels. We also found that when a small proportion of sequences were sampled,

that many new SDPs were predicted, which for some proteins (e.g. GP, NP and VP40) may be greater than the total number of SDPs present in the full dataset. This may not be too surprising given that positions that are variable in the full dataset may appear to be conserved when a small sample of sequences was taken. As the proportion of sequences sampled increased, very few new SDPs were predicted. Sampling the Reston sequences (Supplementary Figure 7B) we again found that the vast majority of SDPs present in the full dataset was present in all samples. The number of new SDPs present in samples was much smaller than for sampling of the human pathogenic sequences, which is likely to be due to the smaller number of Reston sequences, resulting in fewer samples where positions are conserved that are not conserved in the full data set. When both groups were sampled, results were very similar to that observed when the human pathogenic group was sampled (Supplementary Figure 7C).

Finally, we considered the number of SDPs in the sampling sets that are completely conserved and those that are not (Supplementary Figure 8). In conjunction with the data from Supplementary Figure 7, this shows that sampling generates new SDPs that are completely conserved (i.e. only one amino acid in each group) and also some where there is variation within one or both groups. As the proportion of sequences sampled increased these numbers quickly converged to the numbers observed in the full dataset. Some of these included SDPs which in some samples were completely conserved but as further sequences were added, variation was introduced and they were no longer completely conserved. In such cases there was a change ranking for the SDP, as when completely conserved it was ranked 1, and this ranking was reduced once the position was not completely conserved.

Supplementary Figures

Supplementary Figure 1. Phylogenetic tree of the Ebolavirus genomes and individual proteins**.** Bayesian and Maximum Likelihood phylogenetic trees are shown for the Ebolavirus genomes and each of the Ebolavirus proteins. A) genome Bayesian tree. B) Genome maximum likelihood tree, C) Bayesian tree for protein L, D)Maximum likelihood tree for protein L, E)Bayesian tree for protein GP, F)Maximum likelihood tree for protein GP, G)Bayesian tree for protein NP, H)Maximum likelihood tree for protein NP, I)Bayesian tree for protein VP24, J)Maximum likelihood tree for protein VP24, K)Bayesian tree for protein VP30, L)Maximum likelihood tree for protein VP30, M)Bayesian tree for protein VP35, N)Maximum likelihood tree for protein VP35, O)Bayesian tree for protein VP40. P)Maximum likelihood tree for protein VP40. All trees use Ebola virus as root (EBOV, Ebola virus; BDBV, Bundibugyo virus; SUDV, Sudan virus; TAFV, Taï Forest virus; RESTV, Reston virus).

Fig S1A

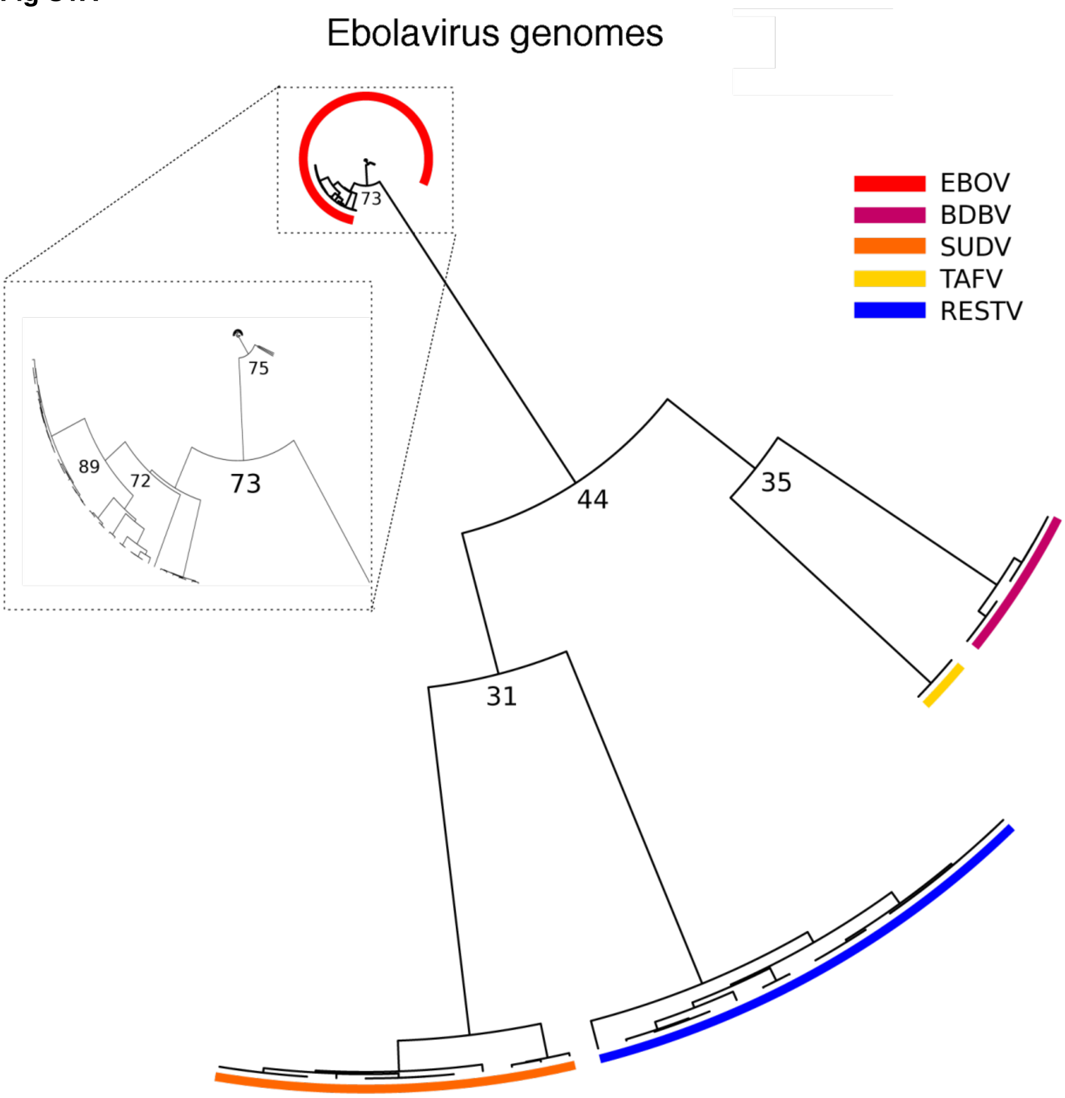

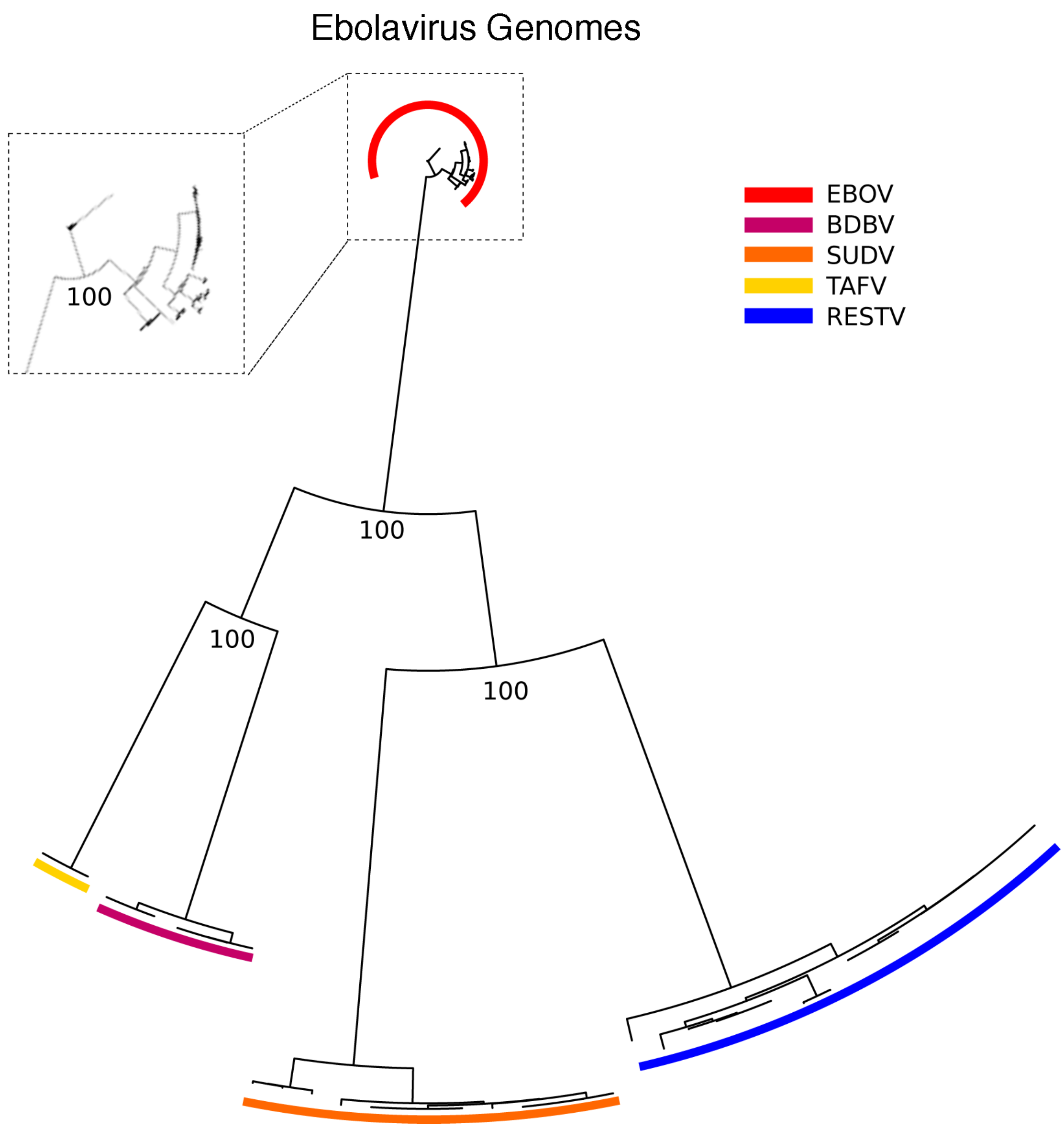
Ebolavirus Genomes
100
100
100
100
EBOV
BDBV
SUDV
TAFV
RESTV

Fig S1B.

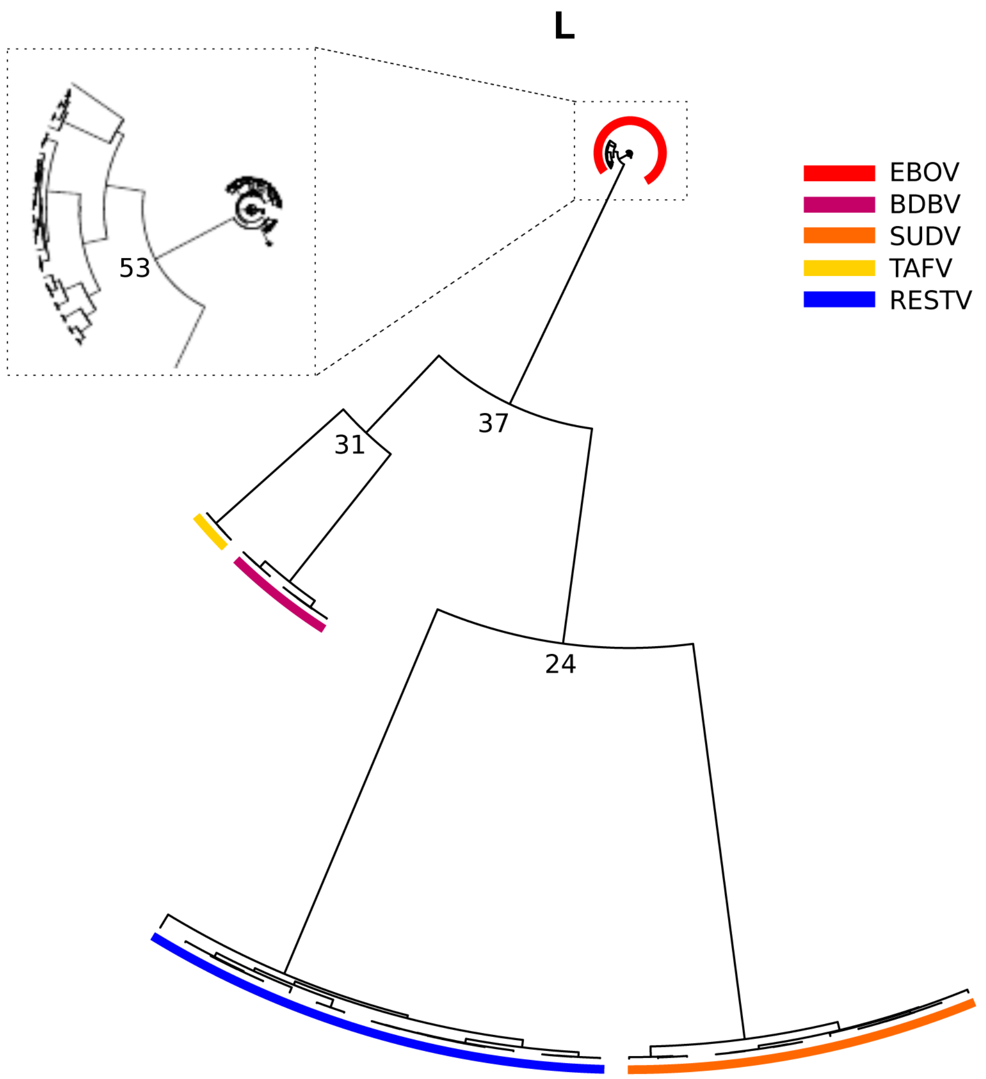

Fig S1C.

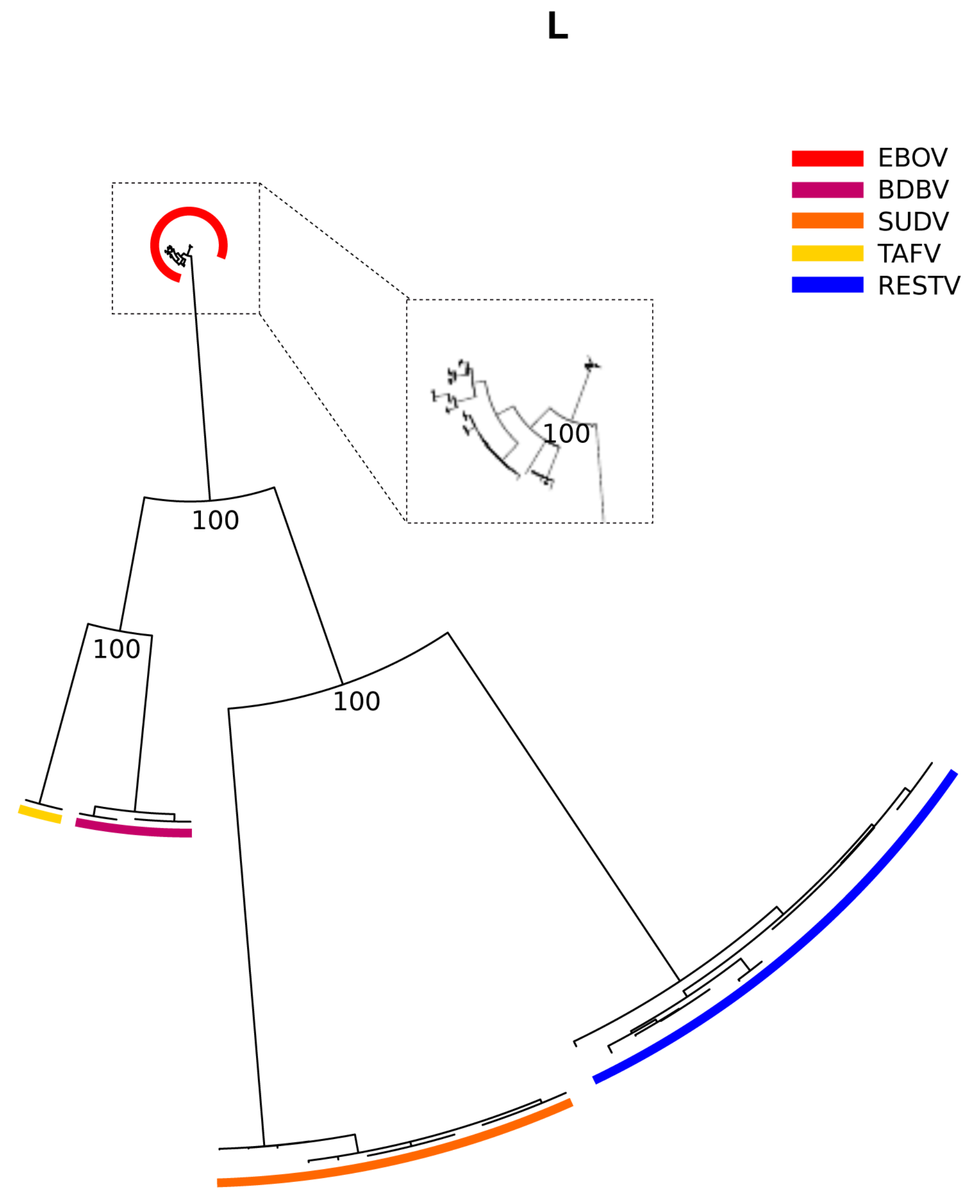

Fig S1D.

GP

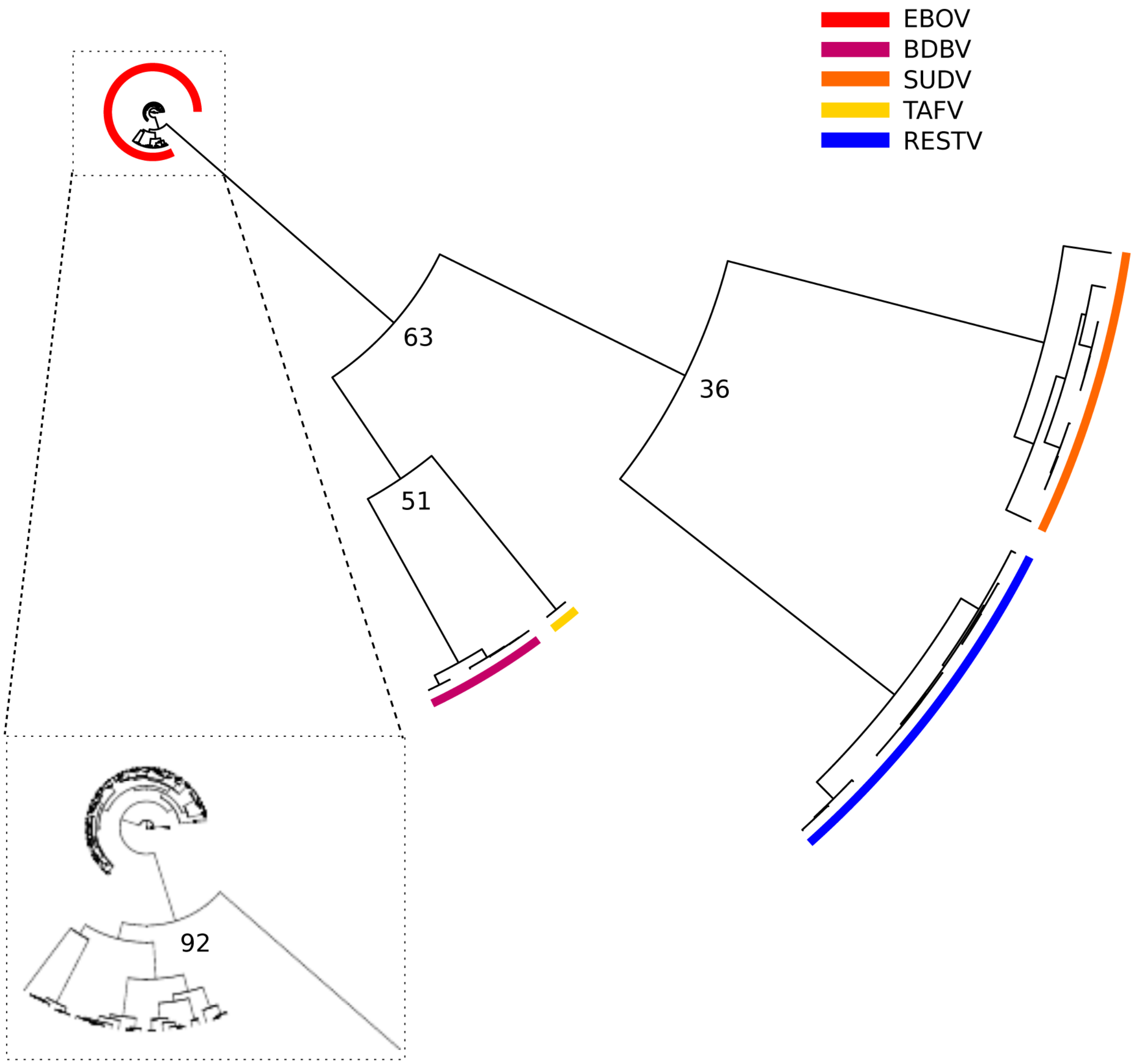

Fig S1E .

GP

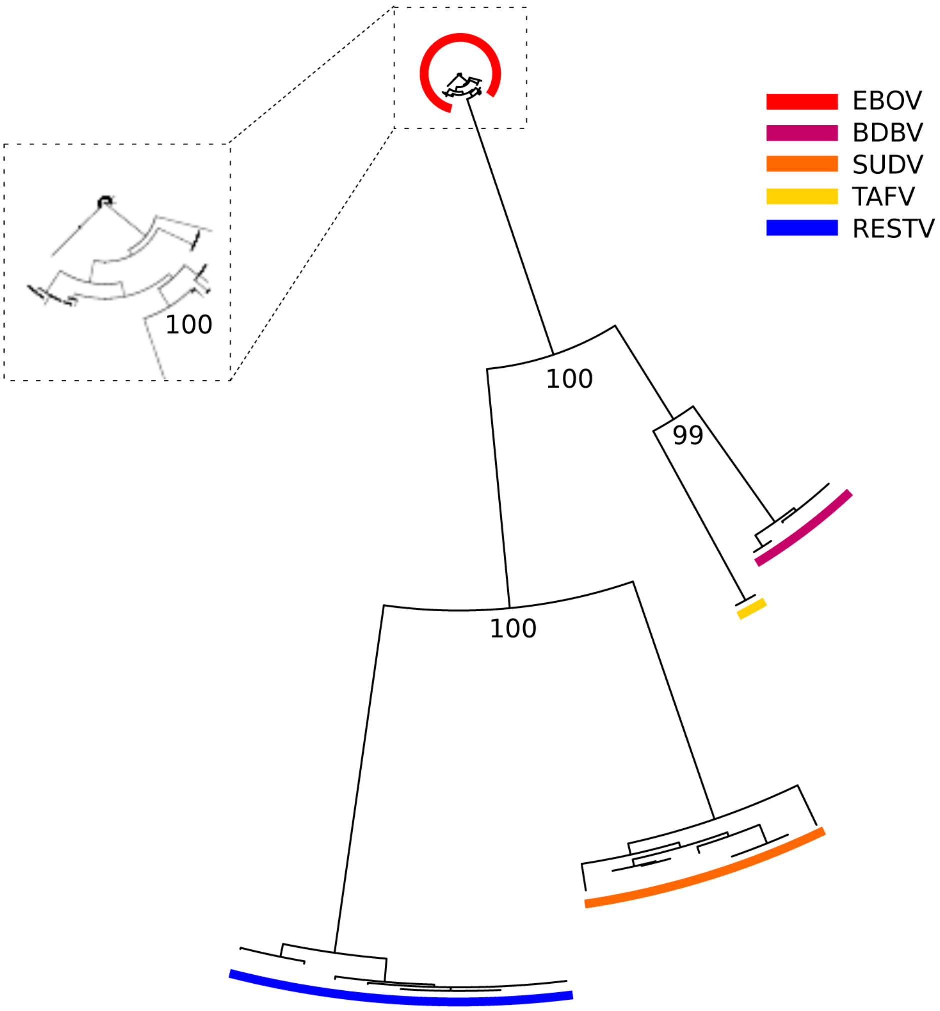

Fig S1F.

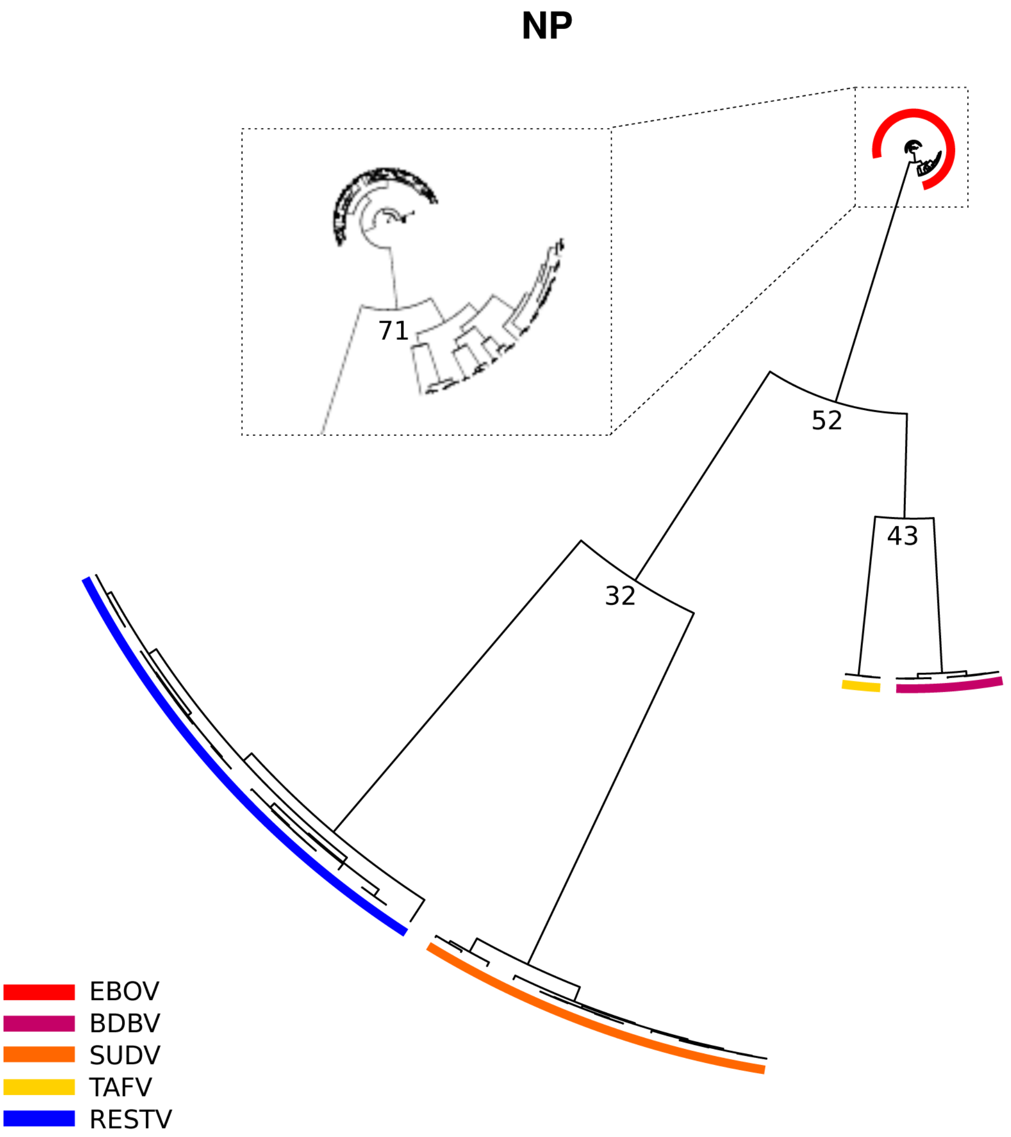

Fig S1G.

NP

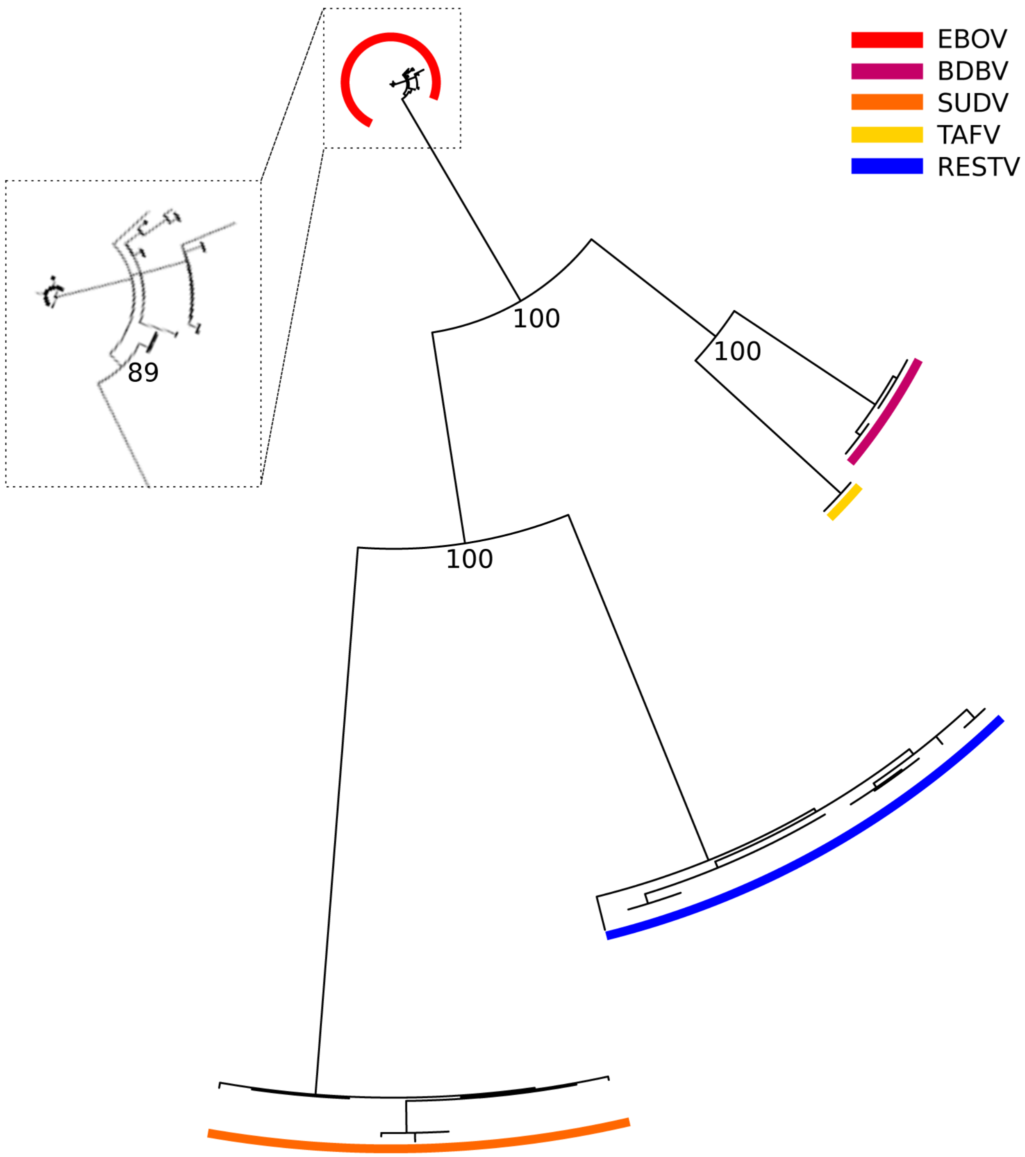

Fig S1H

VP24

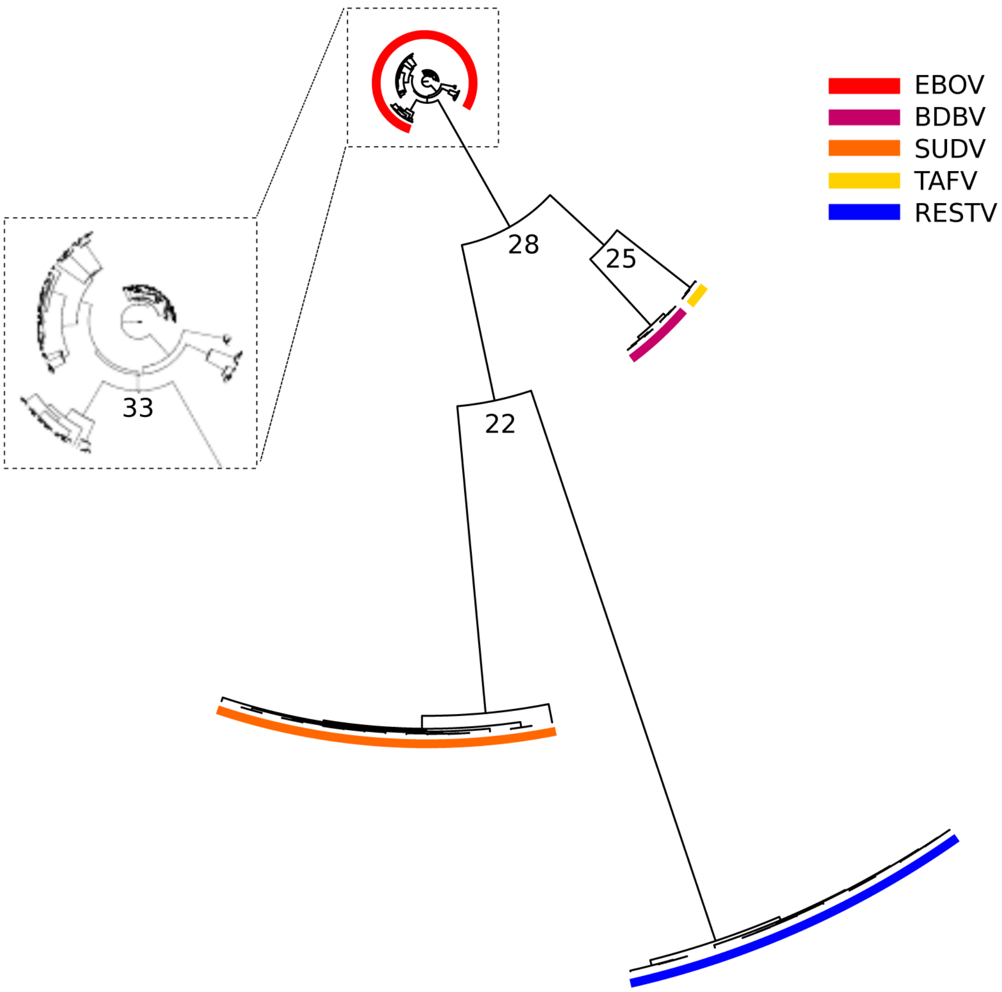

Fig S1I.

VP24

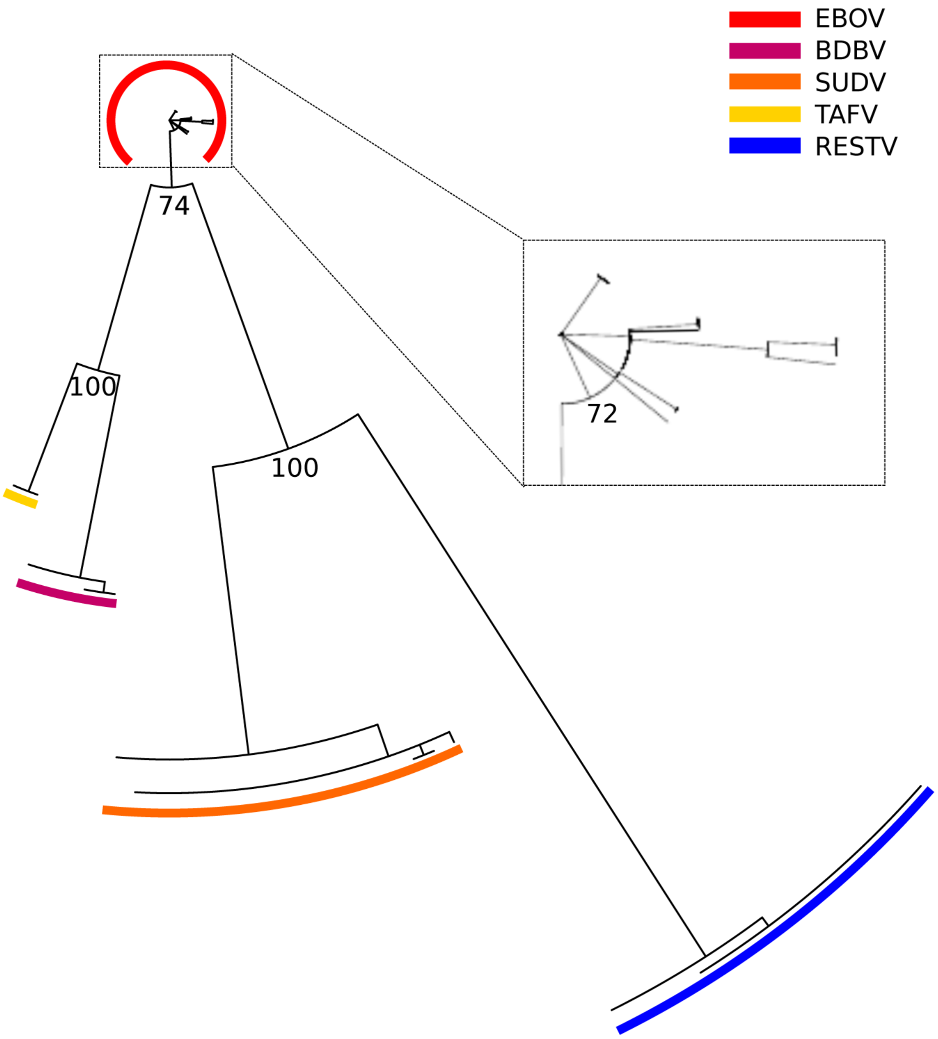

Fig S1J.

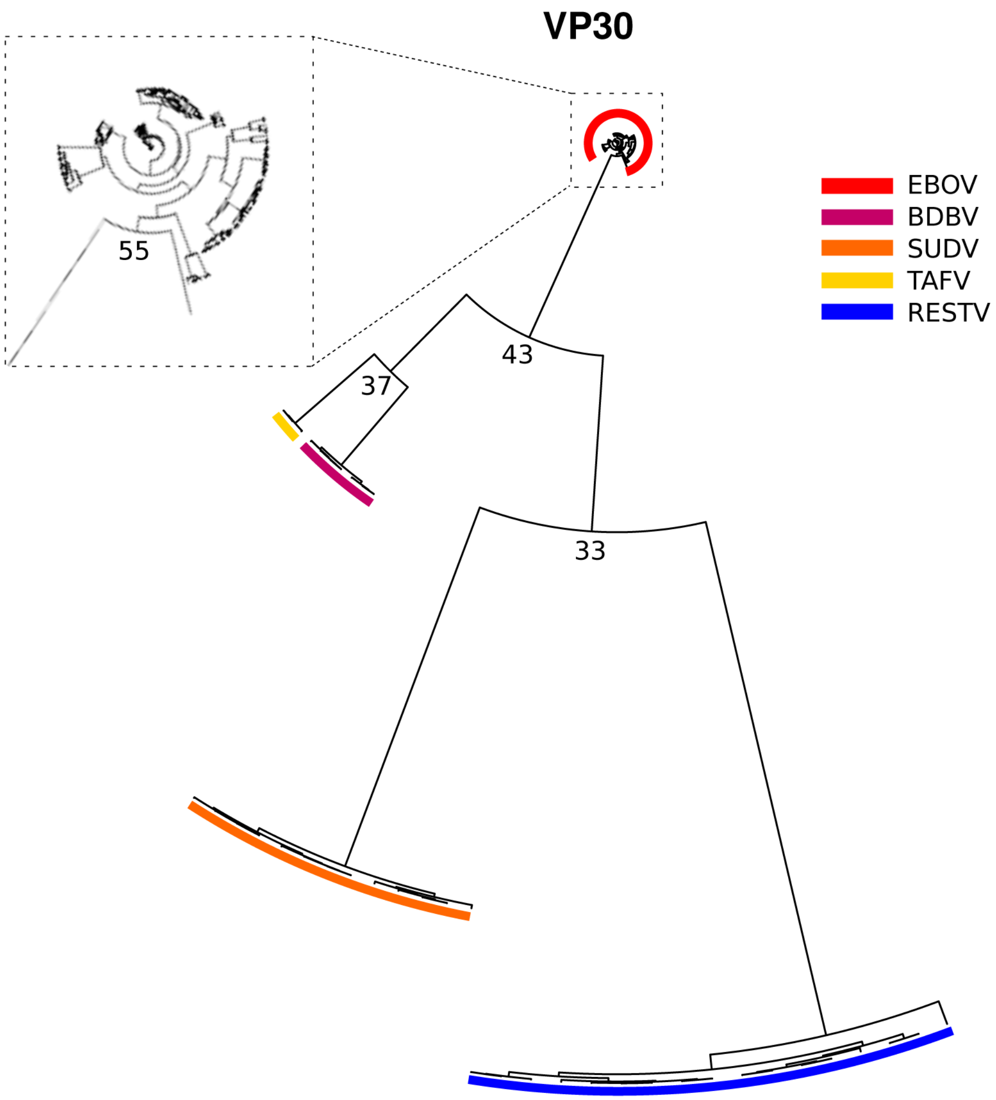

Fig S1K.

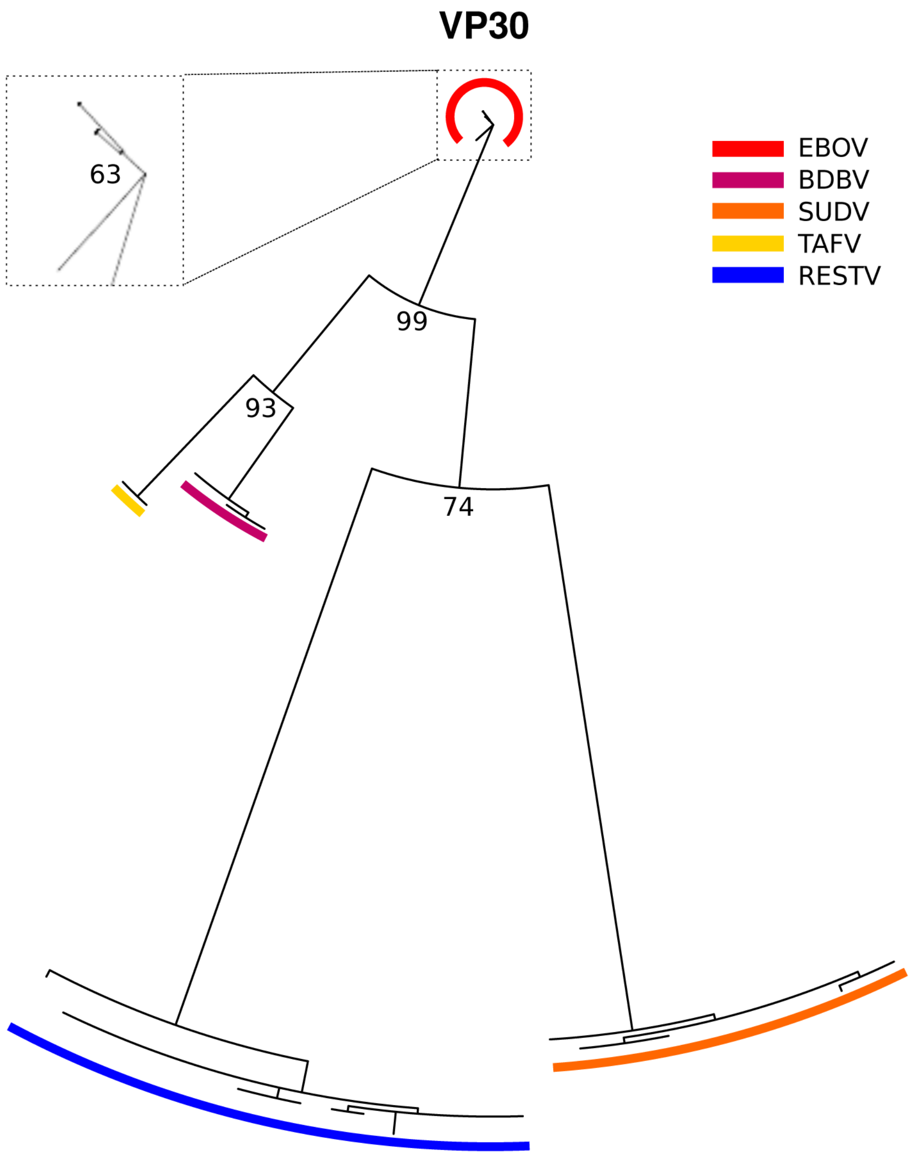

Fig S1L

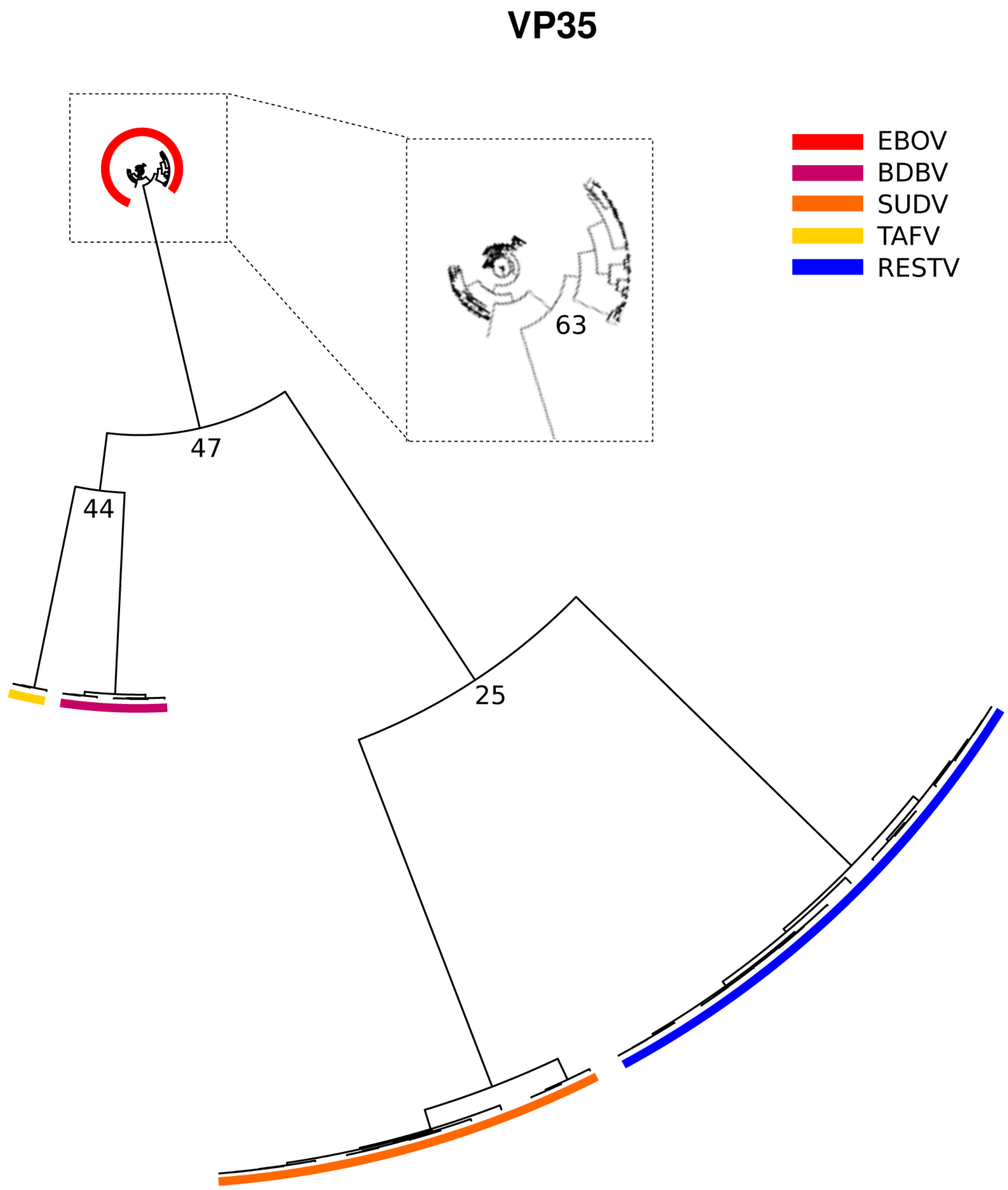

Fig S1M.

VP35

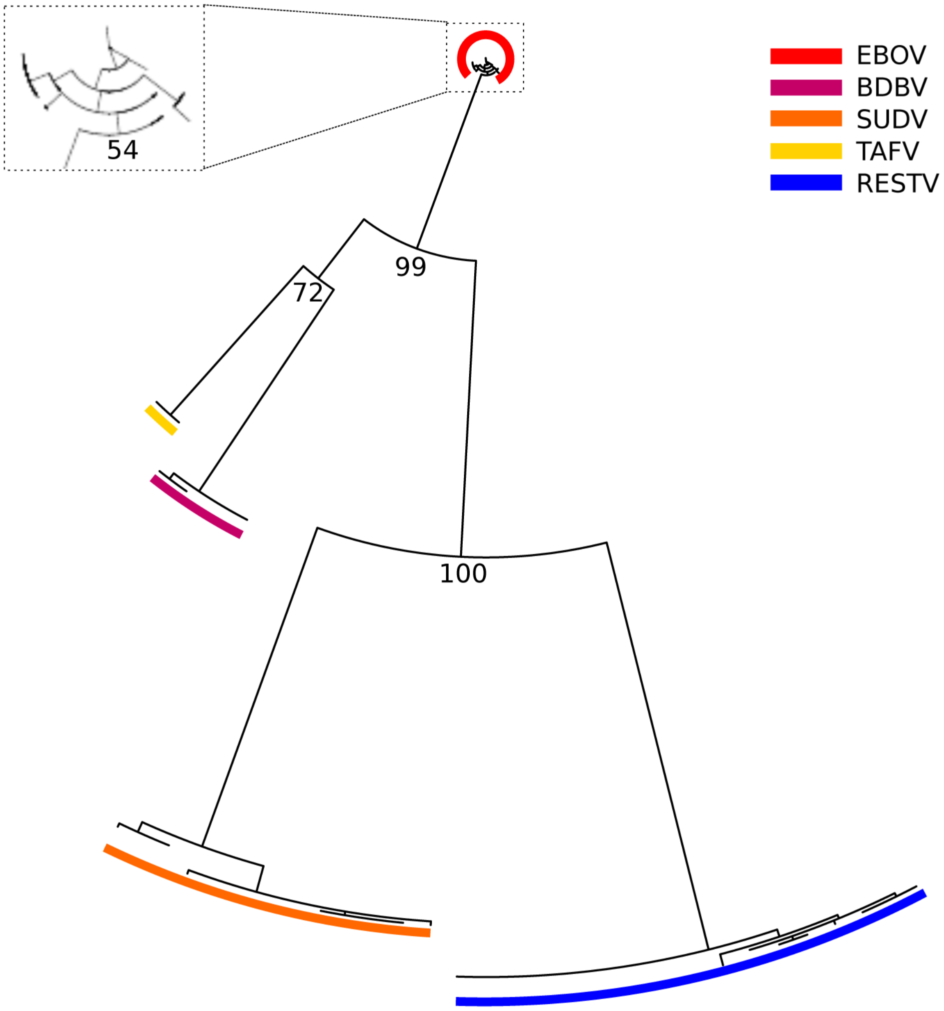

Fig S1N.

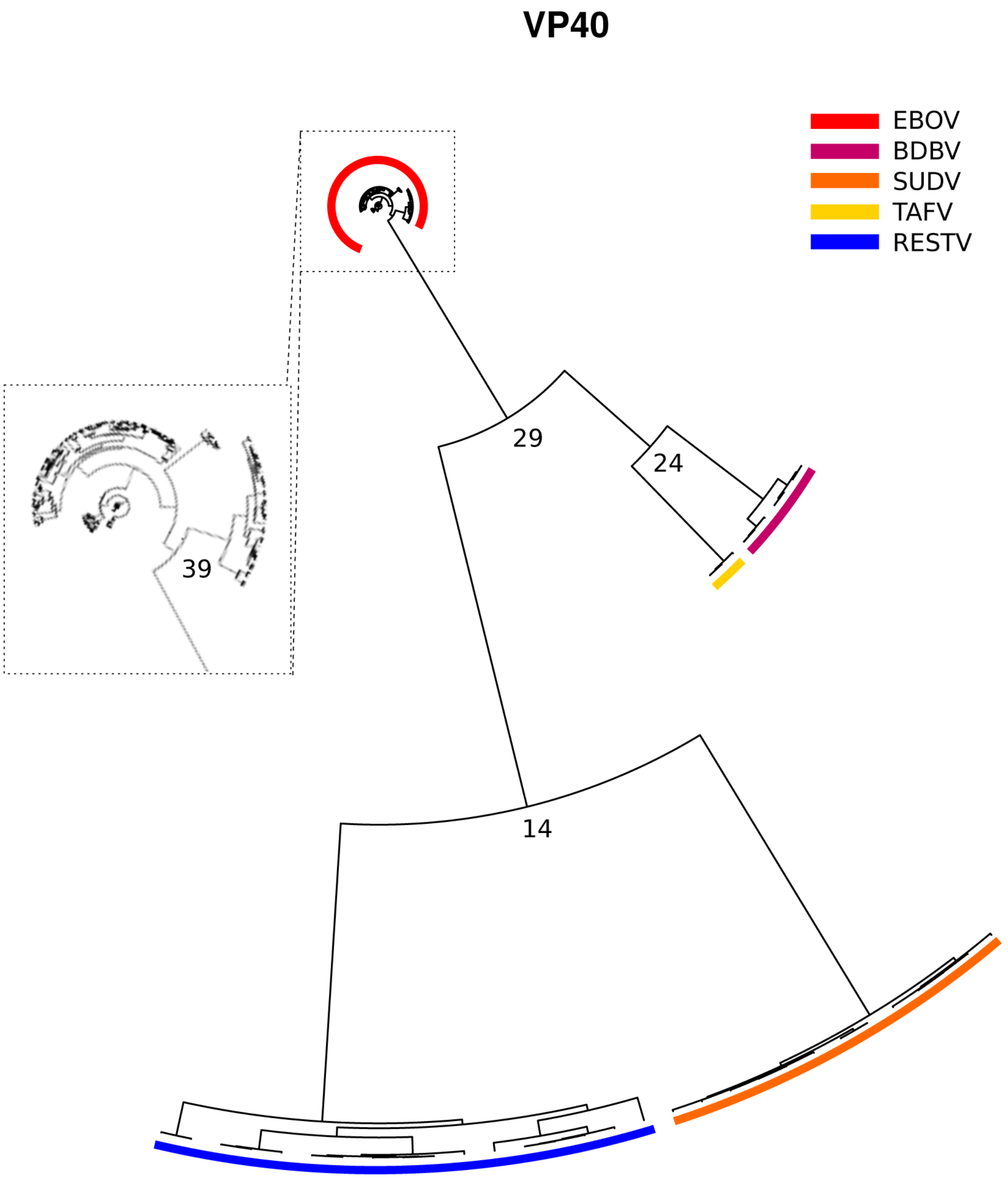

Fig S1O.

VP40

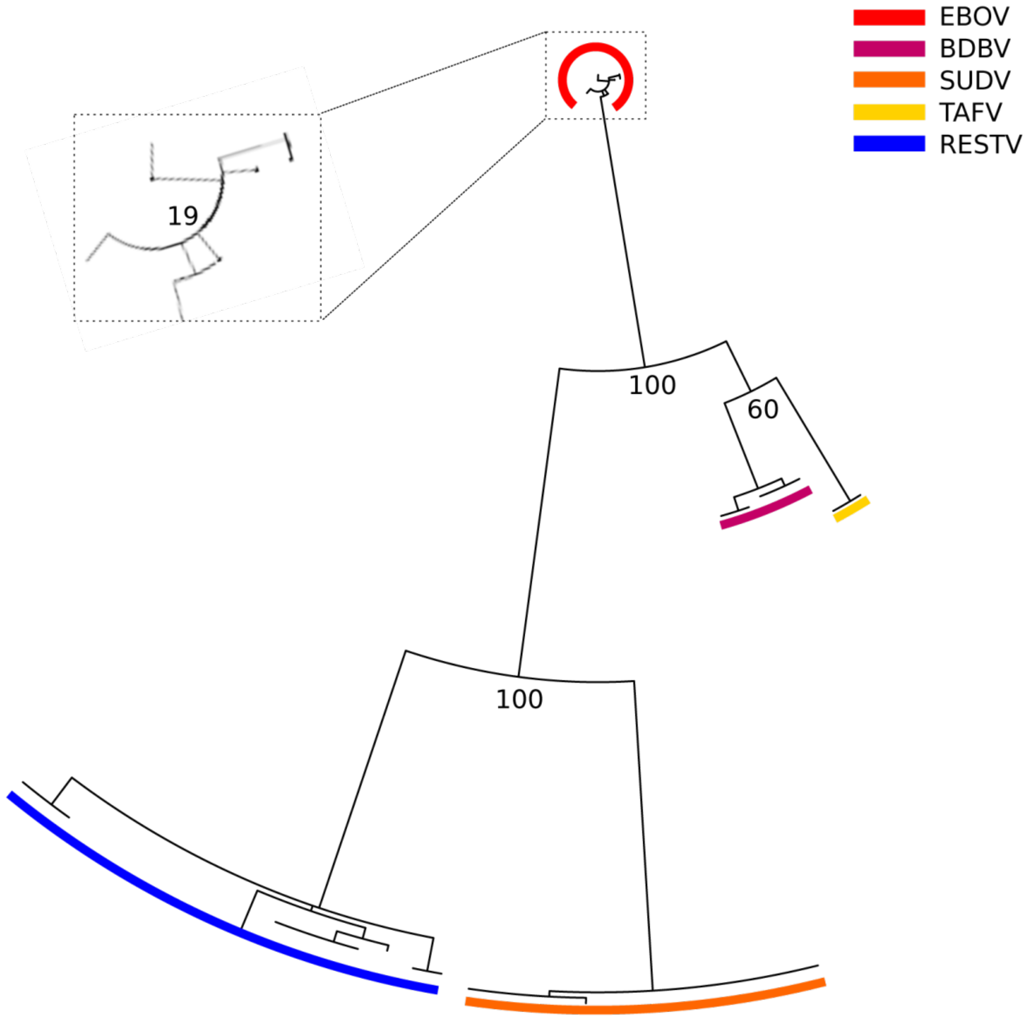

Fig S1P.

Supplementary Figure 2. Ebolavirus protein consensus sequences and SDPs. The consensus sequence for each *Ebolavirus* species is shown for each Ebolavirus protein. The row above the alignment indicates positions that are 100% conserved across all Ebolavirus sequences (black) or specificity determining positions (SDPs) that discriminate Reston viruses from the four human pathogenic *Ebolavirus* species (red); R, Reston virus; E, Ebola virus; S, Sudan virus; B, Bundibugyo virus; T, Taï Forest virus. A) for VP24, B) for GP, C) for VP40, D) VP35, E)VP30, F) sGP, G) NP, H)L.

A – VP24

```
  1        10        20        30        40        50        60
R MAKATGRYNLVPPKKDMEKGVIFSDLCNFLITQTLQGWKVYWAGIEFDVSQKGMALLTRL
E MAKATGRYNLISPKKDLEKGVVLSDLCNFLVSQTIQGWKVYWAGIEFDVTHKGMALLHRL
S MAKATGRYNLVTPKRELEQGVVFSDLCNFLVTPTVQGWKVYWAGLEFDVNQKGITLLNRL
B MAKATGRYNLVSPKKDLERGLVLNDLCTFLVDQTIQGWRVTWVGIEFDIAQKGMALLHRL
T MAKATGRYNLISPKKDLEKGLVLNDLCTLSVAQTVQGWKVTWAGIEFDVTQKGMALLHRL

           70        80        90       100       110       120
R KTNDFAPAWAMTRNLFPHLFQNPNSVIQSPIWALRVILAAGLQDQLLDHSLVEPLTGALG
E KTNDFAPAWSMTRNLFPHLFQNPNSTIESPLWALRVILAAGIQDQLIDQSLIEPLAGALG
S KVNDFAPAWAMTRNLFPHLFKNQQSEVQTPIWALRVILAAGILDQLMDHSLIEPLSGALN
B KTADFAPAWSMTRNLFPHLFQNSNSTIESPLWALRVILAAGIQDQLIDQSLVEPLAGALS
T KTSDFAPAWSMTRNLFPHLFQNPNSTIESPLWALRVILAAGIQDQLIDQSLIEPLAGALG

          130       140       150       160       170       180
R LISDWLLTTTSTHFNLRTRSVKDQLSLRMLSLIRSNILQFINKLDALHVVNYNGLLSSIE
E LISDWLLTTNTNHFNMRTQRVKEQLSLKMLSLIRSNILKFINKLDALHVVNYNGLLSSIE
S LIADWLLTTSTNHFNMRTQRVKDQLSMRMLSLIRSNIINFINKLETLHVVNYKGLLSSVE
B LVSDWLLTTNTNHFQMRTQHAKEQLSLKMLSLVRSNILKFISQLDALHVVNYNGLLSSIE
T LIADWLLTTGTNHFQMRTQQAKEQLSLKMLSLVRSNILKFINQLDALHVVNYNGLLSSIE

          190       200       210       220       230       240
R IGTSTHTIIITRTNMGFLVEVQEPDKSAMNSKRPGPVKFSLLHESAFKPFTRVPQSGMQS
E IGTQNHTIIITRTNMGFLVELQEPDKSAMNRKKPGPAKFSLLHESTLKAFTQGSSTRMQS
S IGTPSYAIIITRTNMGYLVEVQEPDKSAMDIRHPGPVKFSLLHESTLKPVATPKPSSITS
B IGTRNHTIIITRTNMGFLVELQEPDKSAMNQKKPGPVKFSLLHESTFKALIKKPATKMQA
T IGTKSHTIIITRTNMGFLVELQEPDKSAMNTRKPGPVKFSLLHESTLKTLAKKPATQMQA

          250
R LIMEFNSLLAI
E LILEFNSSLAI
S LIMEFNSSLAI
B LILEFNSSLAI
T LILEFNSSLAI
```

B - GP

```
  1        10        20        30        40        50        60
R MGSGYQLLQLPRERFRKTSFLVWVIILFQRAISMPLGIVTNSTLKATEIDQLVCRDKLSS
E -MGVTGILQLPRDRFKRTSFFLWVIILFQRTFSIPLGVIHNSTLQVSDVDKLVCRDKLSS
S -MGGLSLLQLPRDKFRKSSFFVWVIILFQKAFSMPLGVVTNSTLEVTEIDQLVCKDHLAS
B -MVTSGILQLPRERFRKTSFFVWVIILFHKVFPIPLGVVHNNTLQVSDIDKLVCRDKLSS
T -MGASGILQLPRERFRKTSFFVWVIILFHKVFSIPLGVVHNNTLQVSDIDKFVCRDKLSS

           70        80        90       100       110       120
R TSQLKSVGLNLEGNGIATDVPSATKRWGFRSGVPPKVVSYEAGEWAENCYNLEIKKSDGS
E TNQLRSVGLNLEGNGVATDVPSVTKRWGFRSGVPPKVVNYEAGEWAENCYNLEIKKPDGS
S TDQLKSVGLNLEGSGVSTDIPSATKRWGFRSGVPPKVVSYEAGEWAENCYNLEIKKPDGS
B TSQLKSVGLNLEGNGVATDVPTATKRWGFRAGVPPKVVNYEAGEWAENCYNLDIKKADGS
T TSQLKSVGLNLEGNGVATDVPTATKRWGFRAGVPPKVVNCEAGEWAENCYNLAIKKVDGS

          130       140       150       160       170       180
R ECLPLPPDGVRGFPRCRYVHKVQGTGPCPGDLAFHKNGAFFLYDRLASTVIYRGTTFAEG
E ECLPAAPDGIRGFPRCRYVHKVSGTGPCAGDFAFHKEGAFFLYDRLASTVIYRGTTFAEG
S ECLPPPPDGVRGFPRCRYVHKAQGTGPCPGDYAFHKDGAFFLYDRLASTVIYRGVNFAEG
B ECLPEAPEGVRGFPRCRYVHKVSGTGPCPEGFAFHKEGAFFLYDRLASTIIYRSTTFSEG
T ECLPEAPEGVRDFPRCRYVHKVSGTGPCPGGLAFHKEGAFFLYDRLASTIIYRGTTFAEG

          190       200       210       220       230       240
R VVAFLILSEPKKHFWKATPAHEPVNTTDDSTSYYMTLTLSYEMSNFGGEESNTLFKVDNH
E VVAFLILPQAKKDFFSSHPLREPVNATEDPSSGYYSTTIRYQATGFGTNETEYLFEVDNL
S VIAFLILAKPKETFLQSPPIREAVNYTENTSSYYATSYLEYEIENFGAQHSTTLFKIDNN
B VVAFLILPKTKKDFFQSPPLHEPANMTTDPSSYYHTVTLNYVADNFGTNMTNFLFQVDHL
T VIAFLILPKARKDFFQSPPLHEPANMTTDPSSYYHTTTINYVVDNFGTNTTEFLFQVDHL

          250       260       270       280       290       300
R TYVQLDRPHTPQFLVQLNETLRRNNRLSNSTGRLTWTLDPKIEPDVGEWAFWETKKNFSQ
E TYVQLESRFTPQFLLQLNETIYASGKRSNTTGKLIWKVNPEIDTTIGEWAFWETKKNLTR
S TFVRLDRPHTPQFLFQLNDTIHLHQQLSNTTGRLIWTLDANINADIGEWAFWENKKNLSE
B TYVQLEPRFTPQFLVQLNETIYTNGRRSNTTGTLIWKVNPTVDTGVGEWAFWENKKNFTK
T TYVQLEARFTPQFLVLLNETIYSDNRRSNTTGKLIWKINPTVDTSMGEWAFWENKKNFTK

          310       320       330       340       350       360
R QLHGENLHFQILSTHTNNSSDQSPAGTVQGKISYHPPTNNSELVPTDSPPVVSVLTAGRT
E KIRSEELSFTAVSNGPKNISGQSPARTSSDPETNTTNEDHKIMASENSSAMVQVHSQGRK
S QLRGEELSFEALSLNETEDDDAASSRITKGRISDRATRKYSDLVPKNSPGMVPLHIPEGE
B TLSSEELSVILVPRAQDPGSNQKTKVTPTSFANNQTSKNHEDLVPKDPASVVQVRDLQRE
T TLSSEELSFVPVPETQNQVLDTTATVSPPISAHNHAAEDHKELVSEDSTPVVQMQNIKGK

          370       380       390       400       410       420
R EEMSTQGLTNGETI---TGFTANPMTTTIAPSPTMTSEVDNNVPSEQP----NNTASIED
E AAVSHLTTLATISTSPQPPTTKTGPDNSTHNTPVYKLDISEATQVGQHHRRADNDSTASD
S TTLPSQNSTEGRRV---SVNTQETITETAA---TIIGTNGNHMQISTIGIRPSSSQIPSS
B NTVPTSPLNTVPTT-L-IPDTMEEQTTSHYELPNISGNHQERNNTAHPET-------LAN
T DTMPTTVTGVPTTT-P-SPFPINARNTDHTKSFIGLEGPQEDHSTTQPAK-------TTS
```

```
         430       440       450       460       470       480
R S-----PPSASNETIDHSEMNSIQGSNNSAQSPQTKTTPAPTASP--------MTQDPQE
E T-----PPATTA-AGPLKAENTNTSKSAD------SLDLATTTSPQNYS--------ETA
S SPTTAPSPEAQTPTTHTSGPSVMATEE-PTTPPG-SSPGPTTEAP--------TLTTPEN
B N-----PPDNTTPSTPPQ----DGERTSSHTTPSPRPVPTSTIHPTTRETQIPTTMITSH
T Q-----PTNSTESTTLNP----TSEPSSRGTGPSSPTVPNTTESHAELGKTTPTTLPEQH

         490       500       510       520       530       540
R TANSSKPGTSPGSAAEPSQPGLTINTVSKVADSLSPTRKQKRSVRQNTANKCNPDLHYWT
E GNNNTHHQDTGEESASSGKLGLITNTIAGVAGLITGGRRTRREVIVNAQPKCNPNLHYWT
S IT----TAVKTVLPQESTSNGLITSTVTGILGSLGLRKRSRRQTNTKATGKCNPNLHYWT
B DT--DSNRPNPIDISESTEPGLLTNTIRGVANLLTGSRRTRREITLRTQAKCNPNLHYWT
T TA--ASAIPRAVHPDELSGPGFLTNTIRGVTNLLTGSRRKRRDVTPNTQPKCNPNLHYWT

         550       560       570       580       590       600
R AVDEGAAVGLAWIPYFGPAAEGIYIEGVMHNQNGLICGLRQLANETTQALQLFLRATTEL
E TQDEGAAIGLAWIPYFGPAAEGIYTEGLMHNQDGLICGLRQLANETTQALQLFLRATTEL
S AQEQHNAAGIAWIPYFGPGAEGIYTEGLMHNQNALVCGLRQLANETTQALQLFLRATTEL
B TQDEGAAIGLAWIPYFGPAAEGIYTEGIMHNQNGLICGLRQLANETTQALQLFLRATTEL
T ALDEGAAIGLAWIPYFGPAAEGIYTEGIMENQNGLICGLRQLANETTQALQLFLRATTEL

         610       620       630       640       650       660
R RTYSLLNRKAIDFLLQRWGGTCRILGPSCCIEPHDWTKNITDEINQIKHDFIDNPLPDHG
E RTFSILNRKAIDFLLQRWGGTCHILGPDCCIEPHDWTKNITDKIDQIIHDFVDKTLPDQG
S RTYTILNRKAIDFLLRRWGGTCRILGPDCCIEPHDWTKNITDKINQIIHDFIDNPLPNQD
B RTFSILNRKAIDFLLQRWGGTCHILGPDCCIEPHDWTKNITDKIDQIIHDFIDKPLPDQT
T RTFSILNRKAIDFLLQRWGGTCHILGPDCCIEPQDWTKNITDKIDQIIHDFVDNNLPNQN

         670       680       690
R DDLNLWTGWRQWIPAGIGIIGVIIAIIALLCICKILC
E DNDNWWTGWRQWIPAGIGVTGVIIAVIALFCICKFVF
S NDDNWWTGWRQWIPAGIGITGIIIAIIALLCVCKLLC
B DNDNWWTGWRQWVPAGIGITGVIIAVIALLCICKFLL
T DGSNWWTGWKQWVPAGIGITGVIIAIIALLCICKFML
```

C – VP40

```
           10        20        30        40        50        60
R MRRGVLPTAPPAYNDIAYPMSILPTRPSVIVNETKSDVLAVPGADVPSNSMRPVADDNID
E MRRVILPTAPPEYMEAIYPARSNSTIARGGNSNTGFLTPESVNGDTPSNPLRPIADDTID
S MRRVTVPTAPPAYADIGYPMSMLPIKSSRAVSGIQQKQEVLPGMDTPSNSMRPVADDNID
B MRRAILPTAPPEYMEAVYPMRTVSTNISSTSSGPNFPAPDVMMSDTPSNSLRPIADDNID
T MRRIILPTAPPEYMEAVYPMRTMNSGADNTASGPNYTTTGVMTNDTPSNSLRPVADDNID

           70        80        90       100       110       120
R HSSHTPSGVASAFILEATVNVISGTKVLMKQIPIWLPLGVADQKIYSFDSTTAAIMLASY
E HASHTPGSVSSAFILEAMVNVISGPKVLMKQIPIWLPLGVADQKTYSFDSTTAAIMLASY
S HTSHTPNGVASAFILEATVNVISGPKVLMKQIPIWLPLGIADQKTYSFDSTTAAIMLASY
B HPSHTPTSVSSAFILEAMVNVISGPKVLMKQIPIWLPLGVADQKTYSFDSTTAAIMLASY
T HPSHTPNSVASAFILEAMVNVISGPKVLMKQIPIWLPLGVSDQKTYSFDSTTAAIMLASY

          130       140       150       160       170       180
R TVTHFGKISNPLVRVNRLGPGIPDHPLRLLRLGNQAFLQEFVLPPVQLPQYFTFDLTALK
E TITHFGKATNPLVRVNRLGPGIPDHPLRLLRIGNQAFLQEFVLPPVQLPQYFTFDLTALK
S TITHFGKANNPLVRVNRLGQGIPDHPLRLLRMGNQAFLQEFVLPPVQLPQYFTFDLTALK
B TITHFGKTSNPLVRINRLGPGIPDHPLRLLRIGNQAFLQEFVLPPVQLPQYFTFDLTALK
T TITHFGKTSNPLVRINRLGPGIPDHPLRLLRIGNQAFLQEFVLPPVQLPQYFTFDLTALK

          190       200       210       220       230       240
R LITQPLPAATWTDETPAGAVNALRPGLSLHPKLRPILLPGKTGKKGHASDLTSPDKIQTI
E LITQPLPAATWTDDTPTGSNGALRPGISFHPKLRPILLPNKSGKKGNSADLTSPEKIQAI
S LVTQPLPAATWTDETPSNLSGALRPGLSFHPKLRPVLLPGKTGKKGHVSDLTAPDKIQTI
B LITQPLPAATWTDDTPTGPTGILRPGISFHPKLRPILLPGKTGKRGSSSDLTSPDKIQAI
T LITQPLPAATWTDETPAVSTGTLRPGISFHPKLRPILLPGRAGKKGSNSDLTSPDKIQAI

          250       260       270       280       290       300
R MNAIPDLKIVPIDPTKNIVGIEVPELLVQRLTGKKPQPKNGQPIIPVLLPKYVGLDPISP
E MTSLQDFKIVPIDPTKNIMGIEVPETLVHKLTGKKVTSKNGQPIIPVLLPKYIGLDPVAP
S VNLMQDFKIVPIDPAKSIIGIEVPELLVHKLTGKKMSQKNGQPIIPVLLPKYIGLDPISP
B MNFLQDLKLVPIDPAKNIMGIEVPELLVHRLTGKKITTKNGQPIIPILLPKYIGMDPISQ
T MNFLQDLKIVPIDPTKNIMGIEVPELLVHRLTGKKTTTKNGQPIIPILLPKYIGLDPLSQ

          310       320       330
R GDLTMVITQDCDSCHSPASHPYHMDKQNSYQ
E GDLTMVITQDCDTCHSPASLPAVVEK-----
S GDLTMVITPDYDDCHSPASCSYLSEK-----
B GDLTMVITQDCDTCHSPASLPPVSEK-----
T GDLTMVITQDCDSCHSPASLPPVNEK-----
```

D – VP35

```
  1        10        20        30        40        50        60
R ------------MYNNKLKVCSGPETTGWISEQLMTGKIPVTDIFIDIDNKPDQMEVRLK
E -MTTRTKGRGHTVATTQNDRMPGPELSGWISEQLMTGRIPVNDIFCDIENNPGLCYASQM
S ------------MQQDRTYRHHGPEVSGWFSEQLMTGKIPLTEVFVDVENKPSPAPITII
B MTSNRARVTYNPPPTTTGTRSCGPELSGWISEQLMTGKIPITDIFNEIETLPSISPSIHS
T MISTRAAAINDPSLPIRNQCTRGPELSGWISEQLMTGKIPVHEIFNDTEPHISSGSDCLP

           70        80        90       100       110       120
R PSSRSSTRTCTSSSQTEVNYVPLLKKVEDTLTMLVNATSRQNAAIEALENRLSTLESSLK
E QQTKPNPKMRNSQTQTDPICNHSFEEVVQTLASLATVVQQQTIASESLEQRITSLENGLK
S SKNPKTTRKSDKQVQTDDASSLLTEEVKAAINSVISAVRRQTNAIESLEGRVTTLEASLK
B KIKTPSVQTRSVQTQTDPNCNHDFAEVVKMLTSLTLVVQKQTLATESLEQRITDLEGSLK
T RPKNTAPRTRNTQTQTDPVCNHNFEDVTQALTSLTNVIQKQALNLESLEQRIIDLENGLK

          130       140       150       160       170       180
R PIQDMGKVISSLNRSCAEMVAKYDLLVMTTGRATSTAAAVDAYWKEHKQPPPGPALYEEN
E PVYDMAKTISSLNRVCAEMVAKYDLLVMTTGRATATAAATEAYWAEHGQPPPGPSLYEES
S PVQDMAKTISSLNRSCAEMVAKYDLLVMTTGRATATAAATEAYWNEHGQAPPGPSLYEDD
B PVSEITKIVSALNRSCAEMVAKYDLLVMTTGRATATAAATEAYWAEHGRPPPGPSLYEED
T PMYDMAKVISALNRSCAEMVAKYDLLVMTTGRATATAAATEAYWEEHGQPPPGPSLYEES

          190       200       210       220       230       240
R ALKGKIDDPNSYVPDAVQEAYKNLDSTSTLTEENFGKPYISAKDLKEIMYDHLPGFGTAF
E AIRGKIESRDETVPQSVREAFNNLDSTTSLTEENFGKPDISAKDLRNIMYDHLPGFGTAF
S AIKAKLKDPNGKVPESVKQAYTNLDSTSALNEENFGRPYISAKDLKEIIYDHLPGFGTAF
B AIRTKIEKQGDIVPKEVQEAFRNLDSTALLTEENFGKPDISAKDLRNIMYDHLPGFGTAF
T AIRGKINKQEDKVPKEVQEAFRNLDSTSSLTEENFGKPDISAKDLRDIMYDHLPGFGTAF

          250       260       270       280       290       300
R HQLVQVICKIGKDNNLLDTIHAEFQASLADGDSPQCALIQITKRVPIFQDVPPPIIHIRS
E HQLVQVICKLGKDSNSLDIIHAEFQASLAEGDSPQCALIQITKRVPIFQDAAPPVIHIRS
S HQLVQVICKIGKDNNILDIIHAEFQASLAEGDSPQCALIQITKRIPAFQDASPPIVHIKS
B HQLVQVICKLGKDNSSLDVIHAEFQASLAEGDSPQCALIQITKRIPIFQDAAPPVIHIRS
T HQLVQVICKLGKDNSALDIIHAEFQASLAEGDSPQCALIQITKRIPIFQDATPPTIHIRS

          310       320       330       340
R RGDIPRACQKSLRPAPPSPKIDRGWVCLFKMQDGKTLGLKI
E RGDIPRACQKSLRPVPPSPKIDRGWVCVFQLQDGKTLGLKI
S RGDIPKACQKSLRPVPPSPKIDRGWVCIFQFQDGKALGLKI
B RGDIPKACQKSLRPVPPSPKIDRGWVCIFQLQDGKTLGLKI
T RGDIPRACQKSLRPVPPSPKIDRGWVCIFQLQDGKTLGLKI
```

E – VP30

```
 1        10        20        30        40        50        60
R MMEHSRERGRSSNMRHNSREPYENPSRSRSLSRDPNQVDRRQPRSASQIRVPNLFHRKKT
E -MEASYERGRPRAARQHSRDGHDHHVRARSSSRENYRGEYRQSRSASQVRVPTVFHKKRV
S -MERGRERGRSRNSRADQQNSTGPQFRTRSISRDKTTTDYRSSRSTSQVRVPTVFHKKGT
B -MDSFHERGRSRTIRQSARDGPSHQVRTRSSSRDSHRSEYHTPRSSSQVRVPTVFHRKRT
T -MEVVHERGRSRISRQNTRDGPSHLVRARSSSRASYRSEYHTPRSASQIRVPTVFHRKKT

          70        80        90        100       110       120
R DALIVPPAPKDICPTLKKGFLCDSKFCKKDHQLDSLNDHELLLLIARRTCGIIESNSQIT
E EPLTVPPAPKDICPTLKKGFLCDSSFCKKDHQLESLTDRELLLLIARKTCGSVEQQLNIT
S GTLTVPPAPKDICPTLRKGFLCDSNFCKKDHQLESLTDRELLLLIARKTCGSTDSSLNIA
B DFLTVPPAPKDICPTLRKGFLCDSNFCKKDHQLESLTDRELLLLIARKTCGSLEQQLNIT
T DLLTVPPAPKDVCPTLKKGFLCDSNFCKKDHQLESLTDRELLLLIARKTCGSTEQQLSIV

          130       140       150       160       170       180
R SPKDMRLANPTAEDFSQGNSPKLTLAVLLQIAEHWATRDLRQIEDSKLRALLTLCAVLTR
E APKDSRLANPTADDFQQEEGPKITLLTLIKTAEHWARQDIRTIEDSKLRALLTLCAVMTR
S APKDLRLANPTADDFKQDGSPKLTLKLLVETAEFWANQNINEVDDAKLRALLTLSAVLVR
B APKDTRLANPIADDFQQKDGPKITLLTLLETAEYWSKQDIKGIDDSRLRALLTLCAVMTR
T APKDSRLANPIAEDFQQKDGPKVTLSMLIETAEYWSKQDIKNIDDSRLRALLTLCAVMTR

          190       200       210       220       230       240
R KFSKSQLGLLCETHLRHEGLGQDQADSVLEVYQRLHSDKGGNFEAALWQQWDRQSLIMFI
E KFSKSQLSLLCETHLRREGLGQDQAEPVLEVYQRLHSDKGGSFEAALWQQWDRQSLIMFI
S KFSKSQLSQLCESHLRRENLGQDQAESVLEVYQRLHSDKGGAFEAALWQQWDRQSLTMFI
B KFSKSQLSLLCESHLRREGLGQDQSESVLEVYQRLHSDKGGNFEAALWQQWDRQSLIMFI
T KFSKSQLSLLCESHLRREGLGQDQSESVLEVYQRLHSDKGGNFEAALWQQWDRQSLIMFI

          250       260       270       280       290
R SAFLNIALQIPCESSSVVVSGLATLYPAQDNSTPSEATNDTTWSSTVE--
E TAFLNIALQLPCESSAVVVSGLRTLVPQSDNEEASTNPGTCSWSDEGTP-
S SAFLHVALQLSCESSTVVISGLRLLAPPSVNEGLPPAPGEYTWSEDSTT-
B TAFLNIALQLPCESSSVVISGLRLLVPQSEDTETSTYTETRAWSEEGGPH
T TAFLNIALQLPCESSSVVISGLRMLIPQSEATEVVTPSETCTWSEGGSSH
```

F – sGP

```
                  10        20        30        40        50        60
R ---------------------------------------------MGSGYQLLQLPRERF
E ----------------------------------------------MGVTGILQLPRDRF
S ----------------------------------------------MGGLSLLQLPRDKF
B ----------------------------------------------MVTSGILQLPRERF
T ----------------------------------------------MGASGILQLPRERF

                  70        80        90       100       110       120
R RKTSFLVWVIILFQRAISMPLGIVTNSTLKATEIDQLVCRDKLSSTSQLKSVGLNLEGNG
E RKTSFFLWVIILFQRTFSIPLGVIHNSTLQVSDVDKLVCRDKLSSTNQLRSVGLNLEGNG
S RKSSFFVWVIILFQKAFSMPLGVVTNSTLEVTEIDQLVCKDHLASTDQLKSVGLNLEGSG
B RKTSFFVWVIILFHKVFPIPLGVVHNNTLQVSDIDKLVCRDKLSSTSQLKSVGLNLEGNG
T RKTSFFVWVIILFHKVFSIPLGVVHNNTLQVSDIDKFVCRDKLSSTSQLKSVGLNLEGNG

                 130       140       150       160       170       180
R IATDVPSATKRWGFRSGVPPKVVSYEAGEWAENCYNLEIKKSDGSECLPLPPDGVRGFPR
E VATDVPSVTKRWGFRSGVPPKVVNYEAGEWAENCYNLEIKKPDGSECLPAAPDGIRGFPR
S VSTDIPSATKRWGFRSGVPPKVVSYEAGEWAENCYNLEIKKPDGSECLPPPPDGVRGFPR
B VATDVPTATKRWGFRAGVPPKVVNYEAGEWAENCYNLDIKKADGSECLPEAPEGVRGFPR
T VATDVPTATKRWGFRAGVPPKVVNCEAGEWAENCYNLAIKKVDGSECLPEAPEGVRDFPR

                 190       200       210       220       230       240
R CRYVHKVQGTGPCPGDLAFHKNGAFFLYDRLASTVIYRGTTFAEGVVAFLILSEPKKHFW
E CRYVHKVSGTGPCAGDFAFHKEGAFFLYDRLASTVIYRGTTFAEGVVAFLILPQAKKDFF
S CRYVHKAQGTGPCPGDYAFHKDGAFFLYDRLASTVIYRGVNFAEGVIAFLILAKPKETFL
B CRYVHKVSGTGPCPEGYAFHKEGAFFLYDRLASTIIYRSTTFSEGVVAFLILPETKKDFF
T CRYVHKVSGTGPCPGGLAFHKEGAFFLYDRLASTIIYRGTTFAEGVIAFLILPKARKDFF

                 250       260       270       280       290       300
R KATPAHEPVNTTDDSTSYYMTLTLSYEMSNFGGEESNTLFKVDNHTYVQLDRPHTPQFLV
E SSHPLREPVNATEDPSSGYYSTTIRYQATGFGTNETEYLFEVDNLTYVQLESRFTPQFLL
S QSPPIREAVNYTENTSSYYATSYLEYEIENFGAQHSTTLFKIDNNTFVRLDRPHTPQFLF
B QSPPLHEPANMTTDPSSYYHTVTLNYVADNFGTNMTNFLFQVDHLTYVQLEPRFTPQFLV
T QSPPLHEPANMTTDPSSYYHTTTINYVVDNFGTNTTEFLFQVDHLTYVQLEARFTPQFLV

                 310       320       330       340       350       360
R QLNETLRRNNRLSNSTGRLTWTLDPKIEPDVGEWAFWETKKTFPNNFMEKTCISKFYQPT
E QLNETIYASGKRSNTTGKLIWKVNPEIDTTIGEWAFWETKKTSLEKFAVKSCLSQLYQT-
S QLNDTIHLHQQLSNTTGRLIWTLDANINADIGEWAFWENKKISPNNYVEKSCLSKLYRST
B QLNETIYTNGRRSNTTGTLIWKVNPTVDTGVGEWAFWENKKTSQNPFQ--S--S------
T LLNETIYSDNRRSNTTGKLIWKINPTVDTSMGEWAFWENKKTHQNPFQ------------

                 370       380       390       400       410       420
R PTTPQIRARRELSKEKLATTHPPTTPSWFQRIPLQWFQCSLQDGQRKCRPKV--------
E PKTSVVRVRRELLPTQ-PTQQ-KTTKSWLQKIPLQWFKCTVKEGKLQCRI----------
S RQKTMMRHRRELQREESPTGPPGSIRTWFQRIPLGWFHCTYQKGKQHCRLRIRQKVEE--
B ---AA-----S---AS------------F-------F----S--------H----S----
T ------------------------------------------------------------
```

G – NP

```
                                       10                                      20                                      30                                      40                                      50                                      60
R MDRGTRRIWVSQNQGDTDLDYHKILTAGLTVQQGIVRQKIISVYLVDNLEAMCQLVIQAF
E MDSRPQKVWMTPSLTESDMDYHKILTAGLSVQQGIVRQRVIPVYQVNNLEEICQLIIQAF
S MDKRVRGSWALGGQSEVDLDYHKILTAGLSVQQGIVRQRVIPVYVVSDLEGICQHIIQAF
B MDPRPIRTWMMHNTSEVEADYHKILTAGLSVQQGIVRQRIIPVYQISNLEEVCQLIIQAF
T MESRAHKAWMTHTASGFETDYHKILTAGLSVQQGIVRQRVIQVHQVTNLEEICQLIIQAF

  70 80 90 100 110 120
R EAGIDFQENADSFLLMLCLHHAYQGDYKLFLESNAVQYLEGHGFKFELRKKDGVNRLEEL
E EAGVDFQESADSFLLMLCLHHAYQGDYKLFLESGAVKYLEGHGFRFEVKKCDGVKRLEEL
S EAGVDFQDNADSFLLLLCLHHAYQGDHRLFLKSDAVQYLEGHGFRFEVREKENVHRLDEL
B EAGVDFQDSADSFLLMLCLHHAYQGDYKQFLESNAVKYLEGHGFRFEMKKKEGVKRLEEL
T EAGVDFQESADSFLLMLCLHHAYQGDYKQFLESNAVKYLEGHGFRFEVRKKEGVKRLEEL

  130 140 150 160 170 180
R LPAATSGKNIRRTLAALPEEETTEANAGQFLSFASLFLPKLVVGEKACLEKVQRQIQVHA
E LPAVSSGRNIKRTLAAMPEEETTEANAGQFLSFASLFLPKLVVGEKACLEKVQRQIQVHA
S LPNVTGGKNLRRTLAAMPEEETTEANAGQFLSFASLFLPKLVVGEKACLEKVQRQIQVHA
B LPAASSGKNIKRTLAAMPEEETTEANAGQFLSFASLFLPKLVVGEKACLEKVQRQIQVHA
T LPAASSGKSIRRTLAAMPEEETTEANAGQFLSFASLFLPKLVVGEKACLEKVQRQIQVHS

  190 200 210 220 230 240
R EQGLIQYPTAWQSVGHMMVIFRLMRTNFLIKYLLIHQGMHMVAGHDANDAVIANSVAQAR
E EQGLIQYPTAWQSVGHMMVIFRLMRTNFLIKFLLIHQGMHMVAGHDANDAVISNSVAQAR
S EQGLIQYPTSWQSVGHMMVIFRLMRTNFLIKFLLIHQGMHMVAGHDANDTVISNSVAQAR
B EQGLIQYPTSWQSVGHMMVIFRLMRTNFLIKFLLIHQGMHMVAGHDANDAVIANSVAQAR
T EQGLIQYPTAWQSVGHMMVIFRLMRTNFLIKFLLIHQGMHMVAGHDANDAVIANSVAQAR

  250 260 270 280 290 300
R FSGLLIVKTVLDHILQKTDQGVRLHPLARTAKVRNEVNAFKAALSSLAKHGEYAPFARLL
E FSGLLIVKTVLDHILQKTERGVRLHPLARTAKVKNEVNSFKAALSSLAKHGEYAPFARLL
S FSGLLIVKTVLDHILQKTDLGVRLHPLARTAKVKNEVSSFKAALGSLAKHGEYAPFARLL
B FSGLLIVKTVLDHILQKTEHGVRLHPLARTAKVKNEVSSFKAALASLAQHGEYAPFARLL
T FSGLLIVKTVLDHILQKTEHGVRLHPLARTAKVKNEVNSFKAALSSLAQHGEYAPFARLL

  310 320 330 340 350 360
R NLSGVNNLEHGLYPQLSAIALGVATAHGSTLAGVNVGEQYQQLREAATEAEKQLQQYAES
E NLSGVNNLEHGLFPQLSAIALGVATAHGSTLAGVNVGEQYQQLREAATEAEKQLQQYAES
S NLSGVNNLEHGLYPQLSAIALGVATAHGSTLAGVNVGEQYQQLREAATEAEKQLQQYAET
B NLSGVNNLEHGLFPQLSAIALGVATAHGSTLAGVNVGEQYQQLREAATEAEKQLQKYAES
T NLSGVNNLEHGLFPQLSAIALGVATAHGSTLAGVNVGEQYQQLREAATEAEKQLQKYAES

  370 380 390 400 410 420
R RELDSLGLDDQERRILMNFHQKKNEISFQQTNAMVTLRKERLAKLTEAITLASRPNLGSR
E RELDHLGLDDQEKKILMNFHQKKNEISFQQTNAMVTLRKERLAKLTEAITAASLPKTSGH
S RELDNLGLDEQEKKILMSFHQKKNEISFQQTNAMVTLRKERLAKLTEAITTASKIKVGDR
B RELDHLGLDDQEKKILKDFHQKKNEISFQQTTAMVTLRKERLAKLTEAITSTSILKTGRR
T RELDHLGLDDQEKKILKDFHQKKNEISFQQTTAMVTLRKERLAKLTEAITSTSLLKTGKQ
```

```
         430       440       450       460       470       480
R QDDGNEIPFPGPISNNPDQDHLEDDPRDSRDTIIPNGAIDPEDGDFENYNGYHDDEVGTA
E YDDDDDIPFPGPINDDDNPGHQDDDPTDSQDTTIPDVVVDPDDGGYGEYQSYSENGMSAP
S YPDDNDIPFPGPIYDETHPNPSDDNPDDSRDTTIPGGVVDPYDDESNNYPDYEDSAEGTT
B YDDDNDIPFPGPINDNENSGQNDDDPTDSQDTTIPDVIIDPNDGGYNNYSDYANDAASAP
T YDDDNDIPFPGPINDNENSEQQDDDPTDSQDTTIPDIIVDPDDGRYNNYGDYPSETANAP

         490       500       510       520       530       540
R GDLVLFDLDDHEDDNKAFEPQDSSPQSQREIERERLIHPPPGNNKDDNRAS------DN-
E DDLVLFDLDEDDEDTKPVPNRSTKGGQQKNSQK--------GQHTE-GRQTQSTPTQNVT
S GDLDLFNLDDDDDDSQPGPPDRGQSKER-AARTHGLQDPTL---DGAKKVPELTPGSHQP
B DDLVLFDLEDEDDADNPAQN---TPEKNDR---PATTKLRNGRDQDGNQSETASPRAAP-
T EDLVLFDLEDGDEDDHRPSS---SSENNNK---HSLTGTDSNKTSNWNRNPTNMPKKDS-

         550       560       570       580       590       600
R -NQQ-SADSEEQGGQYNWHRGPERTTANRRLSPVHEEDTLMDQGDDDPSSLPPLESDDDD
E GPRRTIHHASAPLTDNDRRNEPSGSTSPRMLTPINEEADPLDDADDETSSLPPLESDDEE
S GNLH-ITKPGS----NTNQPQGNMSSTLQSMTPIQEESEPDDQKDDDDESLTSLDSEGDE
B -NQY-RDKPMPQVQSRSENHDQTLQTQPRVLTPISEEADPSDHNDGDNESIPPLESDDEG
T -TQN-NDNPAQRAQEYARDNIQDTPTPHRALTPISEETGSNGHNEDDIDSIPPLESDEEN

         610       620       630       640       650       660
R ASSSQQDPDYTAVAPPAPVYRSAEAHEPPHKSSNEPAETSQL-NEDPDIGQSKSMQKLEE
E QDRDGTSNRTPTVAPPAPVYRDHSEKKELPQDEQQDQDHI-QEARNQDSDNTQPEHSFEE
S DVESVSGENNPTVAPPAPVYKDTGVDTNQQNGP-SNAVDGQGSESEALPINPEKRSALEE
B STDTTAAETKPATAPPAPVYRSISVDDSVPLEN-IPAQSNQTNNEDNVRNNAQSEQSIAE
T NTETTITTTKNTTAPPAPVYRSNSEKEPLPQEK-SQKQPNQVSGSENTDNKPHSEQSVEE

         670       680       690       700       710       720
R TYHHLLRTQGPFEAINYYHMMKDEPVIFSTDDGKEYTYPDSLEEAYPPWLTEKERLDKEN
E MYRHILRSQGPFDAVLYYHMMKDEPVVFSTSDGKEYTYPDSLEEEYPPWLTEKEAMNDEN
S TYYHLLKTQGPFEAINYYHLMSDEPIAFSTESGKEYIFPDSLEEAYPPWLSEKEALEKEN
B MYQHILKTQGPFDAILYYHMMKEEPIIFSTSDGKEYTYPDSLEDEYPPWLSEKEAMNEDN
T MYRHILQTQGPFDAILYYYMMTEEPIVFSTSDGKEYVYPDSLEGEHPPWLSEKEALNEDN

         730       740
R RYIYINNQQFFWPVMSPRDKFLAILQHHQ
E RFVTLDGQQFYWPVMNHRNKFMAILQHHQ
S RYLVIDGQQFLWPVMSLQDKFLAVLQHD-
B RFITMDGQQFYWPVMNHRNKFMAILQHHR
T RFITMDDQQFYWPVMNHRNKFMAILQHHK
```

H – L

```
  1       10        20        30        40        50        60
R -MATQHTQYPDARLSSPIVLDQCDLVTRACGLYSSYSLNPQLRQCKLPKHIYRLKFDTIV
E -MATQHTQYPDARLSSPIVLDQCDLVTRACGLYSSYSLNPQLRNCKLPKHIYRLKYDVTV
S MMATQHTQYPDARLSSPIVLDQCDLVTRACGLYSEYSLNPKLRTCRLPKHIYRLKYDTIV
B -MATQHTQYPDARLSSPIVLDQCDLVTRACGLYSSYSLNPQLKNCRLPKHIYRLKFDATV
T -MATQHTQYPDARLSSPIVLDQCDLVTRACGLYSAYSLNPQLKNCRLPKHIYRLKYDTTV

          70        80        90        100       110       120
R SKFLSDTPVATLPIDYLVPILLRSLTGHGDRPLTPTCNQFLDEIINYTLHDAAFLDYYLK
E TKFLSDVPVATLPIDFIVPILLKALSGNGFCPVEPRCQQFLDEIIKYTMQDALFLKYYLK
S LRFISDVPVATIPIDYIAPMLINVLADSKNVPLEPPCLSFLDEIVNYTVQDAAFLNYYMN
B TKFLSDVPIVTLPIDYLTPLLLRTLSGEGLCPVEPKCSQFLDEIVSYVLQDARFLRHYFR
T TEFLSDVPVATLPADFLVPTFLRTLSGNGSCPIDPKCSQFLEEIVNYTLQDIRFLNYYLN

          130       140       150       160       170       180
R ATGAQDHLTNIATREKLKNEILNNDYVHQLFFWHDLSILARRGRLNRGNNRSTWFVHDEF
E NVGAQEDCVDDHFQEKILSSIQGNEFLHQMFFWYDLAILTRRGRLNRGNSRSTWFVHDDL
S QIKTQEGVITDQLKQNIRRVIHKNRYLSALFFWHDLAILTRRGRMNRGNVRSTWFVTNEV
B HVGVHDDNVGKNFEPKIKALIYDNEFLQQLFYWYDLAILTRRGRLNRGNNRSTWFANDDL
T RAGVHNDHVDRDFGQKIRNLICDNEVLHQMFHWYDLAILARRGRLNRGNNRSTWFASDNL

          190       200       210       220       230       240
R IDILGYGDYIFWKIPLSLLPVTIDGVPHAATDWYQPTLFKESILGHSQILSVSTAEILIM
E IDILGYGDYVFWKIPISLLPLNTQGIPHAAMDWYQTSVFKEAVQGHTHIVSVSTADVLIM
S VDILGYGDYIFWKIPIALLPMNTANVPHASTDWYQPNIFKEAIQGHTHIISVSTAEVLIM
B IDILGYGDYIFWKIPLSLLSLNTEGIPHAAKDWYHASIFKEAVQGHTHIVSVSTADVLIM
T VDILGYGDYIFWKIPLSLLPVDTQGLPHAAKDWYHESVFKEAIQGHTHIVSISTADVLIM

          250       260       270       280       290       300
R CKDIITCRFNTSLIASIAKLEDVDVSDYPDPSDILKIYNAGDYVISILGSEGYKIIKYLE
E CKDLITCRFNTTLISKIAEVEDPVCSDYPNFKIVSMLYQSGDYLLSILGSDGYKIIKFLE
S CKDLVTSRFNTLLIAELARLEDPVSADYPLVDNIQSLYNAGDYLLSILGSEGYKIIKYLE
B CKDIITCRFNTTLIAALANLEDSICSDYPQPETISNLYKAGDYLISILGSEGYKVIKFLE
T CKDIITCRFNTLLIAAVANLEDSVHSDYPLPETVSDLYKAGDYLISLLGSEGYKVIKFLE

          310       320       330       340       350       360
R PLCLAKIQLCSKFTERKGRFLTQMHLSVINDLRELISNRRLKDYQQEKIRDFHKILLQLQ
E PLCLAKIQLCSKYTERKGRFLTQMHLAVNHTLEEITEIRALKPSQAHKIREFHRTLIRLE
S PLCLAKIQLCSQYTERKGRFLTQMHLAVIQTLRELLLNRGLKKSQLSKIREFHQLLLRLR
B PLCLAKIQLCSNYTERKGRFLTQMHLAVNHTLEELIEGRGLKSQQDWKMREFHRILVNLK
T PLCLAKIQLCSNYTERKGRFLTQMHLAVNHTLEELTGSRELRPQQIRKVREFHQMLINLK

          370       380       390       400       410       420
R LSPQQFCELFSVQKHWGHPILHSEKAIQKVKRHATILKALRPNVIFETYCVFKYNIAKHY
E MTPQQLCELFSIQKHWGHPVLHSETAIQKVKKHATVLKALRPIVIFETYCVFKYSIAKHY
S STPQQLCELFSIQKHWGHPVLHSEKAIQKVKNHATVLKALRPIIIFETYCVFKYSVAKHF
B STPQQLCELFSVQKHWGHPVLHSEKAIQKVKKHATIIKALRPIIIFETYCVFKYSIAKHY
T ATPQQLCELFSVQKHWGHPVLHSEKAIQKVKKHATVIKALRPIIIFETYCVFKYSIAKHY
```

```
         430       440       450       460       470       480
R FDSQGTWYSVISDRNLTPGLNSFIKRNHFPSLPMIKDLLWEFYHLNHPPLFSTKVISDLS
E FDSQGSWYSVTSDRNLTPGLNSYIKRNQFPPLPMIKELLWEFYHLDHPPLFSTKIISDLS
S FDSQGTWYSVISDRCLTPGLNSYIRRNQFPPLPMIKDLLWEFYHLDHPPLFSTKIISDLS
B FDSQGSWYSVISDKHLTPGLHSYIKRNQFPPLPMIKDLLWEFYHLDHPPLFSTKIISDLS
T FDSQGTWYSVTSDRCLTPGLSSYIKRNQFPPLPMIKELLWEFYHLDHPPLFSTKVISDLS

         490       500       510       520       530       540
R IFIKDRATAVEQTCWDAVFEPNVLGYNPPNKFSTKRVPEQFLEQEDFSIESVLNYAQELH
E IFIKDRATAVERTCWDAVFEPNVLGYNPPHKFSTKRVPEQFLEQENFSIENVLSYAQKLE
S IFIKDRATAVEQTCWDAVFEPNVLGYSPPYRFNTKRVPEQFLEQEDFSIESVLQYAQELR
B IFIKDRATAVEKTCWDAVFEPNVLGYSPPNKFSTKRVPEQFLEQENFSIDSVLTYAQRLD
T IFIKDRATAVEKTCWDAVFEPNVLGYNPPNKFATKRVPEQFLEQENFSIESVLHYAQRLE

         550       560       570       580       590       600
R YLLPQNRNFSFSLKEKELNIGRTFGKLPYLTRNVQTLCEALLADGLAKAFPSNMMVVTER
E YLLPQYRNFSFSLKEKELNVGRTFGKLPYPTRNVQTLCEALLADGLAKAFPSNMMVVTER
S YLLPQNRNFSFSLKEKELNVGRTFGKLPYLTRNVQTLCEALLADGLAKAFPSNMMVVTER
B YLLPQYRNFSFSLKEKELNVGRAFGKLPYPTRNVQTLCEALLADGLAKAFPSNMMVVTER
T YLLPEYRNFSFSLKEKELNIGRAFGKLPYPTRNVQTLCEALLADGLAKAFPSNMMVVTER

         610       620       630       640       650       660
R EQKESLLHQASWHHTSDDFGENATVRGSSFVTDLEKYNLAFRYEFTAPFIEYCNHCYGVR
E EQKESLLHQASWHHTSDDFGEHATVRGSSFVTDLEKYNLAFRYEFTAPFIEYCNRCYGVK
S EQKESLLHQASWHHTSDDFGEHATVRGSSFVTDLEKYNLAFRYEFTAPFIKYCNQCYGVR
B EQKESLLHQASWHHTSDDFGENATVRGSSFVTDLEKYNLAFRYEFTAPFIEYCNRCYGVK
T EQKESLLHQASWHHTSDDFGENATVRGSSFVTDLEKYNLAFRYEFTAPFIEYCNRCYGVR

         670       680       690       700       710       720
R NVFNWMHYLIPQCYMHVSDYYNPPHNVNLSNREYPPEGPSSYRGHLGGIEGLQQKLWTSI
E NVFNWMHYTIPQCYMHVSDYYNPPHNLTLENRNNPPEGPSSYRGHMGGIEGLQQKLWTSI
S NVFDWMHFLIPQCYMHVSDYYNPPHNVTLENREYPPEGPSAYRGHLGGIEGLQQKLWTSI
B NLFNWMHYTIPQCYIHVSDYYNPPHGVSLENREDPPEGPSSYRGHLGGIEGLQQKLWTSI
T NLFNWMHYTIPQCYIHVSDYYNPPHGVSLENRENPPEGPSSYRGHLGGIEGLQQKLWTSI

         730       740       750       760       770       780
R SCAQISLVEIKTGFKLRSAVMGDNQCITVLSVFPLETDPEEQEQSAEDNAARVAASLAKV
E SCAQISLVEIKTGFKLRSAVMGDNQCITVLSVFPLETDAGEQEQSAEDNAARVAASLAKV
S SCAQISLVEIKTGFKLRSAVMGDNQCITVLSVFPLESSPNEQERCAEDNAARVAASLAKV
B SCAQISLVEIKTGFKLRSAVMGDNQCITVLSVFPLETDSNEQEHSSEDNAARVAASLAKV
T SCAQISLVEIKTGFKLRSAVMGDNQCITVLSVFPLETESSEQELSSEDNAARVAASLAKV

         790       800       810       820       830       840
R TSACGIFLKPDETFVHSGFIYFGKKQYLNGVQLPQSLKTAARMAPLSDAIFDDLQGTLAS
E TSACGIFLKPDETFVHSGFIYFGKKQYLNGVQLPQSLKTATRMAPLSDAIFDDLQGTLAS
S TSACGIFLKPDETFVHSGFIYFGKKQYLNGIQLPQSLKTAARMAPLSDAIFDDLQGTLAS
B TSACGIFLKPDETFVHSGFIYFGKKQYLNGVQLPQSLKTATRIAPLSDAIFDDLQGTLAS
T TSACGIFLKPDETFVHSGFIYFGKKQYLNGVQLPQSLKTATRIAPLSDAIFDDLQGTLAS
```

```
                 850       860       870       880       890       900
R IGTAFERAISETRHILPCRIVAAFHTYFAVRILQYHHLGFNKGIDLGQLSLSKPLDYGTI
E IGTAFERSISETRHIFPCRITAAFHTFFSVRILQYHHLGFNKGFDLGQLTLGKPLDFGTI
S IGTAFERSISETRHILPCRVAAAFHTYFSVRILQHHHLGFHKGSDLGQLAINKPLDFGTI
B IGTAFERSISETRHVYPCRVVAAFHTFFSVRILQYHHLGFNKGTDLGQLSLSKPLDFGTI
T IGTAFERSISETRHVVPCRVAAAFHTFFSVRILQYHHLGFNKGTDLGQLSLSKPLDFGTI

                 910       920       930       940       950       960
R TLTLAVPQVLGGLSFLNPEKCFYRNFGDPVTSGLFQLRVYLEMVNMKDLFCPLISKNPGN
E SLALAVPQVLGGLSFLNPEKCFYRNLGDPVTSGLFQLKTYLRMIEMDDLFLPLIAKNPGN
S ALSLAVPQVLGGLSFLNPEKCLYRNLGDPVTSGLFQLKHYLSMVGMSDIFHALVAKSPGN
B TLALAVPQVLGGLSFLNPEKCFYRNLGDPVTSGLFQLRTYLQMINMDDLFLPLIAKNPGN
T TLALAVPQVLGGLSFLNPEKCFYRNLGDPVTSGLFQLKTYLQMIHMDDLFLPLIAKNPGN

                 970       980       990      1000      1010      1020
R CSAIDFVLNPSGLNVPGSQDLTSFLRQIVRRSITLTARNKLINTLFHASADLEDEMVCKW
E CTAIDFVLNPSGLNVPGSQDLTSFLRQIVRRTITLSAKNKLINTLFHASADFEDEMVCKW
S CSAIDFVLNPGGLNVPGSQDLTSFLRQIVRRSITLSARNKLINTLFHASADLEDELVCKW
B CSAIDFVLNPSGLNVPGSQDLTSFLRQIVRRTITLSAKNKLINTLFHSSADLEDEMVCKW
T CSAIDFVLNPSGLNVPGSQDLTSFLRQIVRRTITLSAKNKLINTLFHSSADLEDEMVCKW

                1030      1040      1050      1060      1070      1080
R LLSSNPVMSRFAADIFSRTPSGKRLQILGYLEGTRTLLASKIINNNSETPVLDKLRKITL
E LLSSTPVMSRFAADIFSRTPSGKRLQILGYLEGTRTLLASKIINNNTETPVLDRLRKITL
S LLSSTPVMSRFAADIFSRTPSGKRLQILGYLEGTRTLLASKMISNNAETPILERLRKITL
B LLSSTPVMSRFAADIFSRTPSGKRLQILGYLEGTRTLLASKVINNNAETPILDRLRKITL
T LLSSTPVMSRFAADIFSRTPSGKRLQILGYLEGTRTLLASKIINHNTETPILDRLRKITL

                1090      1100      1110      1120      1130      1140
R QRWNLWFSYLDHCDQLLADALQKISCTVDLAQILREYTWSHILEGRSLIGATLPCMVEQF
E QRWSLWFSYLDHCDNILAEALTQITCTVDLAQILREYSWAHILEGRPLIGATLPCMIEQF
S QRWNLWFSYLDHCDPALMEAIQPIKCTVDIAQILREYSWAHILDGRQLIGATLPCIPEQF
B QRWSLWFSYLDHCDQVLADALIKVSCTVDLAQILREYTWAHILEGRQLIGATLPCMLEQF
T QRWSLWFSYLDHCDQVLADALTQITCTVDLAQILREYTWAHILEGRQLIGATLPCILEQL

                1150      1160      1170      1180      1190      1200
R KVKWLGQYEPCPECLNKKG--SNAYVSVAVKDQVVSAWPNTSRISWTIGSGVPYIGSRTE
E KVVWLKPYEQCPQCSNAKQPGGKPFVSVAVKKHIVSAWPNASRISWTIGDGIPYIGSRTE
S QTTWLKPYEQCVECSSTNN--SSPYVSVALKRNVVSAWPDASRLGWTIGDGIPYIGSRTE
B NVFWLKSYEQCPKCARSRNPKGEPFVSIAIKKQVVSAWPNQSRLNWTIGDGVPYIGSRTE
T NVIWLKPYEHCPKCAKSANPKGEPFVSIAIKKHVVSAWPDQSRLSWTIGDGIPYIGSRTE

                1210      1220      1230      1240      1250      1260
R DKIGQPAIKPRCPSSALKEAIELASRLTWVTQGGSNSEQLIRPFLEARVNLSVSEVLQMT
E DKIGQPAIKPKCPSAALREAIELASRLTWVTQGSSNSDLLIKPFLEARVNLSVQEILQMT
S DKIGQPAIKPRCPSAALREAIELTSRLTWVTQGSANSDQLIRPFLEARVNLSVQEILQMT
B DKIGQPAIKPKCPSAALREAIELTSRLTWVTQGGANSDLLVKPFVEARVNLSVQEILQMT
T DKIGQPAIKPKCPSAALREAIELTSRLTWVTQGGANSDLLVKPFIEARVNLSVQEILQMT
```

```
             1270      1280      1290      1300      1310      1320
R PSHYSGNIVHRYNDQYSPHSFMANRMSNTATRLIVSTNTLGEFSGGGQAARDSNIIFQNV
E PSHYSGNIVHRYNDQYSPHSFMANRMSNSATRLIVSTNTLGEFSGGGQSARDSNIIFQNV
S PSHYSGNIVHRYNDQYSPHSFMANRMSNTATRLMVSTNTLGEFSGGGQAARDSNIIFQNV
B PSHYSGNIVHRYNDQYSPHSFMANRMSNSATRLVVSTNTLGEFSGGGQSARDSNIIFQNV
T PSHYSGNIVHRYNDQYSPHSFMANRMSNSATRLVVSTNTLGEFSGGGQSARDSNIIFQNV

             1330      1340      1350      1360      1370      1380
R INLAVALYDIRFRNTNTSDIRHNRAHLHLTECCTKEVPAQYLTYTSALNLDLSRYRDNEL
E INYAVALFDIKFRNTEATDIQYNRAHLHLTKCCTREVPAQYLTYTSTLDLDLTRYRENEL
S INFAVALYDIRFRNTCTSSIQYHRAHIHLTNCCTREVPAQYLTYTTTLNLDLSKYRNNEL
B INFAVALFDLRFRNTETSSIQHNRAHLHLSQCCTREVPAQYLTYTSTLSLDLTRYRENEL
T INFAVALFDLRFRNVATSSIQHHRAHLHLSKCCTREVPAQYLVYTSTLPLDLTRYRDNEL

             1390      1400      1410      1420      1430      1440
R IYDSNPLKGGLNCNLTIDSPLVKGPRLNMIEDDLLRFPHLSGWELAKTVVQSIISDNSNS
E IYDNNPLKGGLNCNISFDNPFFQGKQLNIIEDDLIRLPHLSGWELAKTIMQSIISDSNNS
S IYDSEPLRGGLNCNLSIDSPLMKGPRLNIIEDDLIRLPHLSGWELAKTVLQSIISDSSNS
B IYDNNPLKGGLNCNLSFDNPLFKGQRLNIIEEDLIRFPHLSGWELAKTIIQSIISDSNNS
T IYDDNPLRGGLNCNLSFDNPLFKGQRLNIIEEDLIRLPYLSGWELAKTVIQSIISDSNNS

             1450      1460      1470      1480      1490      1500
R STDPISSGETRSFTTHFLTYPQIGLLYSFGAVLCFYLGNTILWTKKLDYEQFLYYLHNQL
E STDPISSGETRSFTTHFLTYPKIGLLYSFGAFVSYYLGNTILRTKKLTLDNFLYYLTTQI
S STDPISSGETRSFTTHFLTYPKIGLLYSFGALISFYLGNTILCTKKIGLTEFLYYLQNQI
B STDPISSGETRSFTTHFLTYPKVGLLYSFGAIVSYYLGNTIIRTKKLDLSHFMYYLTTQI
T STDPISSGETRSFTTHFLTYPKIGLLYSFGALISYYLGNTIIRTKKLTLNNFIYYLATQI

             1510      1520      1530      1540      1550      1560
R HNLPHRALRVFKPTFKHASVMSRLMEIDSNFSIYIGGTSGDRGLSDAARLFLRTAIASFL
E HNLPHRSLRILKPTFKHASVMSRLMSIDPHFSIYIGGAAGDRGLSDAARLFLRTSISSFL
S HNLSHRSLRIFKPTFRHSSVMSRLMDIDPNFSIYIGGTAGDRGLSDAARLFLRIAISTFL
B HNLPHRSLRILKPTFKHVSVISRLMSIDPHFSIYIGGTAGDRGLSDATRLFLRVAISSFL
T HNLPHRSLRILKPTLKHASVISRLISIDSHFSIYIGGTAGDRGLSDAARLFLRTAITVFL

             1570      1580      1590      1600      1610      1620
R QFLKSWIIDRQKTIPLWIVYPLEGQQPESINEFLHKILGLLKQGPKSIPKEVSIQNDGHL
E TFVKEWIINRGTIVPLWIVYPLEGQNPTPVNNFLHQIVELLVHDSSRHQAFK--TTINDH
S SFVEEWVIFRKANIPLWVIYPLEGQRPDPPGEFLNRVKSLIVGTEDDKNKGSIL--SRSG
B QFVKKWIVEYRTAIPLWVVYPLEGQNPDPINSFLHQIIALLQNESP--QNNIQFQEGRNN
T QFVRKWIVERKTAIPLWVIYPLEGQSPSPINSFLHHVIALLQHESS--HDHVCAAEAHSR

             1630      1640      1650      1660      1670      1680
R DLAENNYVYNSKSTASNFFHASLAYWRSRKSRKTQDHNDFSRGDGTL----TEPVRKFSS
E VHPHDNLVYTCKSTASNFFHASLAYWRSRHRNSNRKDLTRNSSTGSSTNNSDGHIKRSQE
S EKCSSNLVYNCKSTASNFFHASLAYWRGRHRPKKTIGATNATTAPHI----ILPLGNSDR
B QQLSDNLVYMCKSTASNFFHASLAYWRSRHKGRPKNRSTEEQTVKPRPYNNFHSVKCASN
T VETFDNLVYMCKSTASNFFHASLAYWRSRSKNQDKREMTKILSLTQTEKKN--SFGYTAH
```

```
       1690      1700      1710      1720      1730      1740
R -----NHQSDEKYYNVTCGKSPKPQERKDF--SQYRLSNNGQTMSNHRKKGKFHKWNPCK
E QT---------------TRDPHDGTERSLVLQMSHEIKRTTIPQ------ENTHQGPSFQ
S PPGLDLNRNNDTFIPTRIKQIVQGDSRNDRT-TTTRFPPKSRS------TPTSATEPPTK
B PPSIP--KSKSGT----QGSSA-FFEKLEYD-KEIELPTASTP---AEKPKTYTKALSSR
T PESTAVLGSLQTS----LAPPP-SADEATYD-RKNKVLKASRP---GKYSQNTTKAPPNQ

       1750      1760      1770      1780      1790      1800
R MLMESQRGTVL------------TEGDYFQNNTPPTDDVSSPHRLILPFFKLGNHNHAHD
E SFLSDSACGTANPKLNFDRSRHNVKSQDHNSASKREGHQIISHRLVLPFFTLSQGTRQLT
S MYEGSTTHQGK------------LTDTHLDEDHNAKEFPSNPHRLVVPFFKLTKDGEYSI
B IYHGKTPSNAAKDDSTT-----SKGCDS-----KEENAVQASHRIVLPFFTLSQNGYRTP
T T-----SCRDVSPNITG-----TDGCPSANEGSNSNNNNLVSHRIVLPFFTLSHNYNERP

       1810      1820      1830      1840      1850      1860
R QDAQELMNQNIKQYLHQLRSMLDTTIYCRFTGIVSSMHYKLDEVLLEYNSFDSAITLAEG
E SSNESQTQDEISKYLRQLRSVIDTTVYCRFTGIVSSMHYKLDEVLWEIENFKSAVTLAEG
S EPSPEESRSNIKGLLQHLRTMVDTTIYCRFTGIVSSMHYKLDEVLWEYNKFESAVTLAEG
B SVKKSEYVTEITKLIRQLKAIPDTTVYCRFTGVVSSMHYKLDEVLWEFDSFKTAVTLAEG
T SIRKSEGTTEIVRLTRQLRAIPDTTIYCRFTGIVSSMHYKLDEVLWEFDNFKSAITLAEG

       1870      1880      1890      1900      1910      1920
R EGSGALLLLQKYSTRLLFLNTLATEHSIESEVVSGFSTPRMLLPIMQKVHEGQVTVILNN
E EGAGALLLIQKYQVKTLFFNTLATESSIESEIVSGMTTPRMLLPVMSKFHNDQIEIILNN
S EGSGALLLIQKYGVKKLFLNTLATEHSIESEVISGYTTPRMLLSIMPKTHRGELEVILNN
B EGSGALLLLQKYKVRTIFFNTLATEHSIEAEIVSGTTTPRMLLPVMAKLHDDQINVILNN
T EGSGALLLLQKYKVETLFFNTLATEHSIEAEIISGITTPRMLLPIMSRFHGGQIKVTLNN

       1930      1940      1950      1960      1970      1980
R SASQITDITSSMWLS-NQKYNLPCQVEIIMMDAETTENLNRSQLYRAVYNLILDHIDPQY
E SASQITDITNPTWFK-DQRARLPRQVEVITMDAETTENINRSKLYEAVHKLILHHVDPSV
S SASQITDITHRDWFS-NQKNRIPNDADIITMDAETTENLDRSRLYEAVYTIICNHINPKT
B SASQVTDITNPAWFT-DQKSRIPTQVEIMTMDAETTENINRSKLYEAIQQLIVSHIDTRV
T SASQITDITNPSWLA-DQKSRIPKQVEIITMDAETTENINRSKLYEAVQQLIVSHIDPNA

       1990      2000      2010      2020      2030      2040
R LKVVVLKVFLSDIEGILWINDYLAPLFGAGYLIKPITSSARSSEWYLCLSNLISTNRRSA
E LKAVVLKVFLSDTEGMLWLNDNLAPFFATGYLIKPITSSARSSEWYLCLTNFLSTTRKMP
S LKVVILKVFLSDLDGMCWINNYLAPMFGSGYLIKPITSSAKSSEWYLCLSNLLSTLRTTQ
B LKIVIIKVFLSDIDGLLWLNDHLAPLFGSGYLIKPITSSPKSSEWYLCLSNFLSASRRRP
T LKVVVLKVFLSDIDGILWLNDNLTPLFGLGYLIKPITSSPKSSEWYLCLSNLLSTSRRLP

       2050      2060      2070      2080      2090      2100
R HQTHKACLGVIRDALQAQVQRGVYWLSHIAQYATKNLHCEYIGLGFPSLEKVLYHRYNLV
E HQNHLSCKQVILTALQLQIQRSPYWLSHLTQYADCDLHLSYIRLGFPSLEKVLYHRYNLV
S HQTQANCLHVVQCALQQQVQRGSYWLSHLTKYTTSRLHNSYIAFGFPSLEKVLYHRYNLV
B HQGHATCMQVIQTALRLQVQRSSYWLSHLVQYADINLHLSYVNLGFPSLEKVLYHRYNLV
T HQSHTTCMHVIQTALQLQIQRSSYWLSHLVQYANHNLHLDYINLGFPSLERVLYHRYNLV
```

```
                 2110                2120                2130                2140                2150                2160
R DTGLGPLSSVIRHLTNLQAEIRDLVLDYNLMRESRTQTYHFIKTAKGRITKLVNDFLKFS
E DSKRGPLVSVTQHLAHLRAEIRELTNDYNQQRQSRTQTYHFIRTAKGRITKLVNDYLKFF
S DSRNGPLVSITRHLALLQTEIRELVTDYNQLRQSRTQTYHFIKTSKGRITKLVNDYLRFE
B DSRKGPLVSILYHLTHLQAEIRELVCDYNQQRQSRTQTYHFIKTTKGRITKLVNDYLKFY
T DSQKGPLTSIVQHLAHLQTEIRELVNDYNQQRQSRTQTYHFIKTIKGRITKLVNDYLKFF

                 2170                2180                2190                2200                2210                2220
R LIVQALKNNSSWYTELKKLPEVINVCNRFYHTHNCECQEKFFVQTLYLQRLRDAEIKLIE
E LIVQALKHNGTWQAEFKKLPELISVCNRFYHIRDCNCEERFLVQTLYLHRMQDSEVKLIE
S LVIRALKNNSTWHHELYLLPELIGVCHRFNHTRNCTCSERFLVQTLYLHRMSDAEIKLMD
B LVVQALKHNCLWQEELRTLPDLINVCNRFYHIRDCSCEDRFLIQTLYLTRMQDSEVKLME
T LIIQALKHNCTWQEELRALPDLISVCTRFYHTRNCSCENRFLVQTLYLSRMQDSEIKLID

                 2230
R RLTGLMRFYPEGLIYSNHT
E RLTGLLSLFPDGLYRFD--
S RLTSLVNMFPEGFRSSSV-
B RLTGFLGLYPNGINT----
T RLTGLLSLCPNGFFR----
```

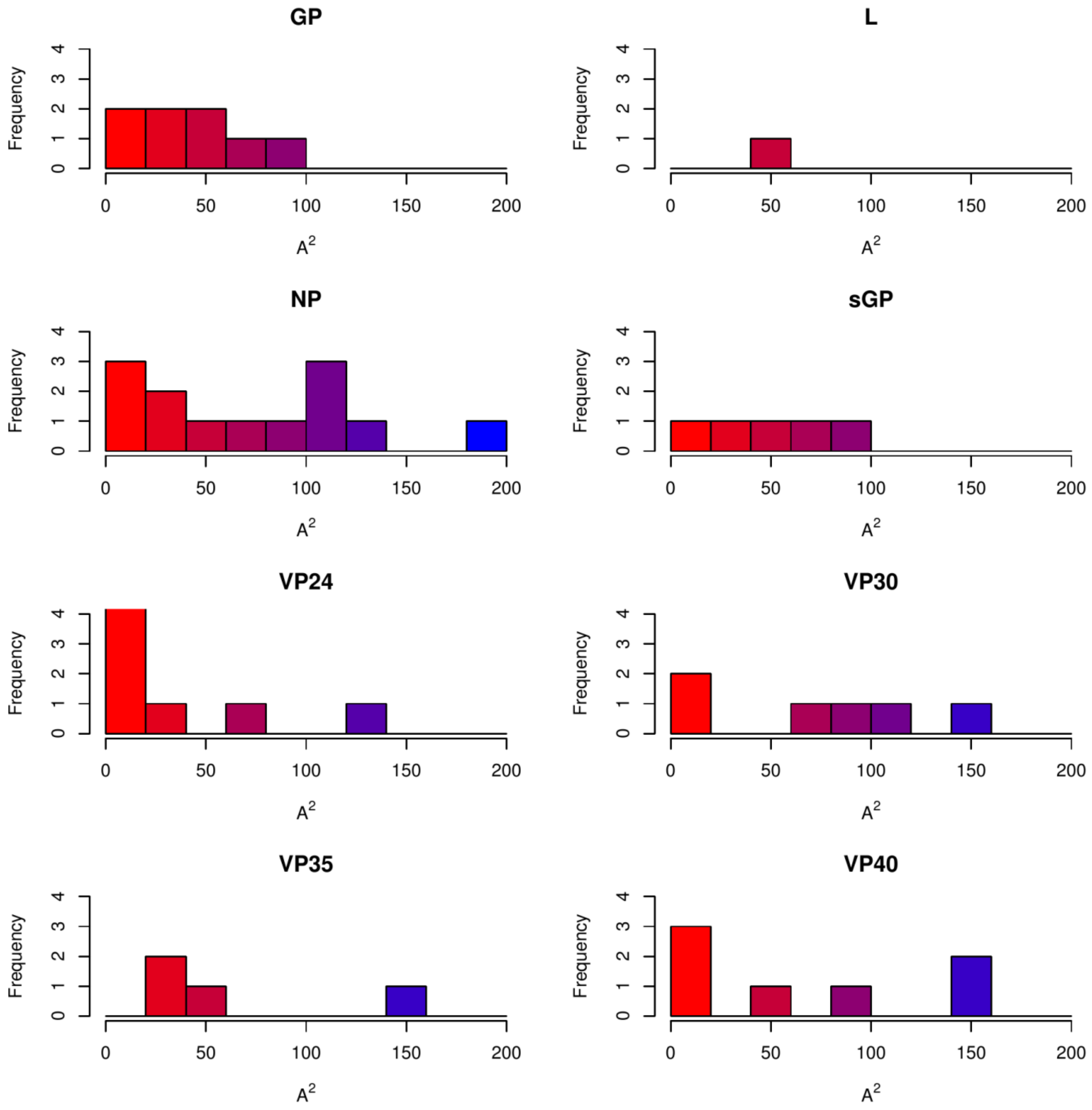

Supplementary Figure 3. Solvent Accessible surface area for Ebolavirus SDPs. Histograms showing the Solvent Accessible surface area in square ångstroms of SDPs. Values are calculated for the Ebola virus structure and residues.

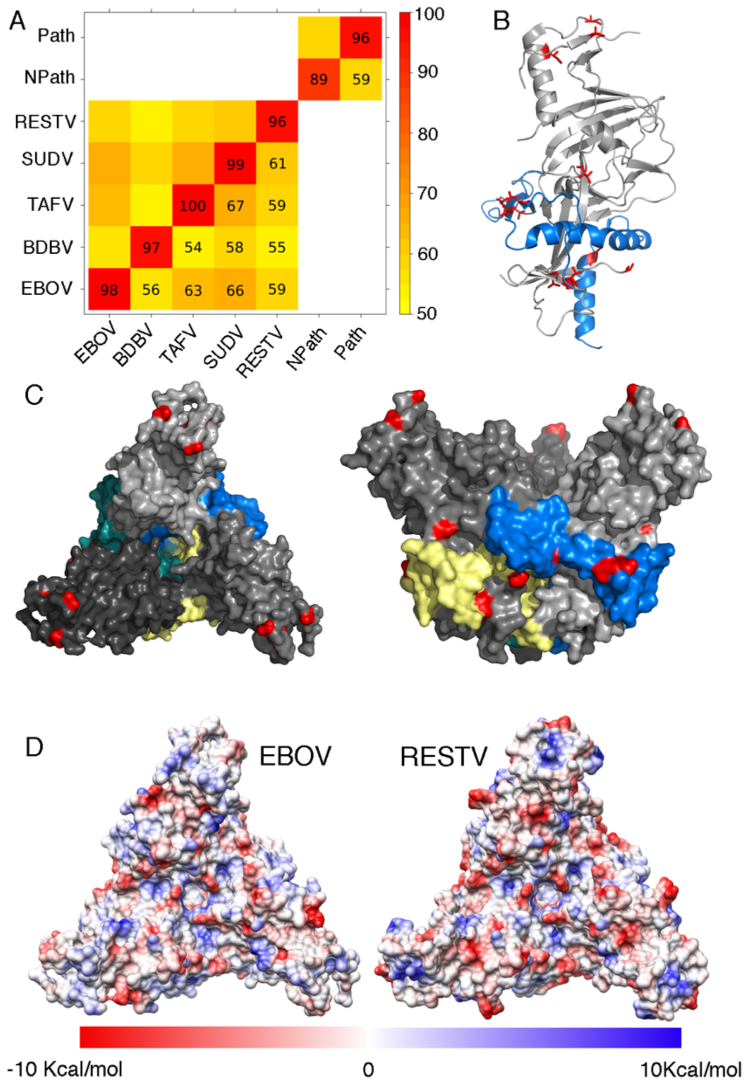

Supplementary Figure 4. GP SDPs. A) Heatmap of intra- and inter-species GP sequence identity (EBOV, Ebola virus; BDBV, Bundibugyo virus; SUDV, Sudan virus; TAFV, Taï Forest virus; RESTV, Reston virus). B) Monomeric representation of GP with GP1 (grey) and GP2 (blue). D) EBOV GP trimer (PDB code: 3CSY) with SDPs colored red. The three GP1 chains are colored grey. The three GP2 chains are colored blue, green and yellow. C) Electrostatics surfaces for the EBOV structure (3CSY) and a model of a RESTV GP trimer based on 3CSY.

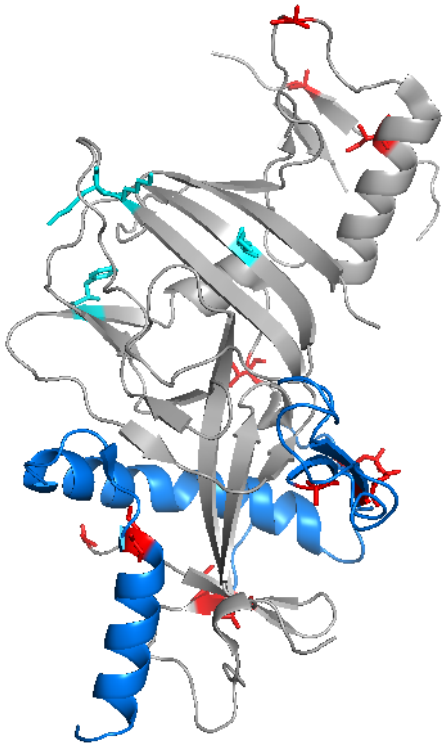

Supplementary Figure 5. GP SDPs are located outside the putative NPC1 binding site. GP SDPS are shown in red. The putative NPC1 binding site is shown in cyan.

Supplementary Figure 6. SDP prediction with subsampling of Ebolavirus sequences. The two groups of sequences ‘human pathogenic’ and Reston (‘non human pathogenic’) were sampled and SDP predictions made (see materials and methods). The boxplots show the distributions of the number of SDPs predicted in the simulations where A) only human pathogenic sequences were sampled, B) only Reston sequences were sampled and C) both sets were sampled. Sampling was performed for samples consisting of between 10%-90% of sequences (x axis). Red lines indicate the number of SDPs predicted in the full dataset without sampling. Note the scale of the Y-axis varies between each plot.

A. **Human pathogenic sequence sampled.**

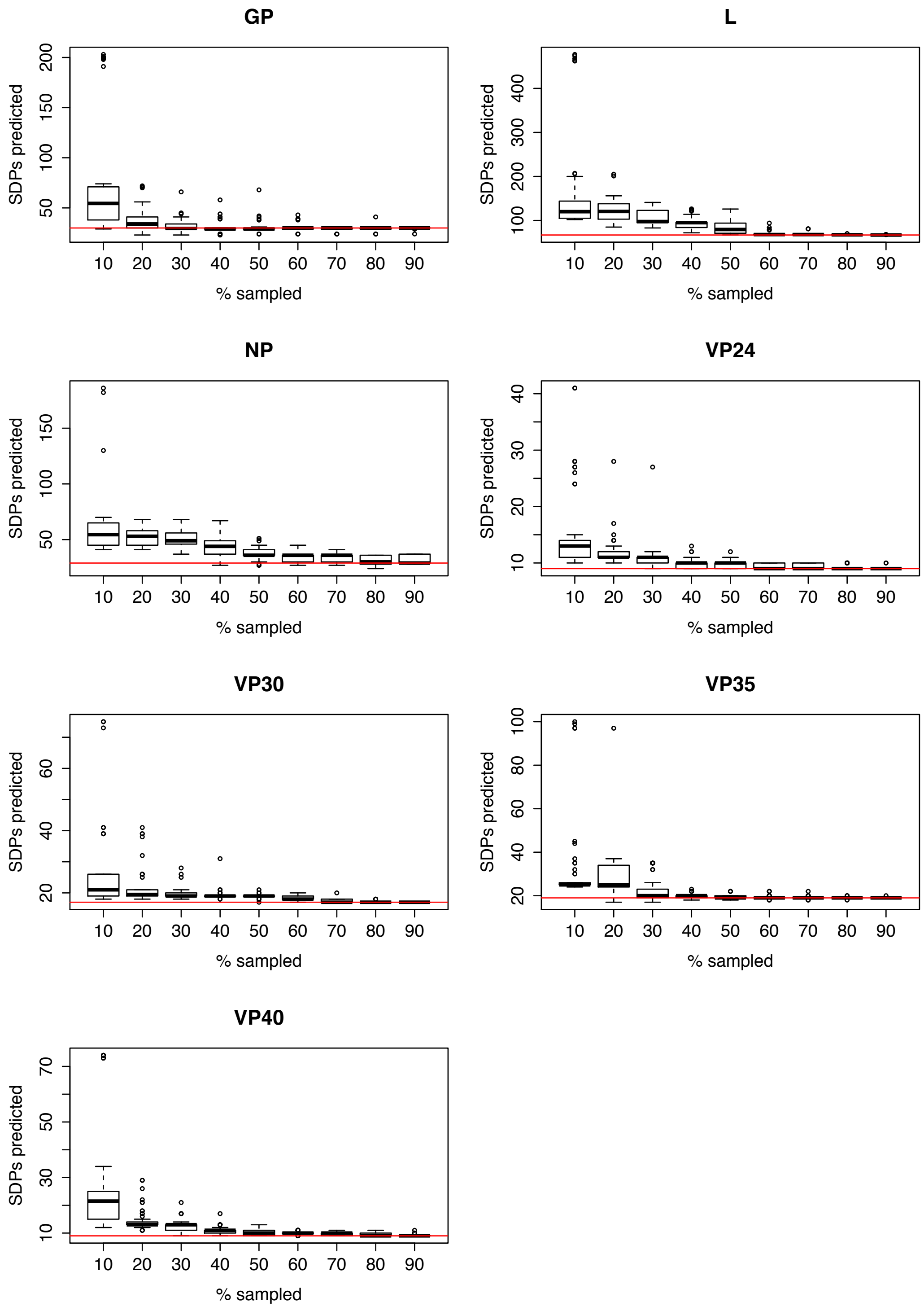

B. **Reston Sequences Sampled**

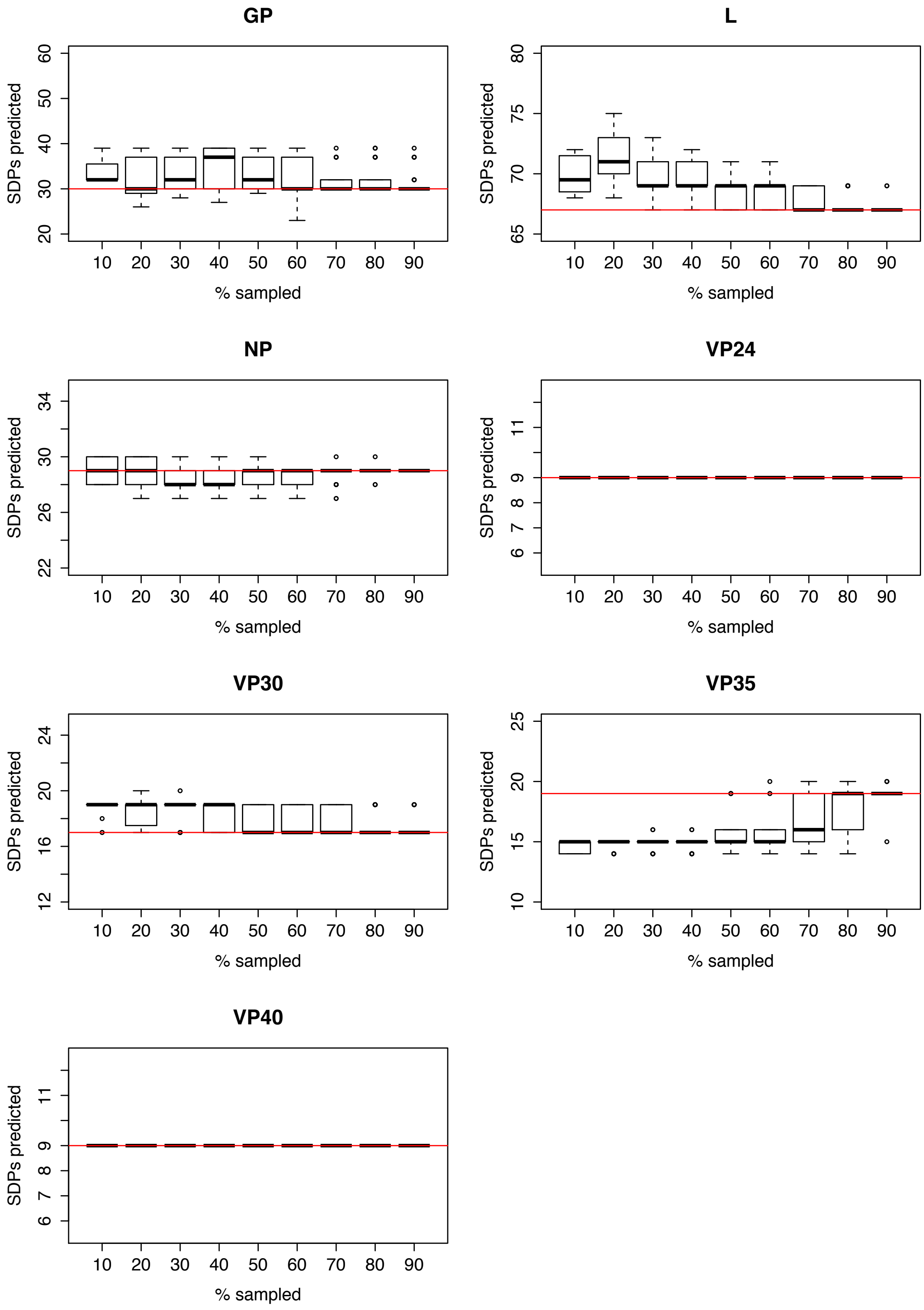

C. **Both groups sampled**

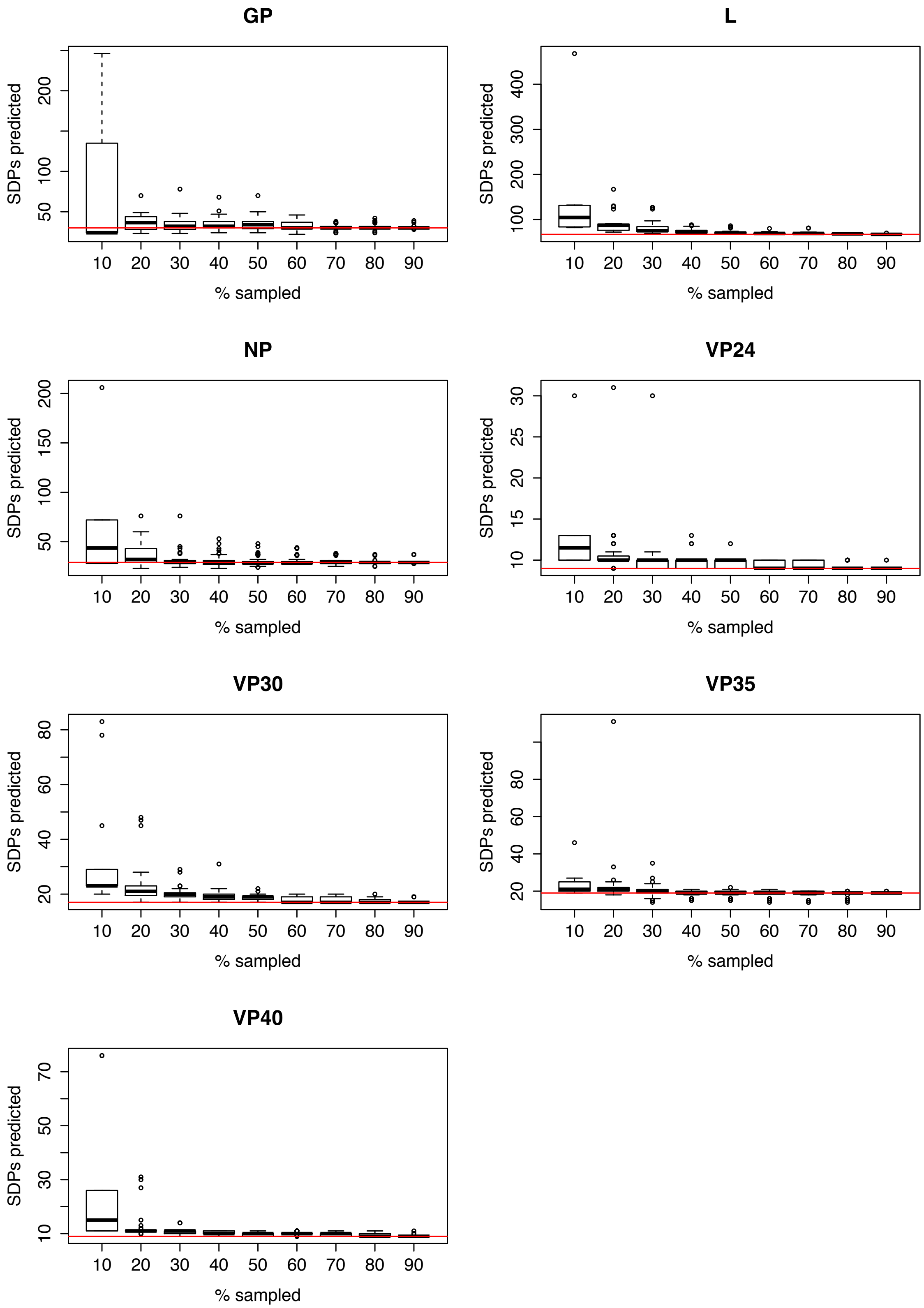

Supplementary Figure 7. Change in SDP prediction with subsampling of Ebolavirus sequences. The two groups of sequences ‘human pathogenic’ and and Reston (‘non human pathogenic’) were sampled and SDP predictions made (see materials and methods). The boxplots show the number of SDPs predicted in each sampling that are also in the full dataset (red) and new SDPs that are predicted only in subsamples (blue). The black horizontal line indicates the number of SDPs predicted using the full dataset. Subsampling performed for A) only human pathogenic sequences were sampled, B) only Reston sequences were sampled and c) both sets were sampled.

A. **Human pathogenic sequence sampled.**

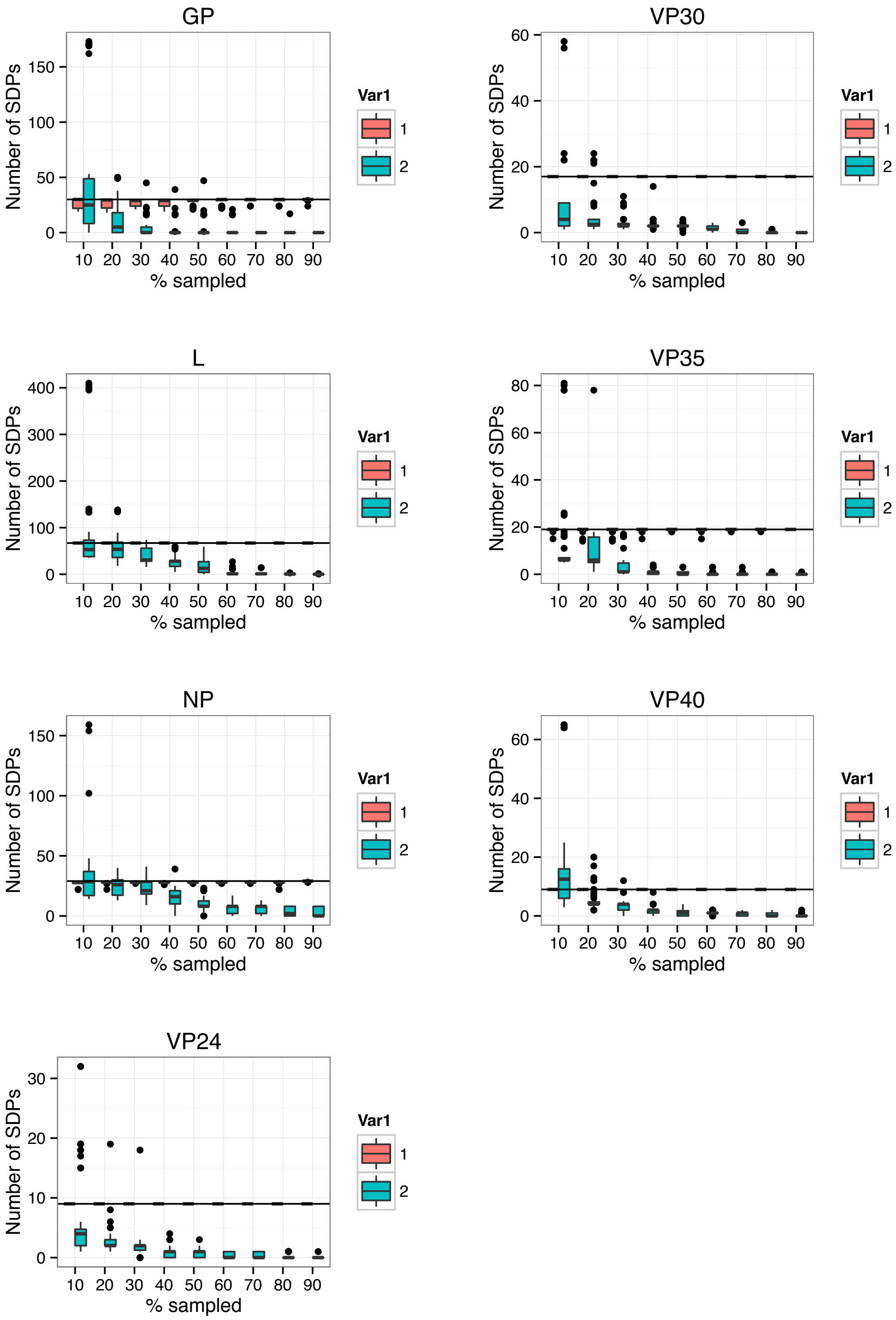

B. Reston Sequences Sampled

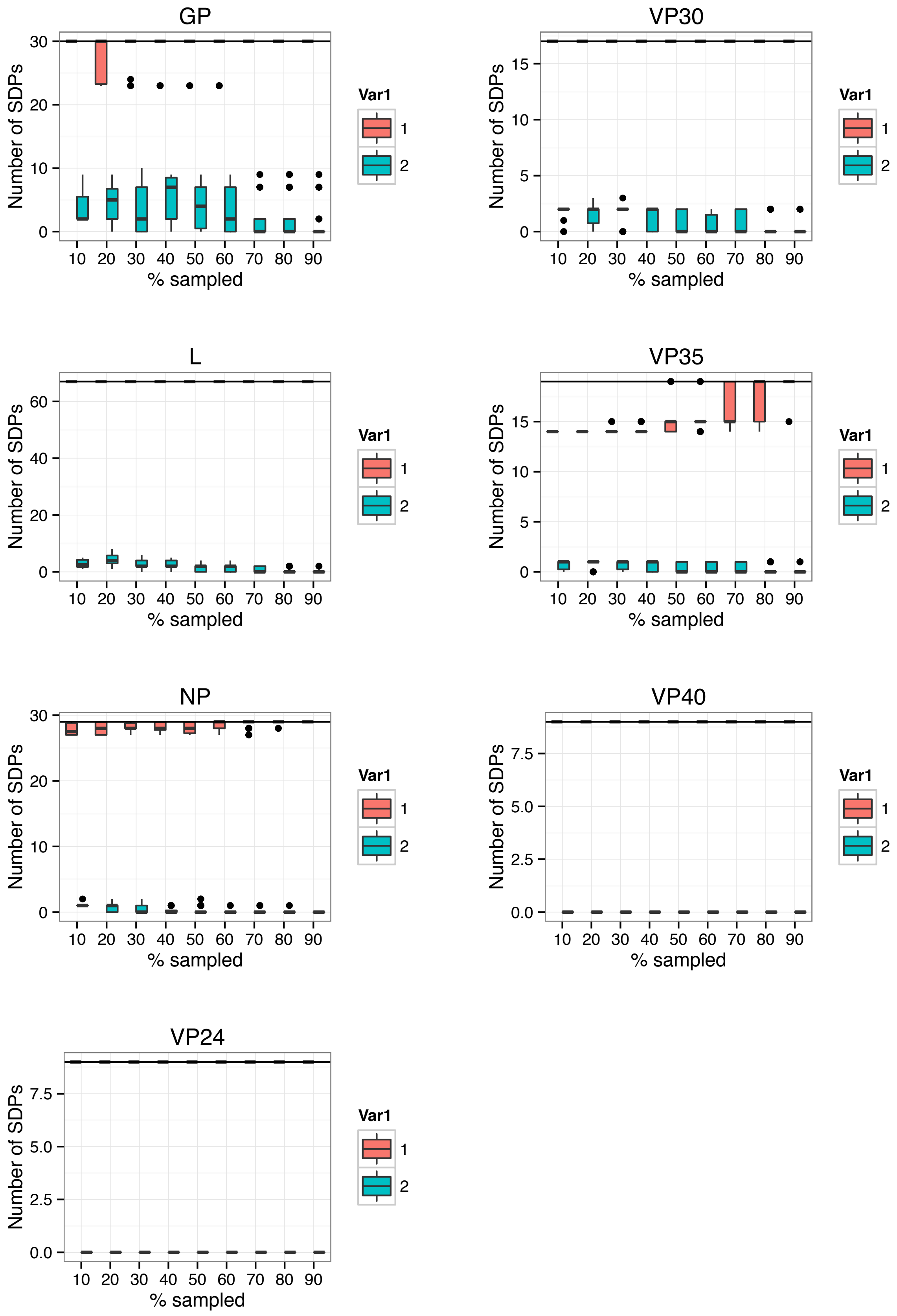

C. Both groups sampled

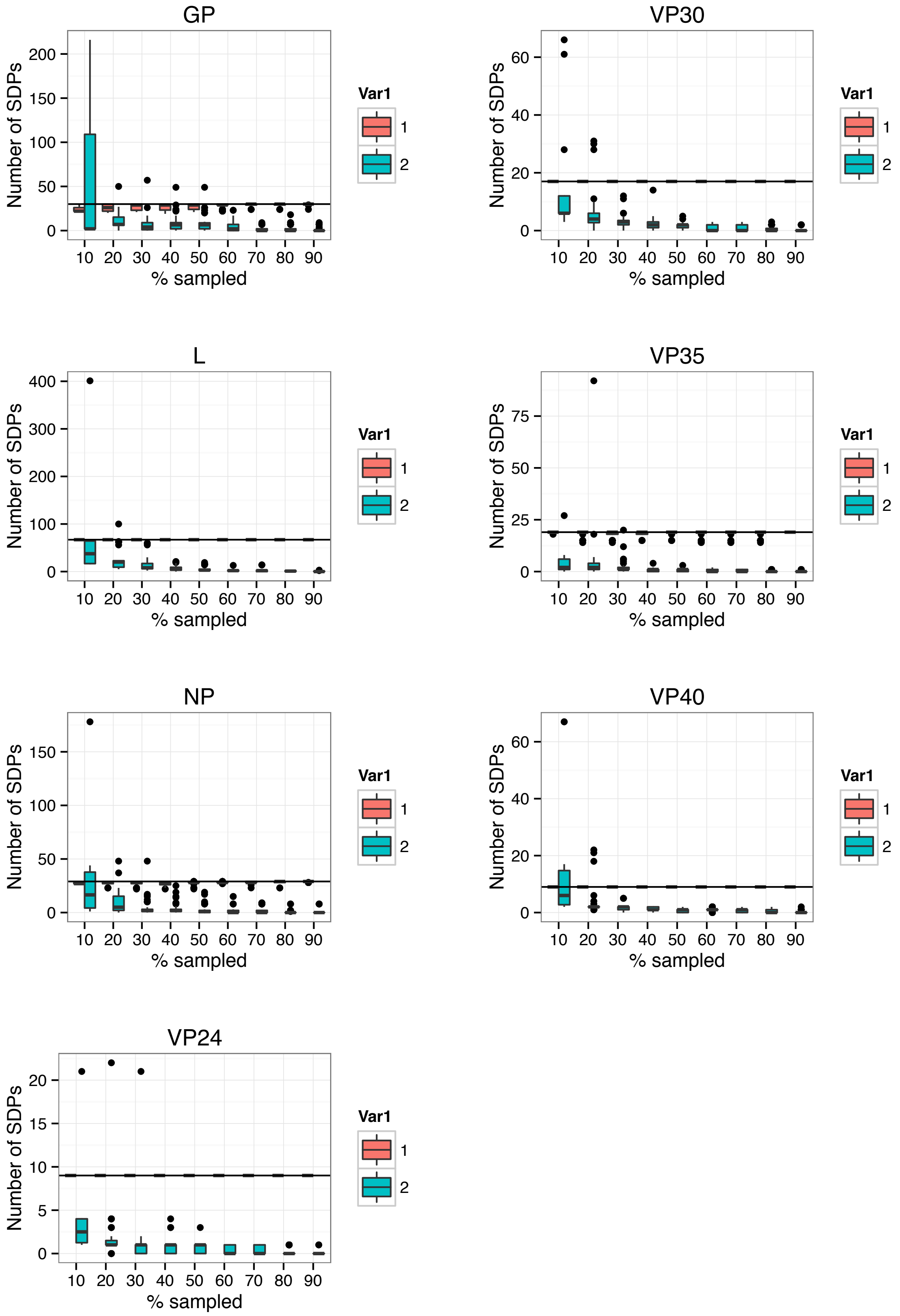

Supplementary Figure 8. Analysis of completely conserved SDP with subsampling of Ebolavirus sequences. The two groups of sequences 'human pathogenic' and and Reston ('non human pathogenic') were sampled and SDP predictions made (see materials and methods). The boxplots show the number of SDPs predicted in each sampling that are are completely conserved (red) and not completely conserved (blue). The red horizontal line indicates the number of completely conserved SDPs present in the full dataset and the blue line represents the equivalent for SDPs that are not completely conserved. Subsampling performed for A) only human pathogenic sequences were sampled, B) only Reston sequences were sampled and c) both sets were sampled.

A. **Human pathogenic sequence sampled.**

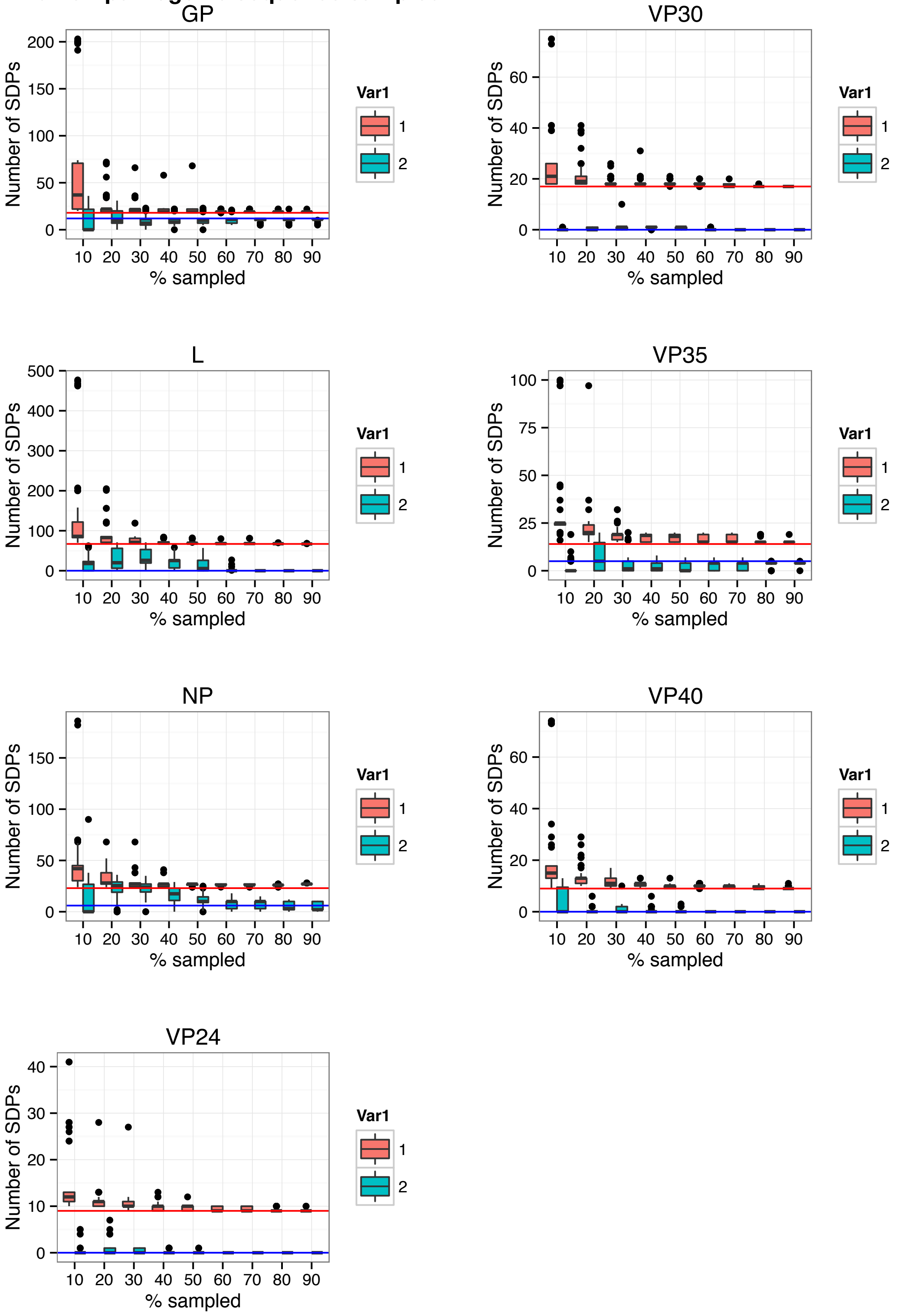

B. Reston Sequences Sampled

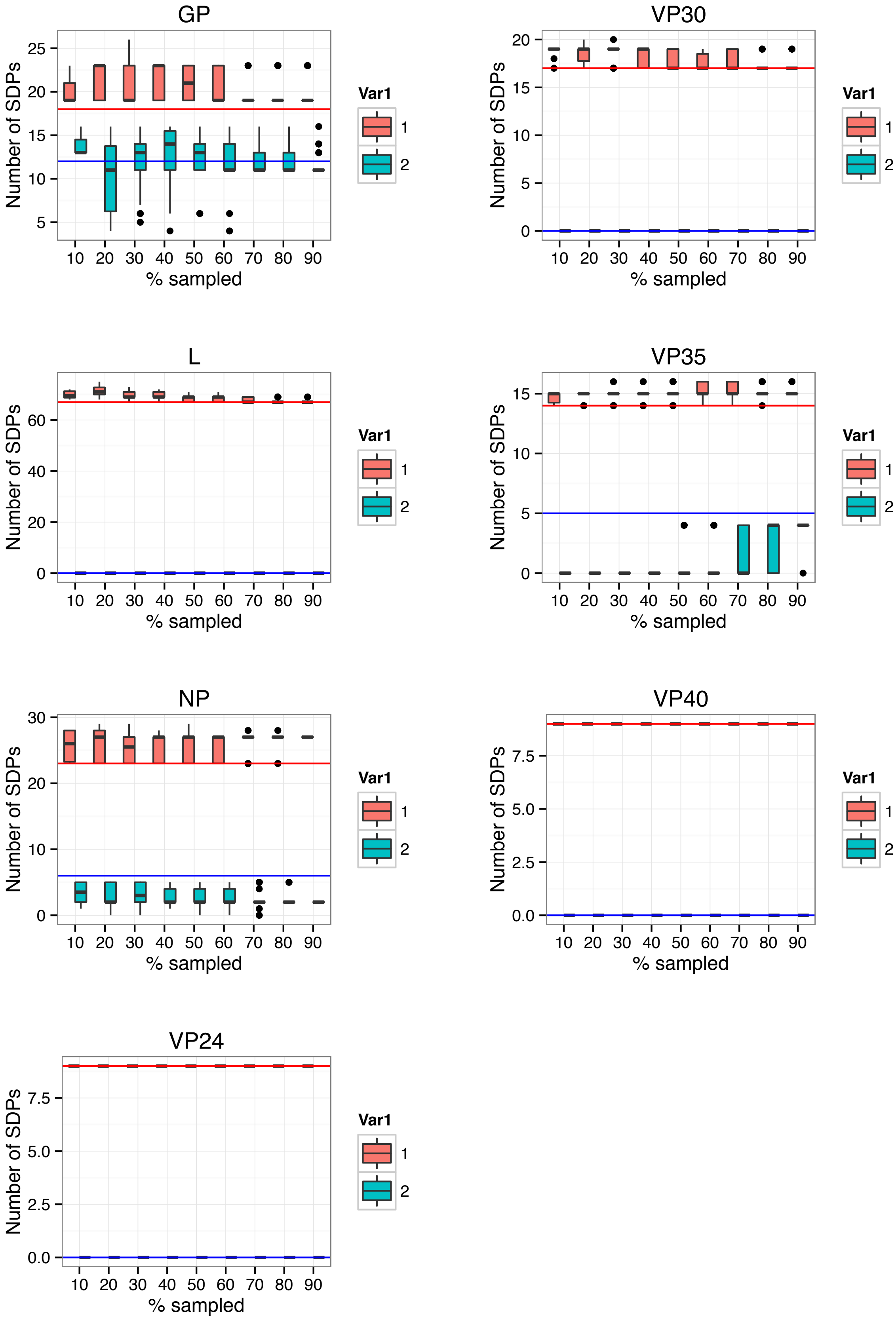

C. Both groups sampled

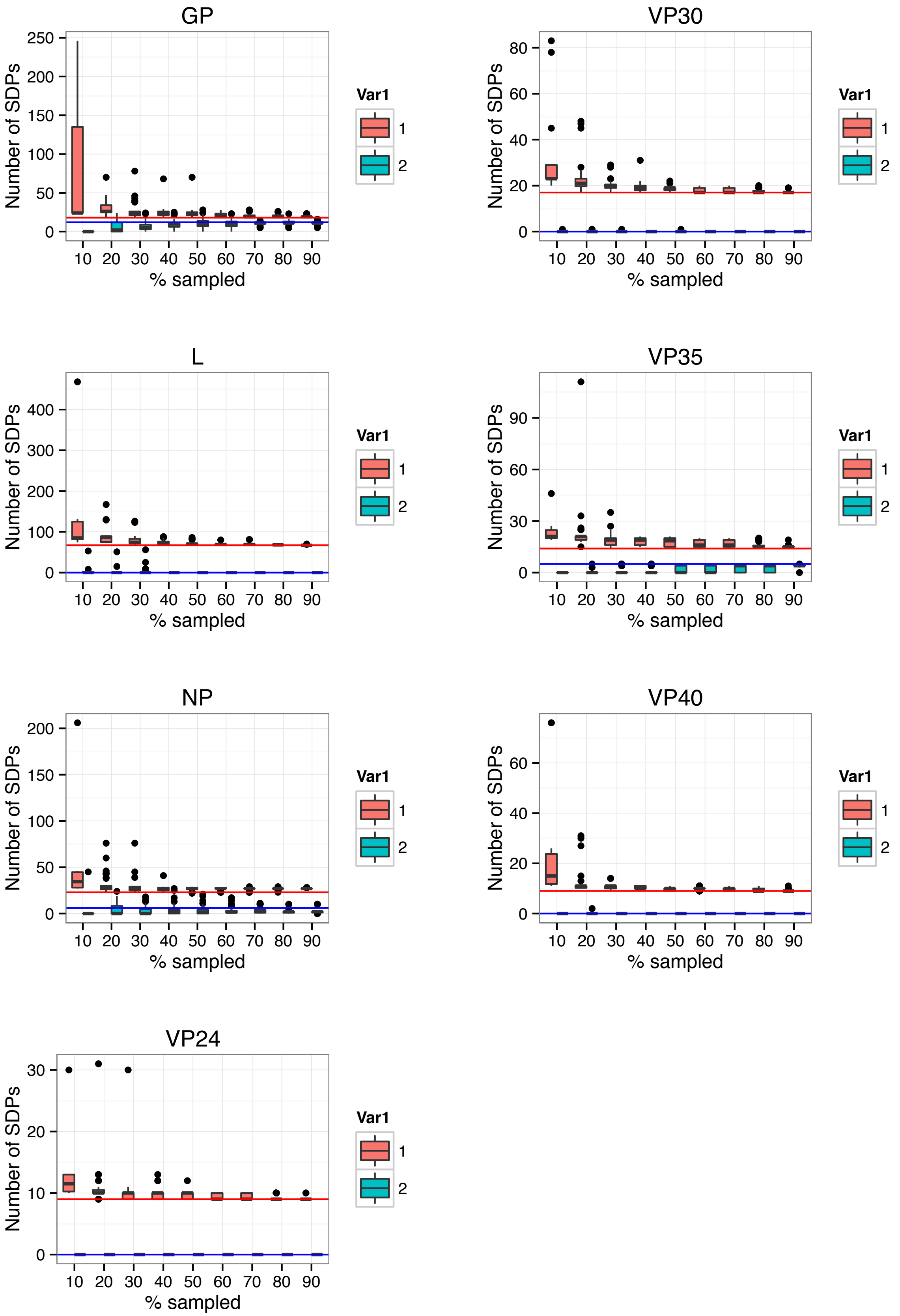

Supplementary Tables

	completely conserved positions	Number of Positions with variation	% of positions with variation
All species	2597	4555	64%
Ebola virus	4287	2865	40%
Sudan virus	4363	2789	38%
Bundibugyo virus	4426	2726	38%
Tai forest virus	4480	2672	37%
Reston virus	4466	2686	38%

Supplementary Table 1. Variation within the Ebolavirus genomes. The number of positions in the Ebolavirus protein multiple sequence alignments that are completely conserved and those that have variation are shown.

Alignment position	RESTV	EBOV	BDBV	SUDV	TAFV	BLOSUM 62 score	SASA (Å^2)	mCSM (Δ Δ G, Kcal/mol)	S3det Rank
17	M17	L17	L17	L17	L17	2	70	-0.444 (destabilising)	1
22	I22	V22	V22	V22	V22	3	0	-0.916 (destabilising)	1
31	I31	V31	V31	V31	V31	3	17	-0.193 (destabilising)	1
131	S131	T131	T131	T131	T131	1	36	-1.394 (destabilising)	1
132	T132	N132	N132	N132	N132	1	9	-1.121 (destabilising)	1
136	L136	M136	M136	M136	M136	2	2	-1.7 (destabilising)	1
139	R139	Q139	Q139	Q139	Q139	1	132	0.05 (stabilising)	1
226	A226	T226	T226	T226	T226	0	2	-0.935 (destabilising)	1
248	L248	S248	S248	S248	S248	-2	-		1

Supplementary Table 2. VP24 SDPs. The position in the multiple sequence alignment, the amino acid position, and amino acid present in each of the species is shown. The BLOSUM62 score represents how frequently such amino acid changes are observed in nature. SASA is the solvent accessible surface area, which is only available for SDPs that could be mapped to protein structure. SASA was calculated using the protein structure with PDB code 4M0Q. RESTV, Reston virus; EBOV, Ebola virus; B, Bundibugyo virus; SUDV, Sudan virus; TAFV, Taï Forest virus. The s3det column shows the ranking of the SDPs by s3det.

Alignment position	RESTV	EBOV	BDBV	SUDV	TAFV	BLOSUM 62 score	SASA ($Å^2$)	mCSM ($\Delta\Delta$G, Kcal/mol)	S3det rank
53	N53	T52	T52	T52	T52	0	-		1
54	L54	V53	V53	V53	V53	1	-		1
64	I64	T63	T63	T63	T63	-1	-		1
94	D94	E93	E93	E93	E93	2	-		1
97	N97	T96	T96	T96	T96	0	-		1
99	H99	R98	R98	R98	R98	0	-		1
108	R108	K107	K107	K107	K107	2	-		1
112	I112	S111	S111	S111	S111	-2	-		1
117	S117	K116	K116	K116	K116	0	-		1
121	S121	A120	A120	A120	A120	1	-		1
151	I151	T150	T150	T150	T150	-1	7	0.455 (stabilising)	1
158	R158	Q157	Q157	Q157	Q157	1	70	-0.493 (destabilising)	1
160	L160	I159	I159	I159	I159	2	6	-0.859 (destabilising)	1
197	H197	R196	R196	R196	R196	0	83	-1.291 (destabilising)	1
206	D206	E205	E205	E205	E205	-2	148	-0.373 (destabilising)	1
263	A263	R262	R262	R262	R262	-1	106	-0.969 (destabilising)	1
269	Q269	S268	S268	S268	S268	0	-		1

Supplementary Table 3. VP30 SDPs. The position in the multiple sequence alignment, the amino acid position, and amino acid present in each of the species is shown. The BLOSUM62 score represents how frequently such amino acid changes are observed in nature. SASA is the solvent accessible surface area, which is only available for SDPs that could be mapped to protein structure. SASA was calculated using the protein structure with PDB code 2I8B. RESTV, Reston virus; EBOV, Ebola virus; B, Bundibugyo virus; SUDV, Sudan virus; TAFV, Taï Forest virus. The s3det column shows the ranking of the SDPs by s3det.

Alignment position	REST V	EBOV	BDB V	SUDV	TAFV	BLOSUM 62 SCORE	SAS A (Å^2)	mCS M (Δ Δ G, Kcal/ mol)	S3det rank
27	T15	S26	S26	S26	S26	1	-		1
49	D37	E48	E48	E48	E48	2	-		1
77	E65	D76	D76	D76	D76	2	-		2
86	K74	E85	E85	E85	D86	1	-		3
93	M81	S92	S92	S92	S92	-1	-		1
98	T86	V97	V97	V97	I98	0	-		3
102	N90	T101	T101	T101	A102	0	-		3
107	A95	S106	S106	S106	S106	1	-		1
122	I110	V121	V121	V121	M122	3	-		3
155	S143	A154	A154	A154	A154	1	-		1
160	V148	T159	T159	T159	T159	0	-		1
161	D149	E160	E160	E160	E160	2	-		1
168	K156	G167	G167	G167	G167	-2	-		1
175	A163	S174	S174	S174	S174	1	-		1
182	L170	I181	I181	I181	I181	2	-		2
270	D258	E269	E269	E269	E269	2	144	-0.039 (destabilising)	1
291	V279	A290	A290	A290	A290	0	23	-0.756 (destabilising)	1
315	A303	V314	V314	V314	V314	0	49	-1.47 (destabilising)	1
330	K318	Q329	Q329	Q329	Q329	1	32	-0.513 (destabilising)	1

Supplementary Table 4. VP35 SDPs. The position in the multiple sequence alignment, the amino acid position, and amino acid present in each of the species is shown. The BLOSUM62 score represents how frequently such amino acid changes are observed in nature. SASA is the solvent accessible surface area, which is only available for SDPs that could be mapped to protein structure. SASA was calculated using the protein structure with PDB code 4IBB. RESTV, Reston virus; EBOV, Ebola virus; B, Bundibugyo virus; SUDV, Sudan virus; TAFV, Taï Forest virus. The s3det rank column shows the ranking of the SDPs by s3det. The s3det column shows the ranking of the SDPs by s3det.

Alignment position	RESTV	EBOV	BDBV	SUDV	TAFV	BLOSUM 62 SCORE	SASA (Å^2)	mCSM (Δ Δ G, Kcal/mol)	S3det rank
46	V46	T46	T46	T46	T46	0	83	-0.31 (destabilising)	1
85	T85	P85	P85	P85	P85	-1	142	-0.626 (destabilising)	1
122	V122	I122	I122	I122	I122	3	-		1
201	N201	G201	G201	G201	G201	0	53	-0.482 (destabilising)	1
209	L209	F209	F209	F209	F209	0	15	-1.219 (destabilising)	1
245	P245	Q245	Q245	Q245	Q245	-1	160	0.059 (stabilising)	1
269	Q269	H269	H269	H269	H269	0	-		1
293	V293	I293	I293	I293	I293	3	14	-1.411 (destabilising)	1
325	D325	E325	E325	E325	E325	2	-		1

Supplementary Table 5. VP40 SDPs. The position in the multiple sequence alignment, the amino acid position, and amino acid present in each of the species is shown. The BLOSUM62 score represents how frequently such amino acid changes are observed in nature. SASA is the solvent accessible surface area, which is only available for SDPs that could be mapped to protein structure. SASA was calculated using the protein structure with PDB code 1ES6. RESTV, Reston virus; EBOV, Ebola virus; B, Bundibugyo virus; SUDV, Sudan virus; TAFV, Taï Forest virus. The s3det column shows the ranking of the SDPs by s3det.

Alignment position	RESTV	EBOV	BDBV	SUDV	TAFV	BLOSUM 62 SCORE	SASA (Å^2)	mCSM (ΔΔ G, Kcal/mol)	S3det rank
4	G4	R4	R4	R4	R4	-2			1
16	D16	E16	E16	E16	G16	2			2
30	T30	S30	S30	S30	S30	1			1
39	K39	R39	R39	R39	R39	2	188	-0.161 (destabilising)	1
42	S42	P42/ Q42	P42	P42	Q42	-1	103	-2.173 (destabilising)	3
56	V56	I56	I56	I56	I56	3	0	-0.8 (destabilising)	1
64	I64	V64	V64	V64	V64	3	7	-0.135 (destabilising)	1
105	K105	R105	R105	R105	R105	2	112	-0.63 (destabilising)	1
137	L137	M137	M137	M137	M137	2	37	-0.649 (destabilising)	1
212	Y212	F212	F212	F212	F212	3	0	-0.692 (destabilising)	1
274	R274	K274	K274	K274	K274	2	92	-0.548 (destabilising)	1
279	A279	S279	S279	S279	S279	1	60	-0.822 (destabilising)	1
374	R374	K374	K374	K374	K374	2	103	-0.836 (destabilising)	1
416	N416	K416	K416	K416	K416	0			1
421	Q421	Y421	Y421	Y421	Y421	-1			1
426	E426	D426	D426	D426	D426	2			1
435	N435	D435	D435	D435	D435	1			1
443	E443	D443	D443	D443	D443	2			1
453	I453	T453	T453	T453	T453	-1			1

492	E492	D492	D492	D492	D492	2			1
497	A497	P497	P497	P497	P497	-1			2
535	(-)	P526	P526	P526	P526				1
572	S563	T563	T563	T563	T563	1			1
574	V565	I565	I565	I565	I565	3			1
611	T602	P602	P602	P602	N602	-1			4
651	Q641	N641	N641	N641	K641	0			2
715	R705	A705	A705	A705	A705	-1	24	-1.037 (destabilising)	1
726	N716	D716	D716	D716	D716	1	123	0.141 (stabilising)	1
727	N717	G717	G717	G717	G717	0	75	-0.461 (destabilising)	2

Supplementary Table 6. NP SDPs. The position in the multiple sequence alignment, the amino acid position, and amino acid present in each of the species is shown. The BLOSUM62 score represents how frequently such amino acid changes are observed in nature. SASA is the solvent accessible surface area, which is only available for SDPs that could be mapped to protein structure. SASA was calculated using the protein structure with PDB code 4QB0 for the C terminal and 4YPI for the N terminal regions. RESTV, Reston virus; EBOV, Ebola virus; B, Bundibugyo virus; SUDV, Sudan virus; TAFV, Taï Forest virus. The s3det rank column shows the ranking of the SDPs by s3det. The s3det column shows the ranking of the SDPs by s3det.

Alignment position	RESTV	EBOV	BDBV	SUDV	TAFV	BLOSUM 62 Score	SASA (Å^2)	mCSM (ΔΔ G, Kcal/mol)	S3det rank
2	G2	M1	M1	M1	M1	-3			1
3	S3	G2	V2	E2/G2	G2	0			8
32	I32	F31	F31	F31	F31	0			1
38	I38	V37	V37	V37	V37	3	0	-0.828 (destabilising)	1
46	A46	V45	V45	V45	V45	0	30	-1.276 (destabilising)	1
76	I76	V75	V75	V75	V75	3	44	-0.295 (destabilising)	1
197	A197	S196	S196	S196	S196	1			1
208	D208	E207	T207	E207	T207	2			9
211	T211	S210	S210	S210	S210	1			1
261	L261	I260	I260	I260	I260	2	25	-0.95 (destabilising)	1
270	S270	T269	T269	T269	T269	1	99	-0.432 (destabilising)	1
308	H308	S308/ L307	S308	S308	S308	-1			2
326	G326	R325	V325	R325	V325	-2			9
355	L355	H354	R354	H354	Q354	-3			9
404	P401	Q403	N401	Q397	S401	-1			9
419	E412	S418	A409	S412	T409	0			9
461	P449	T448	S442	T448	T448	-1			7
497	Y517/ H517	H516	H516	H516	H516	2			6
519	K499	R498	R498	R498	R498	2			1
521	K501	R500	R500	R500	R500	2			1
535	D515	N514	N514	N514	N514	1	59	-1.142 (destabilising)	1
542	V522	Q521	Q521	Q521	L521	2	19	0.037 (stabilising)	6

568	V548	L547	I547	L547	I547	1	74	-1.258 (destabilising)	9
605	L585	I584	I584	I584	I584	2			1
628	S608	D607	D607	D607	D607	0			1
643	E623	K622	K622	K622	K622	1			1
659	H639	Q638	Q638	Q638	Q638	0			1
663	L643	D642	D642	D642	S642	-4			6
665	L645	W644	W644	W644	W644	-2			1
680	I660	T569	T569	T569	T569	-1			1

Supplementary Table 7. GP SDPs. The position in the multiple sequence alignment, the amino acid position, and amino acid present in each of the species is shown. The BLOSUM62 score represents how frequently such amino acid changes are observed in nature. SASA is the solvent accessible surface area, which is only available for SDPs that could be mapped to protein structure. SASA was calculated using the protein structure with PDB code 3CSY. RESTV, Reston virus; EBOV, Ebola virus; B, Bundibugyo virus; SUDV, Sudan virus; TAFV, Taï Forest virus. The s3det rank column shows the ranking of the SDPs by s3det. The s3det column shows the ranking of the SDPs by s3det.

Alignment position	RESTV	EBOV	BDBV	SUDV	TAFV	BLOSUM 62 SCORE	SASA ($Å^2$)	S3det rank
47	G2	M1	M1	M1	M1	-3		1
77	I32	F31	F31	F31	F31	0		1
83	I38	V37	V37	V37	V37	3	21	1
91	A46	V45	V45	V45	V45	0	84	1
121	I76	V75	V75	V75	V75	3	61	1
242	A197	S196	S196	S196	S196	1		1
256	T211	S210	S210	S210	S210	1		1
306	L261	I260	I260	I260	I260	2	20	1
315	S270	T269	T269	T269	T269	1	48	1

Supplementary Table 8. sGP SDPs. The position in the multiple sequence alignment, the amino acid position, and amino acid present in each of the species is shown. The BLOSUM62 score represents how frequently such amino acid changes are observed in nature. SASA is the solvent accessible surface area, which is only available for SDPs that could be mapped to protein structure. SASA was calculated using the Phyre2 structural model that used template structure 3s88I. RESTV, Reston virus; EBOV, Ebola virus; B, Bundibugyo virus; SUDV, Sudan virus; TAFV, Taï Forest virus. The s3det rank column shows the ranking of the SDPs by s3det. The s3det column shows the ranking of the SDPs by s3det.

Alignment position	RESTV	EBOV	BDBV	SUDV	TAFV	BLOSUM62 SCORE	SASA (Å^2)	mCSM (Δ Δ G, Kcal/mol)	S3det rank
67	T66	V66	V66	V66	V66	0			1
110	H109	Q109	Q109	Q109	Q109	0			1
137	L136	I136	I136	I136	I136	2			1
147	V146	L146	L146	L146	L146	1			1
222	S221	A221	A221	A221	A221	1			1
224	L223	Q223	Q223	Q223	Q223	-2			1
228	Q227	H227	H227	H227	H227	0			1
277	I276	L276	L276	L276	L276	2	42	-1.049 (destabilising)	1
284	V283	L283	L283	L283	L283	1			1
313	F312	Y312	Y312	Y312	Y312	3			1
327	S326	A326	A326	A326	A326	1			1
331	D330	T330	T330	T330	T330	-1			1
351	D350	E350	E350	E350	E350	2			1
362	S361	T361	T361	T361	T361	1			1
366	F365	L365	L365	L365	L365	0			1
380	I379	V379	V379	V379	V379	3			1
448	H447	Q447	Q447	Q447	Q447	0			1
451	S450	P450	P450	P450	P450	-1			1
466	N465	D465	D465	D465	D465	1			1
690	S689	E689	E689	E689	E689	0			1
848	A847	S847	S847	S847	S847	1			1
869	A868	S868	S868	S868	S868	1			1
897	Y896	F896	F896	F896	F896	3			1
926	F925	L925	L925	L925	L925	0			1
955	S954	A954	A954	A954	A954	1			1
996	T995	S995	S995	S995	S995	1			1
1025	N1024	T1024	T1024	T1024	T1024	0			1
1074	K1073	R1073	R1073	R1073	R1073	2			1
1120	S1119	A1119	A1119	A1119	A1119	1			1
1164	A1161	F1163	F1163	F1163	F1163	-2			1
1190	S1187	D1189	D1189	D1189	D1189	0			1
1215	S1212	A1214	A1214	A1214	A1214	1			1
1218	K1215	R1217	R1217	R1217	R1217	2			1
1238	E1235	D1237	D1237	D1237	D1237	2			1
1256	V1253	I1255	I1255	I1255	I1255	3			1
1355	K1532	R1534	R153	R1534	R153	2			1

			4		4				
1367	A1354	T1366	T1366	T1366	T1366	0			1
1396	T1393	S1395	S1395	S1395	S1395	1			1
1409	M1406	I1408	I1408	I1408	I1408	1			1
1415	L1412	I1414	I1414	I1414	I1414	2			1
1437	N1434	S1436	S1436	S1436	S1436	1			1
1462	Q1459	K1461	K1461	K1461	K1461	1			1
1474	C1471	S1473	S1473	S1473	S1473	-1			1
1489	Y1486	L1488	L1488	L1488	L1488	-1			1
1500	L1497	I1499	I1499	I1499	I1499	2			1
1507	A1504	S1506	S1506	S1506	S1506	1			1
1510	V1507	I1509	I1509	I1509	I1509	3			1
1539	S1536	A1535	A1535	A1535	A1535	1			1
1627	Y1624	L1624	L1624	L1624	L1624	-1			1
1631	S1628	C1628	C1628	C1628	C1628	-1			1
1786	I1760	V1762	V1762	V1762	V1762	3			1
1874	T1848	V1850	V1850	V1850	V1850	0			1
1897	S1871	T1873	T1873	T1873	T1873	1			1
1941	N1914	R1916	R1916	R1916	R1916	1			1
1966	R1939	E1941	E1941	E1941	E1941	0			1
2033	I2006	L2008	L2008	L2008	L2008	2			1
2069	I2042	L2044	L2044	L2044	L2044	2			1
2102	T2075	S2077	S2077	S2077	S2077	1			1
2123	D2096	E2098	E2098	E2098	E2098	2			1
2130	L2130	Q2105	Q2105	Q2105	Q2105	-2			1
2133	E2106	Q2108	Q2108	Q2108	Q2108	2			1
2156	F2129	Y2131	Y2131	Y2131	Y2131	3			1
2182	V2155	L2157	L2157	L2157	L2157	1			1
2193	N2171	R2168	R2168	R2168	R2168	0			1
2200	K2173	R2175	R217	R2175	R217	2			1

			5		5				
2202	F2175	L2177	L2177	L2177	L2177	0			1
2211	L2184	M2186	M2186	M2186	M2186	2			1

Supplementary Table 9. L SDPs. The position in the multiple sequence alignment, the amino acid position, and amino acid present in each of the species is shown. The BLOSUM62 score represents how frequently such amino acid changes are observed in nature. SASA is the solvent accessible surface area, which is only available for SDPs that could be mapped to protein structure. SASA was calculated using the Phyre2 structural model which used template 4n48A (“cap-specific mrna (“cap-specific mrna (nucleoside-2'-o-)-methyltransferase 1 protein in2 complex with capped rna fragment”). RESTV, Reston virus; EBOV, Ebola virus; B, Bundibugyo virus; SUDV, Sudan virus; TAFV, Taï Forest virus. The s3det rank column shows the ranking of the SDPs by s3det. The s3det column shows the ranking of the SDPs by s3det.

Protein	EBOV Res	RESTV Res	Mutation position	Mutation	Effect
GP	Q638	H	638	Q → V	No effect on release of soluble GP1,2delta.
GP	R498	K	498-501	RTRR → ATAA	No effect on cleavage between GP1 and GP2.
GP	D642	L	642	D → V	No effect on release of soluble GP1,2delta.
VP24	M136	L	134/136	F-A/M-A	Near complete loss of KPNA5 binding *
VP24	Q139	R	137-139	RTQ → AAA	Near complete loss of KPNA5 binding *

Supplementary Table 10. SDPs that coincide with known mutagenesis data. Functional data extracted from UniProt unless stated. Res, residue; EBOV, Ebola virus; RESTV, Reston virus
*Data from Bornholdt et al.,[35]

PROTEIN	SPECIES	OLIGOMERIC STATE	PDB/TEMPLATE	REGION IN SEQUENCE
GP	EBOV	Trimer of Heterodimers	3CSY (structure)	31-310<br>502-599
sGP	EBOV	Dimer	3s88I (model)	32-287
sGP	RESTV	Dimer	3s88I (model)	33-288
L	EBOV	Monomer	4n48A (model)	223-328
NP (C-terminal)	EBOV	Monomer	4QB0 (structure)	645-739
NP (N-terminal)	EBOV	Monomer	4YPI (structure)	39-384
VP24	EBOV	Heterodimer	4M0Q (structure)	10-231
VP24	EBOV	Heterodimer	4U2X (structure)	16-231
VP24	RESTV	Dimer	4D9O (structure)	10-231
VP30	EBOV	Dimer	2I8B (structure)	140-266
VP30	RESTV	Dimer	3V70 (structure)	142-272
VP35	EBOV	Heterodimer	4IBB (structure)	218-340
VP35	EBOV	Dimer of heterodimers	3L25 (structure)	209-340
VP35	RESTV	Dimer of heterodimers	3KS8 (structure)	208-329
VP40	EBOV	Monomer	1ES6 (structure)	44-321
VP40	EBOV	Dimer	4LDB (structure)	44-319
VP40	EBOV	Hexamer	4LDD (structure)	45-188
VP40	EBOV	Octamer	4LDM (structure)	69-188
VP40	RESTV	Monomer	1es6A (model)	44-321

Supplementary Table 11. Protein structures available for Ebolavirus Proteins. EBOV, Ebola virus; RESTV, Reston virus

Reston virus residue	Pathogenic consensus	Comments	Functional effect
I32	F31	Note- Ebola virus GP structure has R31 rather than F31. Surface residue close to interface with GP2 in the trimer. Unclear what functional effect may be if any.	unclear
I38	V37	Surface residue, appears to be a conservative change of amino acid that could be well tolerated	unlikely
A46	V45	Also a surface residue. Conservative change of hydrophobic amino acid that could be well accommodated.	unlikely
I76	V75	Surface residue, conservative change of amino acid . Change should be well accommodated	unlikely
L261	I260	One of three SDPs located in the glycan cap region of GP1. The glycan cap binds the host cell receptor(s) but is highly glycosylated so it is not clear if the amino acids directly contact the host cell. Surface residue in a cavity. It is part packed quite tightly with residue F234, V236, T240 but should be possible to accommodate change to Leu in Reston virus. Could there be a role with the three SDPs combined in this region.	possible*
S270	T269	Located at the top of the structure, is a surface residue (with side chain pointing to the solvent) representing a conservative amino acid change. Again could it have a role in conjunction with the 2 other SDPs in this region?	possible*
H308	S308/ L307	Also located in the glycan cap and also a surface residue. Present in loop so unlikely to alter structure but could have a functional role, and alters charge on the protein surface.	possible*
D515	N514	Surface residue, results in loss of negative charge in Reston virus GP. Located at the end of a beta sheet. Seems unlikely to have a structural effect. Possible combined effect with adjacent L547V?	unlikely
V522	Q521	Close to trimer interface (GP2-GP2) but directly within the interface. Not clear what effect this change would have on protein structure	unclear
V548	L547	Surface residue at end of a beta sheet. Appears to be minor change in amino acid. Possible combined effect with adjacent N514D?	unlikely
L585	I584	Largely buried amino acid. At the interface with GP1 (in the same GP monomer). EBOV I584 interacts with F572, not clear if this interaction would change in with Leu in Reston virus.	unlikely

Supplementary Table 12. Structural analysis of GP SDPs. Details of the structural analysis are included with an assessment of whether the amino acid change is likely to have an effect on the protein. Four categories are used for the effect column unlikely (the change seems unlikely to alter the structure/function), unclear (the change could be functional but there is limited evidence), possible (more confident that there is an effect than the unclear group) and probably (highly confident that the change will have a structural/functional effect).

Reston virus residue	Pathogenic consensus	Comments	Functional effect
K39	R39	R39 forms a H bond with D71. Change to K is likely to maintain this H bond.	unlikely
S42	P42/ Q42	Unusual to see Pro in a sheet. The amino acid is on the protein surface and it there is nothing to suggest that a change to Ser would alter the protein	unclear
V56	I56	I56 is largely buried and packed against other sidechains. While change to Val would reduce the size of the side chain, it seems likely that it would be accommodated within the structure. Also V64I is adjacent to this SDP.	unlikely
I64	V64	In a surface loop facing the helix containing I56V. Possible co-evolution with I56 – reduce size in one, matched with increased size in the other.	unlikely
K105	R105	The side chain guanidino group of R105 provides a hydrogen bond with the side chain of Q38 as well as with the local backbone NH of G103 to provide a stabilized region of the protein. Although the mutation R105K appears conservative and maintains the side chain positive charge, the ability to form multiple hydrogen bonds is reduced due to resonance stabilization in the guanidino group being lost in the transfer to the lysine side chain amino group. This has the potential to weaken interactions in this region.	possible
L137	M137	M137 is located at the end of helix and packs against an adjacent helix. The conservative change to L137 in Reston virus seems unlikely to have a significant effect on structure/function	unlikely
Y212	F212	A minor change in side chains. P212 is located in an alpha helix and the sidechain is largely buried. The change to Y212 in Reston virus is unlikely to have a significant effect on protein structure/function	unlikely
R274	K274	K274 is located in the VP35 binding site. K274 forms a hydrogen bond with VP35 D46 and a change to Arg should be able to maintain this interaction.	unlikely
A279	S279	S279 is located in an alpha helix on the protein surface. The change to A279 in Reston virus would introduce a hydrophobic amino acid on the protein surface that could have an effect on protein structure.	unclear
R374	K374	K374 is located in an alpha helix on the protein surface. It is not unlikely that the change to R374 in Reston virus will alter protein structure. It is a conservative change of side chain.	unlikely
R705	A705	A695 is located on the protein surface so the charge introduce by the change to R695 in Reston virus should be tolerated. Proximity of Reston virus R705 to E694 may result in a salt bridge that would reduce flexibility in Reston virus NP. There could different hydrodynamic volumes between the Reston virus and pathogenic NP proteins as well as in the pathogenic ebolaviruses exposing residues that remain buried in the Reston virus NP. The salt bridge could make RESTV more thermostable (and possibly more resistant to proteolysis and denaturants).	Possible
N716	D716	Present in a surface loop this change will change the charge properties. Should be considered with adjacent amino acid, which is also an SDP. Overall we see the removal of a negatively charged amino acid with two polar side chains.	unclear
N717	G717	Adjacent to D716N pSDP. The loss of Gly would change the turn from type1 to a type 2 turn. Also See comment above.	unclear

Supplementary Table 13. Structural analysis of NP SDPs. Details of the structural analysis are included

with an assessment of whether the amino acid change is likely to have an effect on the protein. Four categories are used for the effect column unlikely (the change seems unlikely to alter the structure/function), unclear (the change could be functional but there is limited evidence), possible (more confident that there is an effect than the unclear group) and probably (highly confident that the change will have a structural/functional effect).

Reston virus Residue	Pathogenic consensus	Comments	Functional effect
D258	E269	Present in dimer interface (only for one of the subunits as the dimer is asymmetric). Forms hydrogen bonds with R301, R311 and W313 (RESTV numbering). Distances between atoms are slightly different between the 2 species. W324 3.1A (2.8 in Ebola virus), R301 3.2A (2.9 in Ebola virus) R322 2.8 and 3.0 (both 2.8A in Ebola virus). Also close to A303 across interface, they could compensate or presence of both changes could have greater effect on interface in this area. (6.1A in RESTV, 7.5 in Ebola virus)	probable
V279	A290	Present in a surface loop packs against adjacent helix, conservative change of hydrophobic amino acid. Could be some local conformational changes and is located adjacent to the linker between the two subdomains, which is in RESTV has a short alpha helix that is not present in EBOV.	Unclear
A303	V314	Present in a surface loop near the VP35 dimer interface. Close in space to D258 in the other subunit.	unclear
K318	Q329	Located at the end of a beta sheet. Adjacent to His285 in next strand. His285 is completely conserved in all *Ebolavirus* species. So Reston virus VP35 has increased positive charge in this position	unclear

Supplementary Table 14. Structural analysis of VP35 SDPs. Details of the structural analysis are included with an assessment of whether the amino acid change is likely to have an effect on the protein. Four categories are used for the effect column unlikely (the change seems unlikely to alter the structure/function), unclear (the change could be functional but there is limited evidence), possible (more confident that there is an effect than the unclear group) and probably (highly confident that the change will have a structural/functional effect).

RESTV residue	Pathogenic consensus	Comments	functional effect
I151	T150	The side chain is largely buried and it appears that Reston virus I151 would be tolerated although a hydrogen bond with the backbone of the previous turn of the helix will be lost.	unlikely
R158	Q157	Located in a surface loop, will increase surface charge. It is possible that Reston virus forms a salt bridge with D159, which would increase stability and reduce flexibility in this area of the protein. This SDP is in a region of SDPs and very close to another SDP (I159L). So possible effects may be compensated by other changes.	unlikely
L160	I159	Located in a surface close to another SDP (see above). Appears to be a conservative change that given the other species specific changes in this area it seems unlikely that it will have a functional effect on the protein.	unlikely
H197	R196	Surface residue so change in size/shape should well accommodated, positive charge maintained in side chain.	unlikely
D206	E205	Exposed surface residue, conservative change of amino acid. Unlikely to alter protein structure.	unlikely
A263	R262	This residue is present in the dimer interface. In Ebola virus VP30 R262 hydrogen bonds with the backbone of A141 and G140. Reston virus A263 will be unable to hydrogen bond. This is likely to reduce the affinity of the dimer (given that it is symmetrical and so the Ebola virus R262 in each subunit forms hydrogen bonds with the other subunit. The Reston virus dimer has been observed to be rotated relative to the Ebola virus. The loss of the hydrogen bonds may explain this.	probable

Supplementary Table 15. Structural analysis of VP30 SDPs. Details of the structural analysis are included with an assessment of whether the amino acid change is likely to have an effect on the protein. Four categories are used for the effect column unlikely (the change seems unlikely to alter the structure/function), unclear (the change could be functional but there is limited evidence), possible (more confident that there is an effect than the unclear group) and probably (highly confident that the change will have a structural/functional effect).

Reston virus residue	Pathogenic consensus	Comments	Possible Functional effect
V46	T46	Present in a surface loop (although only third amino acid in structure). Reston virus V46 introduces a hydrophobic amino acid on surface, could affect stability but no evidence for this.	unclear
T85	P85	Ebola virus P85 is in a S-G-P-K beta-turn, proline confers backbone rigidity and change to Thr in Reston virus would introduce backbone flexibility and provide a side chain with H-bond donor. Located in the Ebola virus octamer interface, will result in changes to this interface and likely alter the octamer structure. In an octamer structure (if it were to remain similar to the Ebola virus octamer), T85 could hydrogen bond with the backbone of L117 or the sidechain of R137.	probably
V122	I122	This change appears to be conservative substitution of two hydrophobic amino acids. Ebola virus I122 is packed with other hydrophobic residues and it appears that the region would be able to accommodate the change to Reston virus V122 with a slightly smaller side chain.	unlikely
N201	G201	Located in a surface loop. Based on the Ebola virus structure, the Reston virus N201 side chain would be likely to point into the protein structure. But not clear what effect this would have on the protein structure, if any given that the structure has gaps in this region so cannot be confident.	unclear
L209	F209	Packed in a largely hydrophobic region the SDP results in a reduction in side chain size in Reston virus. The smaller Leucine may adopt different side chain conformations to aid stability. Ebola virus F209 does not interact with other aromatic side chains so the structure is unlikely to be adversely affected by the swap to Leucine. Surrounding hydrophobic residues are aliphatic (I261, I285, V298, A318, P317) so the change to Leucine could be well accommodated.	unlikely
P245	Q245	Located at the end of an alpha helix, the Reston virus P245 would break the helix and shorten it to either L244 or more likely M241, which is a better C-capping residue. This could have a destabilizing effect on the two helices in this region and the base of the hydrophobic core because secondary structure will most likely change to accommodate the inflexible Proline.	probably
Q269	H269	A surface residue, loss of charge to polar side chain. This is a highly charged region with E265, R270, K274, K275. So the positive charge would be reduced in Reston virus VP40.	unclear
V293	I293	Packs with other hydrophobic residues. Appears to be a conservative change	Unlikely

Supplementary Table 16. Structural analysis of VP40 SDPs. Details of the structural analysis are included with an assessment of whether the amino acid change is likely to have an effect on the protein. Four categories are used for the effect column unlikely (the change seems unlikely to alter the structure/function), unclear (the change could be functional but there is limited evidence), possible (more confident that there is an effect than the unclear group) and probably (highly confident that the change will have a structural/functional effect). Analysis is based on the VP40 dimer structure unless otherwise stated.

Reston virus residue	Pathogenic consensus	Comments	Possible functional effect
M17	L17	Located in a helix. Appears to be a conservative change in amino acid. No suggestion from structure that it would alter structure/function.	unlikely
I22	V22	Located in a helix and is fairly tightly packed against the adjacent helix but would expect the pocket to accommodate the change.	unlikely
I31	V31	Located in a sheet facing a loop. Side chain is relatively exposed so structure should be able to accommodate. Adjacent in space to another SDP (132)	unlikely
S131	T131	Ebola virus T131 forms hydrogen bonds with the side chains of T129, W125 and with the backbone of H133. Model of Reston virus VP24 suggests S131 would continue to interact with the same residues. This residue is on the edge of the KPNA5 binding site. Appears to be a conservative change of amino acid.	probable
T132	N132	Exposed polar residue exchanges for another polar residue. Unlikely to affect structure. Adjacent in space to an SDP (V31S) and in sequence to 131.	unlikely
L136	M136	Part of the interface site with KPNA5. Mutagenesis of M136 in combination with other residues resulted in loss of KPNA5 binding[34]. Although it appears to be a conservative substitution.	probable
R139	Q139	Interface residue. In Ebola virus Q139 forms an H bond with the backbone of R137. This is likely to be lost in Reston virus VP24 with the longer R139 side chain. Change will also introduce positive charge at interface site.	probable
A226	T226	Located in a helix facing a sheet. Ebola virus T226 forms a hydrogen bond with the backbone of D48. Reston virus A226 will not be able to form this hydrogen bond. This is likely to reduce the stability of the protein and increase flexibility.	Probable

Supplementary Table 17. Structural analysis of VP24 SDPs. Details of the structural analysis are included with an assessment of whether the amino acid change is likely to have an effect on the protein. Four categories are used for the effect column unlikely (the change seems unlikely to alter the structure/function), unclear (the change could be functional but there is limited evidence), possible (more confident that there is an effect than the unclear group) and probably (highly confident that the change will have a structural/functional effect).

Region	Residue	Conservation
1	L136	SDP
1	R139	SDP
1	S140	Not an SDP but conserved S in Reston viruses and mainly R in Ebola viruses, not conserved enough to be SDP
2	L107	Vary in species specific manner
2	H109	Vary in species specific manner
2	T116	Vary in species specific manner
2	G120	Not an SDP – G in Reston viruses and Ebola viruses (mainly), differs in others
3	S184	
3	T185	Not an SDP. T in Reston viruses, mainly N in other species
3	H186	Vary in species specific manner
3	T187	Not an SDP, primarily T in most species (A in Sudan viruses)
3	F197	Vary in species specific manner
4	V201	Vary in species specific manner
5	S50	Not an SDP

Supplementary Table 18. Residues in VP24 previously identified to differ between Reston viruses and Ebola viruses and/or Sudan viruses. Zhang et al., identified five regions that differed between Reston viruses and Ebola viruses and/or Sudan viruses[7] .The five regions are listed along with conservation information i.e. whether the position is an SDP, varies in a species specific manner (i.e. not an SDP, but a different residue is conserved in each of the different species) or otherwise conserved. Region one is part of the KPNA5 (karyopherin α5) binding site and region two is thought to be part of the STAT1 binding site[7].

Mutation	Location/Comments	Relationship to SDPs
From Volchhkov et al.,[43] – experiment 1		
M71I	Surface residue. Not clear what functional effect would be.	Not close
L147P	Part of an alpha helix, the proline would be expected to break the helix and could lead to conformational changes that would alter function.	Close to SDPs L17M, V22I
T187I	Adjacent to interface site. T187 forms Hydrogen bonds with the backbone of H186 and E203. Mutation to I would remove these hydrogen bonds and reduce stability/increase flexibility in this area. (Also close to L26F mutation from a separate study)	Not close
From Volchhkov et al.,[43] – experiment 2		
H186Y	Present in interface with KPNA5. Forms a hydrogen bond with the backbone of T434 in KPNA5. Mutation to Tyr would still enable Hydrogen bonding with KPNA as the functional group is maintained.	Not close
From Ebihara et al.,[44]		
T50I	The side chain of Ebola virus T50 can hydrogen bond with the backbones of Q36 and K52. Removal of these interactions with mutation Ile will reduce stability/increase flexibility.	Close to SDP T226A
From Dowall et al.,[45]		
L26F	Largely buried side chain. Increase in size to phenylalanine could require some conformational change. Interesting that is located close to T187I (see above).	Close to V22I
F29V*	Largely buried side chain. Reduction in size would create space and therefore likely to result in some conformational change?	Close in space to SDPs T131S, N132T, V31I.
A43P*	Close in space to L26F (see above). Present in a turn.	
K218R*	Appears to be a conservative change. K218 is present in the KPNA5 interface. Is close to M436 and D489. Possible electrostatic interaction. Possible the mutation to R enables this interaction to continue in the different species.	

Supplementary Table 19. VP24 Mutations occurring in adaption of Ebola virus to rodent species. The location of the mutation and how it may alter structure and function is listed with details of proximity to SDPs. *indicates that after passage one the predominant amino acid at that position was the wild type [44]. In the Dowall et al.[45], study L26F is the only mutation where the mutation is predominantly maintained in in all passages. Separate experimental evidence suggests that the L26F mutation along results in pathogenicity in guinea pigs[37].

Genome Identifier	Ebola virus species	Host
gb:KJ660346	Organism:Zaire ebolavirus H.sapiens-wt/GIN/2014/Makona-Kissidougou-C15	Human
gb:KJ660347	Organism:Zaire ebolavirus H.sapiens-wt/GIN/2014/Makona-Gueckedou-C07	Human
gb:KJ660348	Organism:Zaire ebolavirus H.sapiens-wt/GIN/2014/Makona-Gueckedou-C05	Human
gb:KP342330	Organism:Zaire ebolavirus H.sapiens-wt/GIN/2014/Conacry-192	Human
gb:KP096422	Organism:Zaire ebolavirus H.sapiens-tc/GIN/14/WPG-C15	Human
gb:KP096421	Organism:Zaire ebolavirus H.sapiens-tc/GIN/14/WPG-C07	Human
gb:KP096420	Organism:Zaire ebolavirus H.sapiens-tc/GIN/14/WPG-C05	Human
gb:KC242800	Organism:Zaire ebolavirus EBOV/H.sapiens-tc/GAB/2002/Ilembe	Human
gb:KC242794	Organism:Zaire ebolavirus EBOV/H.sapiens-tc/GAB/1996/2Nza	Human
gb:KC242797	Organism:Zaire ebolavirus EBOV/H.sapiens-tc/GAB/1996/1Oba	Human
gb:KC242795	Organism:Zaire ebolavirus EBOV/H.sapiens-tc/GAB/1996/1Mbie	Human
gb:KC242798	Organism:Zaire ebolavirus EBOV/H.sapiens-tc/GAB/1996/1Ikot	Human
gb:KC242793	Organism:Zaire ebolavirus EBOV/H.sapiens-tc/GAB/1996/1Eko	Human
gb:KC242792	Organism:Zaire ebolavirus EBOV/H.sapiens-tc/GAB/1994/Gabon	Human
gb:KC242784	Organism:Zaire ebolavirus EBOV/H.sapiens-tc/COD/2007/9 Luebo	Human
gb:KC242790	Organism:Zaire ebolavirus EBOV/H.sapiens-tc/COD/2007/5 Luebo	Human
gb:KC242788	Organism:Zaire ebolavirus EBOV/H.sapiens-tc/COD/2007/43 Luebo	Human
gb:KC242789	Organism:Zaire ebolavirus EBOV/H.sapiens-tc/COD/2007/4 Luebo	Human
gb:KC242787	Organism:Zaire ebolavirus EBOV/H.sapiens-tc/COD/2007/23 Luebo	Human
gb:KC242786	Organism:Zaire ebolavirus EBOV/H.sapiens-tc/COD/2007/1 Luebo	Human
gb:KC242785	Organism:Zaire ebolavirus EBOV/H.sapiens-tc/COD/2007/0 Luebo	Human
gb:KC242799	Organism:Zaire ebolavirus EBOV/H.sapiens-tc/COD/1995/13709 Kikwit	Human
gb:KC242796	Organism:Zaire ebolavirus EBOV/H.sapiens-tc/COD/1995/13625 Kikwit	Human
gb:KC242791	Organism:Zaire ebolavirus EBOV/H.sapiens-tc/COD/1977/Bonduni	Human
gb:KC242801	Organism:Zaire ebolavirus EBOV/H.sapiens-tc/COD/1976/deRoover	Human
gb:KM233118	Organism:Zaire ebolavirus Ebola virus/H.sapiens-wt/SLE/2014/Makona-NM042.3	Human
gb:KM233117	Organism:Zaire ebolavirus Ebola virus/H.sapiens-wt/SLE/2014/Makona-NM042.2	Human
gb:KM233116	Organism:Zaire ebolavirus Ebola virus/H.sapiens-wt/SLE/2014/Makona-NM042.1	Human
gb:KM233115	Organism:Zaire ebolavirus Ebola virus/H.sapiens-wt/SLE/2014/Makona-G3857	Human
gb:KM233114	Organism:Zaire ebolavirus Ebola virus/H.sapiens-wt/SLE/2014/Makona-G3856.3	Human
gb:KM233113	Organism:Zaire ebolavirus Ebola virus/H.sapiens-wt/SLE/2014/Makona-G3856.1	Human
gb:KM233112	Organism:Zaire ebolavirus Ebola virus/H.sapiens-wt/SLE/2014/Makona-G3851	Human
gb:KM233111	Organism:Zaire ebolavirus Ebola virus/H.sapiens-wt/SLE/2014/Makona-G3850	Human
gb:KM233110	Organism:Zaire ebolavirus Ebola virus/H.sapiens-wt/SLE/2014/Makona-G3848	Human
gb:KM233109	Organism:Zaire ebolavirus Ebola virus/H.sapiens-wt/SLE/2014/Makona-G3846	Human
gb:KM233108	Organism:Zaire ebolavirus Ebola virus/H.sapiens-wt/SLE/2014/Makona-G3845	Human
gb:KM233107	Organism:Zaire ebolavirus Ebola virus/H.sapiens-wt/SLE/2014/Makona-G3841	Human
gb:KM233106	Organism:Zaire ebolavirus Ebola virus/H.sapiens-wt/SLE/2014/Makona-G3840	Human
gb:KM233105	Organism:Zaire ebolavirus Ebola virus/H.sapiens-wt/SLE/2014/Makona-G3838	Human
gb:KM233104	Organism:Zaire ebolavirus Ebola virus/H.sapiens-wt/SLE/2014/Makona-G3834	Human
gb:KM233103	Organism:Zaire ebolavirus Ebola virus/H.sapiens-wt/SLE/2014/Makona-G3831	Human
gb:KM233102	Organism:Zaire ebolavirus Ebola virus/H.sapiens-wt/SLE/2014/Makona-G3829	Human
gb:KM233101	Organism:Zaire ebolavirus Ebola virus/H.sapiens-wt/SLE/2014/Makona-G3827	Human
gb:KM233100	Organism:Zaire ebolavirus Ebola virus/H.sapiens-wt/SLE/2014/Makona-G3826	Human
gb:KM233099	Organism:Zaire ebolavirus Ebola virus/H.sapiens-wt/SLE/2014/Makona-G3825.2	Human
gb:KM233098	Organism:Zaire ebolavirus Ebola virus/H.sapiens-wt/SLE/2014/Makona-G3825.1	Human
gb:KM233097	Organism:Zaire ebolavirus Ebola virus/H.sapiens-wt/SLE/2014/Makona-G3823	Human
gb:KM233096	Organism:Zaire ebolavirus Ebola virus/H.sapiens-wt/SLE/2014/Makona-G3822	Human
gb:KM233095	Organism:Zaire ebolavirus Ebola virus/H.sapiens-wt/SLE/2014/Makona-G3821	Human
gb:KM233094	Organism:Zaire ebolavirus Ebola virus/H.sapiens-wt/SLE/2014/Makona-G3820	Human
gb:KM233093	Organism:Zaire ebolavirus Ebola virus/H.sapiens-wt/SLE/2014/Makona-G3819	Human
gb:KM233092	Organism:Zaire ebolavirus Ebola virus/H.sapiens-wt/SLE/2014/Makona-G3818	Human

gb:KM233091	Organism:Zaire ebolavirus Ebola virus/H.sapiens-wt/SLE/2014/Makona-G3817	Human
gb:KM233090	Organism:Zaire ebolavirus Ebola virus/H.sapiens-wt/SLE/2014/Makona-G3816	Human
gb:KM233089	Organism:Zaire ebolavirus Ebola virus/H.sapiens-wt/SLE/2014/Makona-G3814	Human
gb:KM233088	Organism:Zaire ebolavirus Ebola virus/H.sapiens-wt/SLE/2014/Makona-G3810.2	Human
gb:KM233087	Organism:Zaire ebolavirus Ebola virus/H.sapiens-wt/SLE/2014/Makona-G3810.1	Human
gb:KM233086	Organism:Zaire ebolavirus Ebola virus/H.sapiens-wt/SLE/2014/Makona-G3809	Human
gb:KM233085	Organism:Zaire ebolavirus Ebola virus/H.sapiens-wt/SLE/2014/Makona-G3808	Human
gb:KM233084	Organism:Zaire ebolavirus Ebola virus/H.sapiens-wt/SLE/2014/Makona-G3807	Human
gb:KM233083	Organism:Zaire ebolavirus Ebola virus/H.sapiens-wt/SLE/2014/Makona-G3805.2	Human
gb:KM233082	Organism:Zaire ebolavirus Ebola virus/H.sapiens-wt/SLE/2014/Makona-G3805.1	Human
gb:KM233081	Organism:Zaire ebolavirus Ebola virus/H.sapiens-wt/SLE/2014/Makona-G3800	Human
gb:KM233080	Organism:Zaire ebolavirus Ebola virus/H.sapiens-wt/SLE/2014/Makona-G3799	Human
gb:KM233079	Organism:Zaire ebolavirus Ebola virus/H.sapiens-wt/SLE/2014/Makona-G3798	Human
gb:KM233078	Organism:Zaire ebolavirus Ebola virus/H.sapiens-wt/SLE/2014/Makona-G3796	Human
gb:KM233077	Organism:Zaire ebolavirus Ebola virus/H.sapiens-wt/SLE/2014/Makona-G3795	Human
gb:KM233076	Organism:Zaire ebolavirus Ebola virus/H.sapiens-wt/SLE/2014/Makona-G3789.1	Human
gb:KM233075	Organism:Zaire ebolavirus Ebola virus/H.sapiens-wt/SLE/2014/Makona-G3788	Human
gb:KM233074	Organism:Zaire ebolavirus Ebola virus/H.sapiens-wt/SLE/2014/Makona-G3787	Human
gb:KM233073	Organism:Zaire ebolavirus Ebola virus/H.sapiens-wt/SLE/2014/Makona-G3786	Human
gb:KM233072	Organism:Zaire ebolavirus Ebola virus/H.sapiens-wt/SLE/2014/Makona-G3782	Human
gb:KM233071	Organism:Zaire ebolavirus Ebola virus/H.sapiens-wt/SLE/2014/Makona-G3771	Human
gb:KM233070	Organism:Zaire ebolavirus Ebola virus/H.sapiens-wt/SLE/2014/Makona-G3770.2	Human
gb:KM233069	Organism:Zaire ebolavirus Ebola virus/H.sapiens-wt/SLE/2014/Makona-G3770.1	Human
gb:KM233068	Organism:Zaire ebolavirus Ebola virus/H.sapiens-wt/SLE/2014/Makona-G3769.4	Human
gb:KM233067	Organism:Zaire ebolavirus Ebola virus/H.sapiens-wt/SLE/2014/Makona-G3769.3	Human
gb:KM233066	Organism:Zaire ebolavirus Ebola virus/H.sapiens-wt/SLE/2014/Makona-G3769.2	Human
gb:KM233065	Organism:Zaire ebolavirus Ebola virus/H.sapiens-wt/SLE/2014/Makona-G3769.1	Human
gb:KM233064	Organism:Zaire ebolavirus Ebola virus/H.sapiens-wt/SLE/2014/Makona-G3765.2	Human
gb:KM233063	Organism:Zaire ebolavirus Ebola virus/H.sapiens-wt/SLE/2014/Makona-G3764	Human
gb:KM233062	Organism:Zaire ebolavirus Ebola virus/H.sapiens-wt/SLE/2014/Makona-G3758	Human
gb:KM233061	Organism:Zaire ebolavirus Ebola virus/H.sapiens-wt/SLE/2014/Makona-G3752	Human
gb:KM233060	Organism:Zaire ebolavirus Ebola virus/H.sapiens-wt/SLE/2014/Makona-G3750.3	Human
gb:KM233059	Organism:Zaire ebolavirus Ebola virus/H.sapiens-wt/SLE/2014/Makona-G3750.2	Human
gb:KM233058	Organism:Zaire ebolavirus Ebola virus/H.sapiens-wt/SLE/2014/Makona-G3750.1	Human
gb:KM233057	Organism:Zaire ebolavirus Ebola virus/H.sapiens-wt/SLE/2014/Makona-G3735.2	Human
gb:KM233056	Organism:Zaire ebolavirus Ebola virus/H.sapiens-wt/SLE/2014/Makona-G3735.1	Human
gb:KM233055	Organism:Zaire ebolavirus Ebola virus/H.sapiens-wt/SLE/2014/Makona-G3734.1	Human
gb:KM233054	Organism:Zaire ebolavirus Ebola virus/H.sapiens-wt/SLE/2014/Makona-G3729	Human
gb:KM233053	Organism:Zaire ebolavirus Ebola virus/H.sapiens-wt/SLE/2014/Makona-G3724	Human
gb:KM233052	Organism:Zaire ebolavirus Ebola virus/H.sapiens-wt/SLE/2014/Makona-G3713.4	Human
gb:KM233051	Organism:Zaire ebolavirus Ebola virus/H.sapiens-wt/SLE/2014/Makona-G3713.3	Human
gb:KM233050	Organism:Zaire ebolavirus Ebola virus/H.sapiens-wt/SLE/2014/Makona-G3713.2	Human
gb:KM233049	Organism:Zaire ebolavirus Ebola virus/H.sapiens-wt/SLE/2014/Makona-G3707	Human
gb:KM034563	Organism:Zaire ebolavirus Ebola virus/H.sapiens-wt/SLE/2014/Makona-G3687.1	Human
gb:KM034562	Organism:Zaire ebolavirus Ebola virus/H.sapiens-wt/SLE/2014/Makona-G3686.1	Human
gb:KM034561	Organism:Zaire ebolavirus Ebola virus/H.sapiens-wt/SLE/2014/Makona-G3683.1	Human
gb:KM034560	Organism:Zaire ebolavirus Ebola virus/H.sapiens-wt/SLE/2014/Makona-G3682.1	Human
gb:KM034559	Organism:Zaire ebolavirus Ebola virus/H.sapiens-wt/SLE/2014/Makona-G3680.1	Human
gb:KM034558	Organism:Zaire ebolavirus Ebola virus/H.sapiens-wt/SLE/2014/Makona-G3679.1	Human
gb:KM034557	Organism:Zaire ebolavirus Ebola virus/H.sapiens-wt/SLE/2014/Makona-G3677.2	Human
gb:KM034556	Organism:Zaire ebolavirus Ebola virus/H.sapiens-wt/SLE/2014/Makona-G3677.1	Human
gb:KM034555	Organism:Zaire ebolavirus Ebola virus/H.sapiens-wt/SLE/2014/Makona-G3676.2	Human
gb:KM034554	Organism:Zaire ebolavirus Ebola virus/H.sapiens-wt/SLE/2014/Makona-G3676.1	Human

gb:KM034553	Organism:Zaire ebolavirus Ebola virus/H.sapiens-wt/SLE/2014/Makona-G3670.1	Human
gb:KM233048	Organism:Zaire ebolavirus Ebola virus/H.sapiens-wt/SLE/2014/Makona-EM124.4	Human
gb:KM233047	Organism:Zaire ebolavirus Ebola virus/H.sapiens-wt/SLE/2014/Makona-EM124.3	Human
gb:KM233046	Organism:Zaire ebolavirus Ebola virus/H.sapiens-wt/SLE/2014/Makona-EM124.2	Human
gb:KM233045	Organism:Zaire ebolavirus Ebola virus/H.sapiens-wt/SLE/2014/Makona-EM124.1	Human
gb:KM233044	Organism:Zaire ebolavirus Ebola virus/H.sapiens-wt/SLE/2014/Makona-EM121	Human
gb:KM233043	Organism:Zaire ebolavirus Ebola virus/H.sapiens-wt/SLE/2014/Makona-EM120	Human
gb:KM233042	Organism:Zaire ebolavirus Ebola virus/H.sapiens-wt/SLE/2014/Makona-EM119	Human
gb:KM233041	Organism:Zaire ebolavirus Ebola virus/H.sapiens-wt/SLE/2014/Makona-EM115	Human
gb:KM233040	Organism:Zaire ebolavirus Ebola virus/H.sapiens-wt/SLE/2014/Makona-EM113	Human
gb:KM233039	Organism:Zaire ebolavirus Ebola virus/H.sapiens-wt/SLE/2014/Makona-EM112	Human
gb:KM233038	Organism:Zaire ebolavirus Ebola virus/H.sapiens-wt/SLE/2014/Makona-EM111	Human
gb:KM233037	Organism:Zaire ebolavirus Ebola virus/H.sapiens-wt/SLE/2014/Makona-EM110	Human
gb:KM233036	Organism:Zaire ebolavirus Ebola virus/H.sapiens-wt/SLE/2014/Makona-EM106	Human
gb:KM233035	Organism:Zaire ebolavirus Ebola virus/H.sapiens-wt/SLE/2014/Makona-EM104	Human
gb:KM034552	Organism:Zaire ebolavirus Ebola virus/H.sapiens-wt/SLE/2014/Makona-EM098	Human
gb:KM034551	Organism:Zaire ebolavirus Ebola virus/H.sapiens-wt/SLE/2014/Makona-EM096	Human
gb:KM034549	Organism:Zaire ebolavirus Ebola virus/H.sapiens-wt/SLE/2014/Makona-EM095B	Human
gb:KM034550	Organism:Zaire ebolavirus Ebola virus/H.sapiens-wt/SLE/2014/Makona-EM095	Human
gb:KP178538	Organism:Zaire ebolavirus Ebola virus/H.sapiens-wt/LBR/2014/Makona-201403007	Human
gb:KP120616	Organism:Zaire ebolavirus Ebola virus/H.sapiens-wt/GBR/2014/Makona-UK1	Human
gb:KP271020	Organism:Zaire ebolavirus Ebola virus/H.sapiens-wt/COD/2014/Lomela-Lokolia19	Human
gb:KP271018	Organism:Zaire ebolavirus Ebola virus/H.sapiens-wt/COD/2014/Lomela-Lokolia16	Human
gb:KP728283	Organism:Zaire ebolavirus Ebola virus/H.sapiens-wt/CHE/2014/Makona-GE1	Human
gb:KP701371	Organism:Zaire ebolavirus Ebola virus/H.sapiens-tc/SLE/2014/Makona-Italy-INMI1	Human
gb:KP184503	Organism:Zaire ebolavirus Ebola virus/H.sapiens-tc/GBR/2014/Makona-UK1.1	Human
gb:KM655246	Organism:Zaire ebolavirus Ebola virus/H.sapiens-tc/COD/1976/Yambuku-Ecran	Human
gb:KP260802	Organism:Zaire ebolavirus Ebola virus H.sapiens/MLI/14/Manoka-Mali-DPR4	Human
gb:KP260801	Organism:Zaire ebolavirus Ebola virus H.sapiens/MLI/14/Manoka-Mali-DPR3	Human
gb:KP260800	Organism:Zaire ebolavirus Ebola virus H.sapiens/MLI/14/Manoka-Mali-DPR2	Human
gb:KP260799	Organism:Zaire ebolavirus Ebola virus H.sapiens/MLI/14/Manoka-Mali-DPR1	Human
gb:NC_002549	Organism:Zaire ebolavirus Ebola virus H.sapiens-tc/COD/1976/Yambuku-Mayinga	Unknown
gb:AY354458	Organism:Zaire ebolavirus Zaire 1995	Unknown
gb:JA489037	Organism:Zaire ebolavirus UNKNOWN-JA489037	Unknown
gb:HC874683	Organism:Zaire ebolavirus UNKNOWN-HC874683	
gb:HC874681	Organism:Zaire ebolavirus UNKNOWN-HC874681	
gb:HC874677	Organism:Zaire ebolavirus UNKNOWN-HC874677	
gb:HC874665	Organism:Zaire ebolavirus UNKNOWN-HC874665	
gb:HC874661	Organism:Zaire ebolavirus UNKNOWN-HC874661	
gb:HC069241	Organism:Zaire ebolavirus UNKNOWN-HC069241	
gb:HC069239	Organism:Zaire ebolavirus UNKNOWN-HC069239	
gb:HC069235	Organism:Zaire ebolavirus UNKNOWN-HC069235	
gb:HC069221	Organism:Zaire ebolavirus UNKNOWN-HC069221	
gb:HC069217	Organism:Zaire ebolavirus UNKNOWN-HC069217	
gb:KF827427	Organism:Zaire ebolavirus rec/COD/1976/Mayinga-rgEBOV	Human
gb:AF272001	Organism:Zaire ebolavirus Mayinga	Guinea Pig
gb:AF499101	Organism:Zaire ebolavirus Mayinga	Guinea Pig
gb:AY142960	Organism:Zaire ebolavirus Mayinga	Guinea Pig
gb:EU224440	Organism:Zaire ebolavirus Mayinga	Guinea Pig
gb:AF086833	Organism:Zaire ebolavirus Mayinga	Guinea Pig
gb:JQ352763	Organism:Zaire ebolavirus Kikwit	Unknown
gb:JA489027	Organism:Tai Forest ebolavirus UNKNOWN-JA489027	Unknown
gb:FJ217162	Organism:Tai Forest ebolavirus UNKNOWN-FJ217162	Human

gb:NC_014372	Organism:Tai Forest ebolavirus Tai Forest virus/H.sapiens-tc/CIV/1994/Pauleoula-CI	Human
gb:EU338380	Organism:Sudan ebolavirus Yambio	Human
gb:HC874655	Organism:Sudan ebolavirus UNKNOWN-HC874655	
gb:HC069211	Organism:Sudan ebolavirus UNKNOWN-HC069211	
gb:KC242783	Organism:Sudan ebolavirus SUDV/H.sapiens-tc/SSD/1979/Maleo	Human
gb:NC_006432	Organism:Sudan ebolavirus Sudan virus/H.sapiens-tc/UGA/2000/Gulu-808892	Unknown
gb:JN638998	Organism:Sudan ebolavirus Sudan	Human
gb:AY729654	Organism:Sudan ebolavirus Gulu	Unknown
gb:KC545392	Organism:Sudan ebolavirus EboSud-682 2012	Human
gb:KC589025	Organism:Sudan ebolavirus EboSud-639	Human
gb:KC545391	Organism:Sudan ebolavirus EboSud-609 2012	Human
gb:KC545390	Organism:Sudan ebolavirus EboSud-603 2012	Human
gb:KC545389	Organism:Sudan ebolavirus EboSud-602 2012	Human
gb:FJ968794	Organism:Sudan ebolavirus Boniface	Unknown
gb:HC874675	Organism:Reston ebolavirus UNKNOWN-HC874675	
gb:HC874663	Organism:Reston ebolavirus UNKNOWN-HC874663	
gb:HC874659	Organism:Reston ebolavirus UNKNOWN-HC874659	
gb:HC874657	Organism:Reston ebolavirus UNKNOWN-HC874657	
gb:HC069233	Organism:Reston ebolavirus UNKNOWN-HC069233	
gb:HC069219	Organism:Reston ebolavirus UNKNOWN-HC069219	
gb:HC069215	Organism:Reston ebolavirus UNKNOWN-HC069215	
gb:HC069213	Organism:Reston ebolavirus UNKNOWN-HC069213	
gb:JX477165	Organism:Reston ebolavirus Reston09-A	Swine
gb:FJ621585	Organism:Reston ebolavirus Reston08-E	Swine
gb:FJ621584	Organism:Reston ebolavirus Reston08-C	Swine
gb:FJ621583	Organism:Reston ebolavirus Reston08-A	Swine
gb:NC_004161	Organism:Reston ebolavirus Reston virus/M.fascicularis-tc/USA/1989/Philippines89- Pennsylvania	Unknown
gb:AB050936	Organism:Reston ebolavirus Reston	
gb:AF522874	Organism:Reston ebolavirus Pennsylvania	
gb:AY769362	Organism:Reston ebolavirus Pennsylvania	
gb:JX477166	Organism:Reston ebolavirus Alice, TX USA MkCQ8167	Monkey
gb:NC_014373	Organism:Bundibugyo virus Bundibugyo virus/H.sapiens-tc/UGA/2007/Butalya-811250	Human
gb:JA489018	Organism:Bundibugyo ebolavirus UNKNOWN-JA489018	Unknown
gb:FJ217161	Organism:Bundibugyo ebolavirus UNKNOWN-FJ217161	Human
gb:KC545396	Organism:Bundibugyo ebolavirus EboBund-14 2012	Human
gb:KC545395	Organism:Bundibugyo ebolavirus EboBund-122 2012	Human
gb:KC545394	Organism:Bundibugyo ebolavirus EboBund-120 2012	Human
gb:KC545393	Organism:Bundibugyo ebolavirus EboBund-112 2012	Human

Supplementary Table 20. Information on the 196 complete *Ebolavirus* genomes. Genomes were downloaded from Virus Pathogen Resource, VIPR (http://www.viprbrc.org/brc/home.spg?decorator=vipr) .

**Protein**	**Effective number of sequences**	**Effective number of human pathogenic sequence**	**Effective number of Reston virus sequences**
GP	95.15	86	4
L	99.2	78	7
NP	148.96	133	7
VP24	88.2	79	7
VP30	96.04	84	7
VP35	99.96	87	7
VP40	90.16	80	7

Supplementary Table 21. Effective number of independent sequences in the dataset. The effective number of independent sequences present in the multiple sequence alignments for each of the Ebolavirus proteins is shown. Values were calculated using hmmer (see material and methods).
